# Concerted genomic and epigenomic changes accompany stabilization of *Arabidopsis* allopolyploids

**DOI:** 10.1038/s41559-021-01523-y

**Published:** 2021-08-19

**Authors:** Xinyu Jiang, Qingxin Song, Wenxue Ye, Z. Jeffrey Chen

**Affiliations:** 1 State Key Laboratory of Crop Genetics and Germplasm Enhancement, Nanjing Agricultural University, Nanjing, China; 2 Department of Molecular Biosciences, The University of Texas at Austin, Austin, TX USA

**Keywords:** Epigenomics, Evolutionary genetics, Polyploidy in plants

## Abstract

During evolution successful allopolyploids must overcome ‘genome shock’ between hybridizing species but the underlying process remains elusive. Here, we report concerted genomic and epigenomic changes in resynthesized and natural *Arabidopsis suecica* (TTAA) allotetraploids derived from *Arabidopsis* *thaliana* (TT) and *Arabidopsis* *arenosa* (AA). *A. suecica* shows conserved gene synteny and content with more gene family gain and loss in the A and T subgenomes than respective progenitors, although *A. arenosa*-derived subgenome has more structural variation and transposon distributions than *A. thaliana*-derived subgenome. These balanced genomic variations are accompanied by pervasive convergent and concerted changes in DNA methylation and gene expression among allotetraploids. The A subgenome is hypomethylated rapidly from F_1_ to resynthesized allotetraploids and convergently to the T-subgenome level in natural *A. suecica*, despite many other methylated loci being inherited from F_1_ to all allotetraploids. These changes in DNA methylation, including small RNAs, in allotetraploids may affect gene expression and phenotypic variation, including flowering, silencing of self-incompatibility and upregulation of meiosis- and mitosis-related genes. In conclusion, concerted genomic and epigenomic changes may improve stability and adaptation during polyploid evolution.

## Abstract (web summary)

*Arabidopsis suecica* is an allotetraploid derived from *Arabidopsis* *thaliana* and *Arabidopsis* *arenosa*. Analysis of resynthesized and natural allotetraploid *A. suecica* shows balanced genomic variation accompanied by convergent and concerted changes in DNA methylation and gene expression between two subgenomes that probably contributed to genome stability during polyploid evolution.

## Main

Polyploidy or whole-genome duplication (WGD) is a pervasive feature of genome evolution in animals and flowering plants^1–6^. Many important crops are allopolyploids, such as wheat, cotton and canola and autopolyploids including alfalfa and potato. Many other plants, such as *Arabidopsis thaliana* and maize, are palaeopolyploids that underwent one or more rounds of WGD during evolution. The common occurrence of polyploidy suggests advantages for polyploids to possess genomic diversity, gene expression and epigenetic changes in response to selection, adaptation and domestication^1,2,6,7^. Notably, many newly resynthesized or naturally formed allotetraploids have experienced ‘genome shock’^8^, including rapid genomic reshuffling as observed in *Brassica napus*^9^ and *Tragopogon miscellus*^10^, while others, such as *A. suecica*^11–13^ and cotton (*Gossypium*) allotetraploids^14^, show genomic stability and conservation. The basis for this paradox between rapid genomic reshuffling and relatively stable genomes among different allopolyploids is unknown.

*Arabidopsis* is a powerful model for studying plant biology and polyploid evolution, consisting of diploids (for example, *A. thaliana*, Ath), autotetraploids (*A. arenosa*, Aar and *A. lyrata*, Aly) and allotetraploids such as *A. suecica* (Asu)^15^ and *A. kamchatica* (Aka); the latter was formed between *A. lyrata* and *A. halleri* (Aha)^16^. Asu (AATT, 2*n* = 4*x* = 26) was formed naturally^15^ and can also be resynthesized by pollinating tetraploid Ath Ler4 ecotype (TTTT, 2*n* = 4*x* = 20) with Aar (AAAA, 2*n* = 4*x* = 32) pollen, generating two independent and genetically stable strains (Allo733 and Allo738)^11,12^. Consistently, A subgenome of natural *A. suecica* is reported to be more closely related to tetraploid than diploid *A. arenosa*^17^. Resynthesized and natural *A. suecica* provides a powerful model for studying genetic and epigenetic changes in morphological evolution, non-additive gene expression, nucleolar dominance and hybrid vigour^7,11–13,18–22^. However, despite genomes of 1,135 *A. thaliana*^23^ and several related species, including Aly^24^, Aha^25^ and Aka^26^, having been sequenced, *A. arenosa* and *A. suecica* genomes are unavailable, except for a draft sequence of Asu^13^.

Here, we report high-quality sequences of both Ath Ler and Aar genomes in resynthesized allotetraploids and two subgenomes of natural *A. suecica*. Using these sequences, we studied genomic variation, DNA methylation and gene expression changes between the progenitors and their related subgenomes in resynthesized and natural *A. suecica*. Our findings indicate that balanced genomic diversifications in allotetraploids are accompanied by convergent and concerted changes in DNA methylation and gene expression between two subgenomes. This example of genomic and epigenomic reconciliation may provide a basis for stabilizing subgenomic structure and function to improve adaptation during polyploid evolution.

## Results

### Sequences, assemblies and annotation of *A. suecica* and *A. arenosa* genomes

**Fig. 1:**
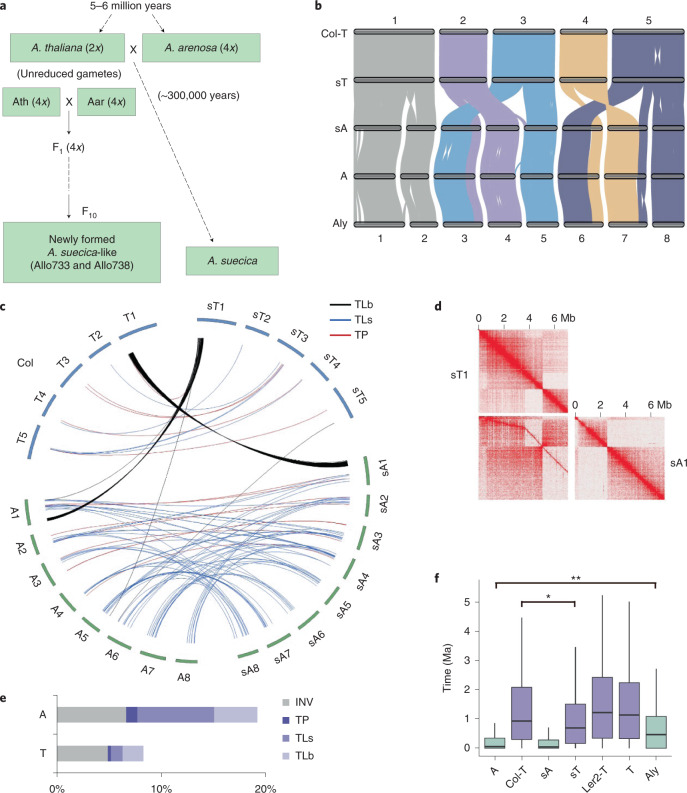
Conservation and diversification of *A. suecica* genome. **a**, Diagram of resynthesized allotetraploids and natural *A. suecica* (Asu). Allo733 and Allo738 are two stable *A. suecica*-like allotetraploids derived from tetraploid *A. thaliana* (Ath, Ler4) and *A. arenosa* (Aar, Care-1)^11^. Natural Asu was formed ~300,000 years ago. **b**, Genomic synteny of *A. thaliana* Col genome (Col-T), A (Aar-related) subgenome of Allo738, sT and sA subgenomes of Asu and *A. lyrata* (Aly). Syntenic blocks with 30 or more genes are shown. **c**, Rearrangements between sT (1–5) and sA (1–8) subgenomes of natural Asu and extant progenitors, *A. thaliana* (Col, T1–T5) and *A. arenosa* (A subgenome of Allo738, A1–A8). Ribbons indicate translocation between subgenomes (TLb, black), within a subgenome (TLs, blue) and transposition in the same chromosomes (TP, red). **d**, A large (~5 Mb) translocation is present between sT1 and sA1 relative to T1 and A1 chromosomes, which was validated by chromatin contact (Hi-C) maps. **e**, Proportion of sequence variation in sA and sT subgenomes of Asu relative to extant progenitors. INV, inversion; TP, transpositions; TLs, translocations within a subgenome; TLb, translocations between subgenomes. **f**, Boxplots of the estimated time for intact LTR insertion (million years ago, Ma) in A (Aar-related) subgenome of 738, Col genome (Col-T), sT and sA subgenomes of Asu, diploid Ler genome (Ler2-T), T (Ler4) subgenome of Allo738 and *A. lyrata* (Aly) genome. Single and double asterisks indicate statistical significance levels of *P* < 0.05 and 0.01, respectively (permutation test using 1,000 permutations). Source data

**Extended Data Fig. 1:**
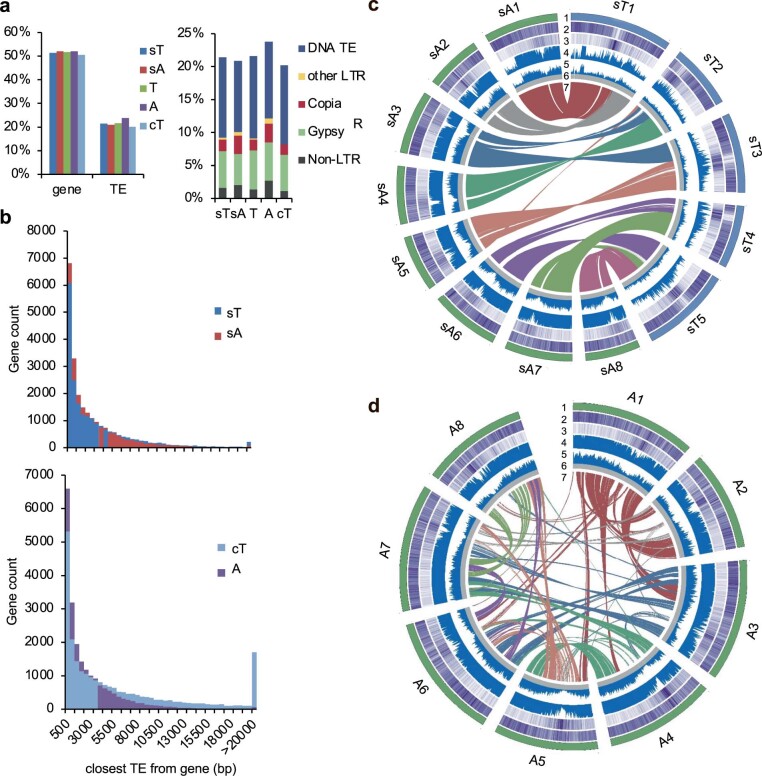
Synteny and gene content of two subgenome in *A. suecica*. **a**, Proportion of genes and TEs (left) and TE classes (right) in *A. thaliana* (Col) and in A and T subgenomes of Allo738 (738) and sA and sT subgenome of *A. suecica* (Asu). **b**, Distances of the nearest TE from each gene in sT and sA subgenomes of Asu (upper panel) and in Col (cT) and A subgenome (Aar-related) of Allo738 (lower panel). **c**, **d**, Genomic features of *A. suecica* (**c**) and *A. arenosa* (**d**) genome. (1) Chromosome ideogram; (2) Gene density; (3) TE density; (4)-(6) SNP (4), indel (5) identity, and aligned regions (6) between *A. suecica* and Col or A (**c**) or between A and *A. lyrata* genomes (**d**); (7) synteny of sA and sT homologous gene pairs (**c**) or paralogous gene pairs (**d**). Colours in (1) indicate T (blue) and A (green) or related subgenomes; colour scales in (2) and (3) indicate high (dark purple) to low (white) density; densities (2)-(5) were shown per 100-kb windows; only gene pairs in syntenic blocks spanning 30 genes were shown in (7). Source data

**Extended Data Fig. 2:**
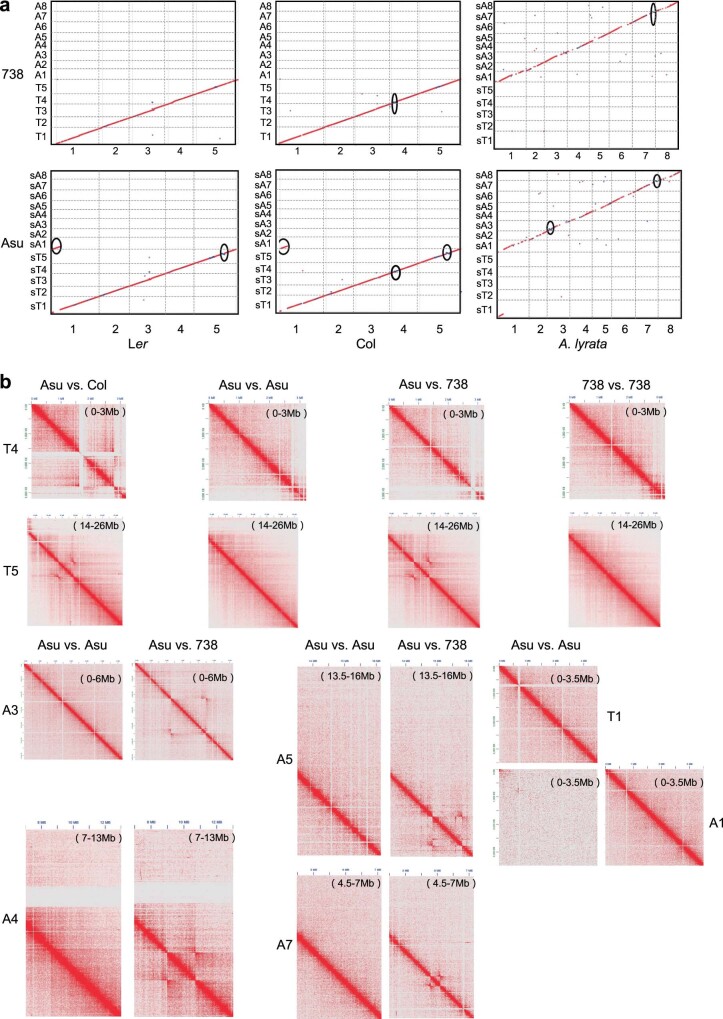
Analysis of Allo733 and A. suecica genome assemblies using reference genomes. **a**, Dotplots of allotetraploid Allo738 (738) and *A. suecica* (Asu) assemblies with reference genomes of *A. thaliana* (L*er* and Col) and *A. lyrata*, respectively. Two genomes were co-linear (red line) with disruptions (blue lines or dots) and inversions (blue line) or translocations (black circles). **b**, Heatmaps of Hi-C-seq chromosome contacts to show the location of structural variation in different chromosomes. Source data

*A. arenosa* is obligately outcrossing and highly heterozygous^12^. To overcome the heterozygosity issue, we sequenced the genome of a resynthesized allotetraploid, Allo738, that had been maintained by self-pollination for ten or more generations (Fig. 1a)^11,19,27^. In addition, we sequenced natural allotetraploid *A. suecica* (Asu) that was formed 14,000–300,000 years ago^13,28^. Here, we adopted chromosome nomenclatures, T1–T5 (T subgenome) and A1–A8 (A subgenome) for resynthesized allotetraploids (Allo733 and Allo738) and sT1–sT5 and sA1–sA8 for natural *A. suecica*, while Col, Ler2 (diploid) and Ler4 (tetraploid), Aar and Asu were used to specify individual genomes. The genomes were assembled de novo using integrated sequencing approaches of single-molecule real-time (PacBio Sequel, ~130×), paired-end (Illumina HiSeq, ~80×) and chromosome conformation capture (Hi-C, ~80×) (Methods). Genome sizes of *A. suecica* and Allo738 were estimated to be 272.4 and 269.2 megabases (Mb), respectively, of which 96.9 and 98.6% were represented in the 13 largest scaffolds, including 120.9–121.1 Mb among five chromosomes (T1–T5) in T or sT subgenome and 147.4–150.6 Mb among eight chromosomes (A1–A8) in A or sA subgenome (Table 1 and Extended Data Fig. 1). Completeness and continuity of these genomes were supported by BUSCO scores^29^ of 95.9–99.2%, although *A. suecica* genome was estimated to be ~345 Mb by flow cytometry^30,31^. *A. suecica* subgenomes were aligned colinearly with gold-standard genomes of *A. thaliana*^23,32^ and *A. lyrata*^24^, respectively, except for several known inversions on chromosomes sT4, sA3, sA7 and a new inversion on chromosome sT5, all of which were confirmed by Hi-C contact matrix analysis (Extended Data Fig. 2a,b). Approximately 50% of the genome is in genic regions including 54,861–55,534 annotated genes and ~20% consists of repetitive sequences including a variety of transposable elements (TEs) (Table 1 and Extended Data Fig. 1a). Many TEs were closely associated with genes and the nearest TEs from genes were closer in A-related than in T-related genomes (Extended Data Fig. 1b).

**Table 1:** Genome assembly and annotation statistics of two *Arabidopsis* allotetraploids

Sequence statistics	*A. suecica* (T + A)	Allo738 (T + A)
Total length of contigs (bp)	272,218,784	268,958,675
Total length of assemblies (bp)	272,391,284	269,147,175
Length of largest 13 super-scaffolds	263,860,340	265,394,178
Percentage of anchored (bp)	96.90%	98.60%
Number of contigs	380	470
Contig L50 (bp)	6,555,646	6,799,294
Number of scaffolds	269	218
Scaffold L50 (bp)	19,847,963	19,689,293
Total length of assemblies (A) (bp)	150,632,036	147,419,868
Total length of assemblies (T) (bp)	120,857,189	121,174,287
Percentage of repeat sequences (A)	26.1	25.0
Percentage of repeat sequences (T)	22.9	23.0
Number (%) of TEs (A)^a^	60,716 (20.9)	68,541 (23.8)
Number (%) of TEs (T)^a^	35,893 (21.4)	36,669 (21.6)
Number of genes (A)^a^	28,945 + 341	27,939 + 288
Number of genes (T)^a^	25,834 + 316	26,553 + 73
Complete BUSCOs (%) (A)	1,602 (99.2)	1,548 (95.9)
Complete BUSCOs (%) (T)	1,589 (98.5)	1,601 (99.2)

Note: Allo738 has *A. thaliana* (Ler) and *A. arenosa* genomes for ten or more generations^11,27^, while the T (*A. thaliana* equivalent) and A (*A. arenosa* equivalent) subgenomes have evolved in Asu for 14,000–300,000 yr (refs. ^13,28^). ^a^Excludes those TEs and genes in unassembled scaffolds.

To test genome stability, we sequenced another neo-allotetraploid, Allo733 (Supplementary Fig. 1), and compared Allo733 and Allo738 genomes with Ler^33^ and other *Arabidopsis* species^13,17^ including *A. arenosa* (Aar) accession^34^. These data suggest that the newly assembled T subgenome of neo-allotetraploids is almost identical to the published Ler sequence and A subgenome is closely related to the *A. arenosa* sequence (Supplementary Information). For this study, we used A and T subgenomes of Allo738 (and Allo733) as *A. arenosa* (Aar) and *A. thaliana* (Ath, Ler) genomes, respectively, for further analysis.

### Genomic diversity between progenitors and subgenomes

**Extended Data Fig. 3:**
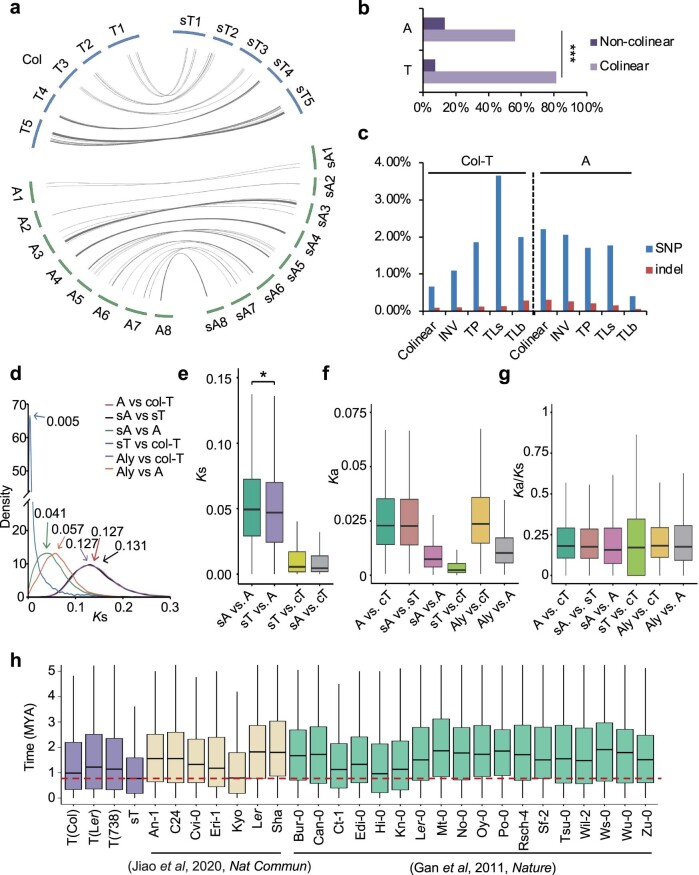
Genomic variation between *A. suecica*, *A. thaliana*, and *A. arenosa* genomes. **a**, Inversions between sT of Asu and Col-T (Ath) or between sA of Asu and A (Ara-related) of Allo738. **b**, Proportion of co-linear and non-co-linear regions between A and T. Three asterisks indicate statistical significance level of *P* < 0.001 (Fisher’s exact test). **c**, Proportion of SNP and indel distributions in sA and sT subgenomes of Asu relative to A and cT genomes. Non-co-linear: not collinear; INV: inversion; TP: transpositions; TLs: translocations within a subgenome; TLb: translocations between T and A subgenomes. **d**, Distribution of *K*s values for a set of 14,668 single-copy genes among A subgenome (Aar-related) of 738, cT (Ath, Col), sT and sA subgenomes of Asu, and *A. lyrata* (Aly). **e**, Distribution of *K*s values between genes in co-linear and TLb regions (co-linear regions: sA vs A, sT vs cT; TLb regions: sT vs A, sA vs cT). One asterisk indicates statistical significance level of *P* < 0.05 (Mann–Whitney U-test). **f**, Distribution of *K*a values between A and col-T (cT), *A. lyrata* (Aly), and sT and sA subgenomes of Asu. **g**, Distribution of *K*a/*K*s values as in (**f**). **h**, Boxplots of the estimated time (million years ago, MYA) for intact LTR insertions in 25 *A. thaliana* ecotypes. The red dashed line indicates the median of time in sT (ANOVA test). Source data

Two subgenomes in *A. suecica* have maintained high levels of colinearity and synteny compared to *A. lyrata* and extant progenitors (Aar and Ath, Col), respectively (Fig. 1b and Extended Data Fig. 1c,d). There were some large-scale sequence rearrangements, including a large translocation between sA1 and sT1 in Asu (Fig. 1c), which were confirmed by a Hi-C contact matrix analysis (Fig. 1d). Inversions and translocations occurred more frequently between *A. arenosa* and sA subgenome of *A. suecica* than between *A. thaliana* and sT subgenome (Fig. 1c and Extended Data Fig. 3a). This may suggest an increased rate of genetic diversity in outcrossing *A. arenosa* or a different *A. arenosa* strain involved in the formation of natural *A. suecica*. Whole-genome pairwise alignment analysis also showed more colinear regions in T (81.2%) than in A (56.2%) subgenome (*P* = 0, Fisher’s exact test) (Fig. 1e and Extended Data Fig. 3b). While indel distributions were similar among these structural variants, single-nucleotide polymorphism (SNP) frequency in Asu A/T subgenomic translocation regions was twofold higher in sT than in sA subgenome (Extended Data Fig. 3c), suggesting stable maintenance of high SNP frequency in the A segment and low SNP frequency in the T segment of these homologous exchange (HE) regions. Notably, the total amount of HEs between two subgenomes in Allo738 was relatively small, only ~21.5 kilobases (kb) of Ath origin in A subgenome and ~1.4 Mb of Aar origin in T subgenome (Supplementary Dataset 1), suggesting a minor role of HEs in evolution of *A. suecica* allotetraploid genomes.

To assess nucleotide sequence evolution, we estimated synonymous (*K*_s_) and non-synonymous (*K*_a_) mutation rates using 14,668 single-copy orthogroups identified in Ath, Aar, Asu and Aly (Extended Data Fig. 3d and Methods). *K*_s_ value distribution was higher between *A. arenosa* and sA subgenome than between *A. thaliana* and sT subgenome. However, *K*_s_ value was similar between *A. arenosa* and *A. thaliana* and between two *A. suecica* subgenomes, suggesting concerted and independent evolution of subgenomes in allotetraploids. Considering that large structural variation affects genomes of evolutionary rate^35^, genic sequences in rearranged regions between subgenomes, excluding small amounts of HEs, had lower neutral mutation rates than those in the syntenic regions (*P* < 0.05, Mann–Whitney U-test) (Extended Data Fig. 3e). Overall, *K*_a_/*K*_s_ values were uniformly small among those species tested (Extended Data Fig. 3f,g), implying purifying selection. However, purifying selection is generally weaker due to redundancy of homologues in allopolyploids as reported in *A. kamchatica*^26^ and *Capsella bursa*^36^, and allopolyploidy might have weakened natural selection because of this bottleneck effect.

Among repetitive DNA, proportion of TEs in each subgenome was relatively similar (20.9–23.8%), although A subgenome had twice as many as T subgenome (Table 1). The order of TE insertion time was *A. thaliana* > *A. lyrata* > *A. arenosa*. (Fig. 1f), which tended to correlate with different mating systems and reduced from the transition of outcrossing in *A. lyrata* to selfing^37^. However, long terminal repeat (LTR) retrotransposons were more active (younger insertion events) in sT subgenome of *A. suecica* than in *A. thaliana* Ler and Col. Among 25 other *A. thaliana* ecotypes published previously^38,39^, all except one had older LTR insertion events than sT. Kyo, an ecotype from Kyoto, Japan, had similar LTR insertion time to sT subgenome (Extended Data Fig. 3h). This result may suggest that T subgenome donor of Asu has more active LTRs.

### Gene family expansion and contraction in allotetraploids

**Fig. 2:**
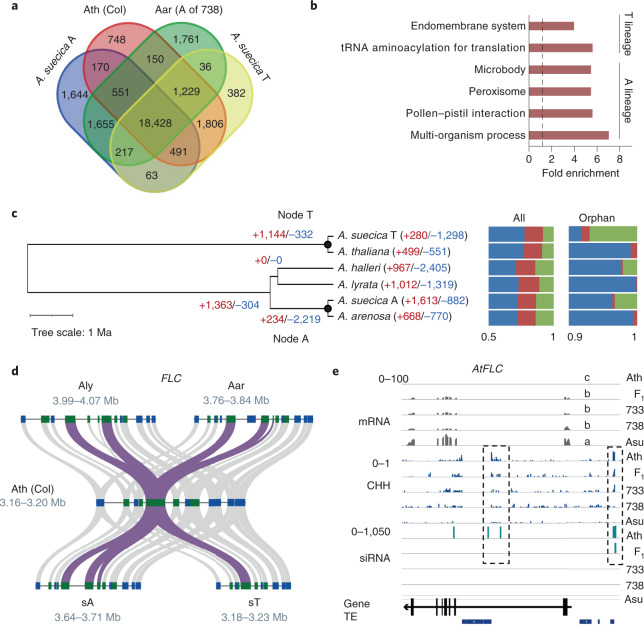
Gene family expansion and contraction. **a**, Venn diagram of orthologue clusters among *A. thaliana* (Ath, Col), *A. arenosa* (A subgenome of 738) and *A. suecica* (sT and sA subgenomes). **b**, GO enrichment terms of the genes specific to T lineage (Col, sT subgenome of *A. suecica*) and A lineage (*A. arenosa*, sA subgenome of *A. suecica*, *A. lyrata*, *A. halleri*). Dashed line indicates onefold enrichment. **c**, Expansion and contraction of gene families in *Arabidopsis-*related species with the numbers in parenthesis, indicating gene families subject to expansion (+red) and contraction (–blue), respectively. Black dots indicate node T (ancestor of *A. thaliana*) and node A (ancestor of *A. arenosa*), respectively. Bar graphs show the proportion of single-copy (blue), two-copy (red) and multi-copy (green) genes among all (All) and orphan (Orphan) genes in corresponding species. **d**, Micro-colinearity patterns between *FLC* and flanking genes in Col, Aar (A subgenome of 738), *A. suecica* (sT and sA subgenomes) and *A. lyrata* (Aly). Ribbons indicate colinearity of *FLC* genes (purple) and its flanking genes (grey). **e**, *FLC* expression is correlated with CHH methylation and siRNA levels in *A. thaliana* (Ath), F_1_, resynthesized allotetraploids (Allo733 and Allo738) and natural *A. suecica* (Asu). Scales indicate mRNA (0–100), CHH methylation density (0–1) and 24-nucleotide siRNA (0–1,050) levels. Different letters in mRNA (transcripts per kilobase million) indicate statistical significance of *P* < 0.05 (analysis of variance (ANOVA) test, *n* = 3). Source data

OrthoFinder identified 18,428 genes shared among *A. thaliana*, *A. arenosa* and A and T subgenomes of *A. suecica* (Fig. 2a). Among A-lineage orthogroups, gene families (744) from *A. arenosa*, *A. suecica*, *A. lyrata* and *A. halleri* were overrepresented in gene ontology (GO) terms of pollen–pistil interaction, multi-organism process, microbody and peroxisome (Fig. 2b), supporting their characteristics of outcrossing. GO enrichment terms of T-lineage orthogroups (1,415) from *A. thaliana* and *A. suecica* included endomembrane system and transfer RNA aminoacylation for translation.

**Extended Data Fig. 4:**
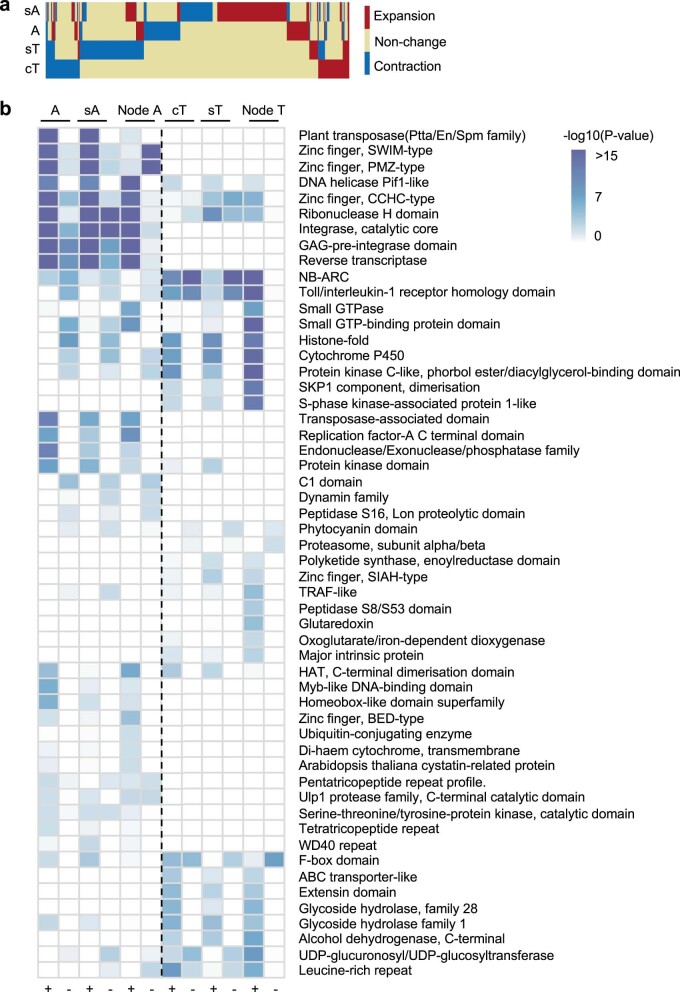
Expansion and contraction of gene families in A and T-related genomes. **a**, Heatmap showing expansion and contraction of gene families in A subgenome (Aar-related) of Allo738, Col-T (cT), and sA and sT subgenomes of *A. suecica*. **b**, Domain enrichment of gene family expansion/contraction (+/-) in A, sA, and their nearest ancestor (Node A in Fig. 2c), and cT, sT, and their nearest ancestor (Node T in Fig. 2c) (Fisher’s exact test). Source data

Analysis of gene family contraction and expansion revealed uneven rates of gain or loss among allotetraploid species examined (Fig. 2c). Unlike similar numbers of gene family gain or loss in its diploid relatives, there was more gene family loss in T subgenome (gain/loss; 280/1,298) and more gene family gain in A subgenome (1,613/882) of *A. suecica* (Fig. 2c), which were unique to subgenomes and their respective extant species, respectively (Extended Data Fig. 4a). Note that *A. lyrata* and *A. arenosa* may be more closely related^17^ and clustering between *A. lyrata* and *A. halleri* could result from the small number of species examined. Domain-based annotation showed a similar trend of gene family gain or loss between A- or T-lineage orthologues with a few exceptions (Extended Data Fig. 4b). For example, F-box and CCHC-type zinc finger domain gene families shrank in T lineage but expanded in A lineage and the trend was opposite for the gene families with histone-fold associated domains and cytochrome P450 domains (Extended Data Fig. 4b). These differences in the gene family loss or gain between subgenomes may suggest pervasive lineage-specific evolutionary heterogeneities in allopolyploids, as observed among five *Gossypium* allotetraploid species^14^.

### Flowering time variation and S locus evolution in allopolyploids

**Extended Data Fig. 5:**
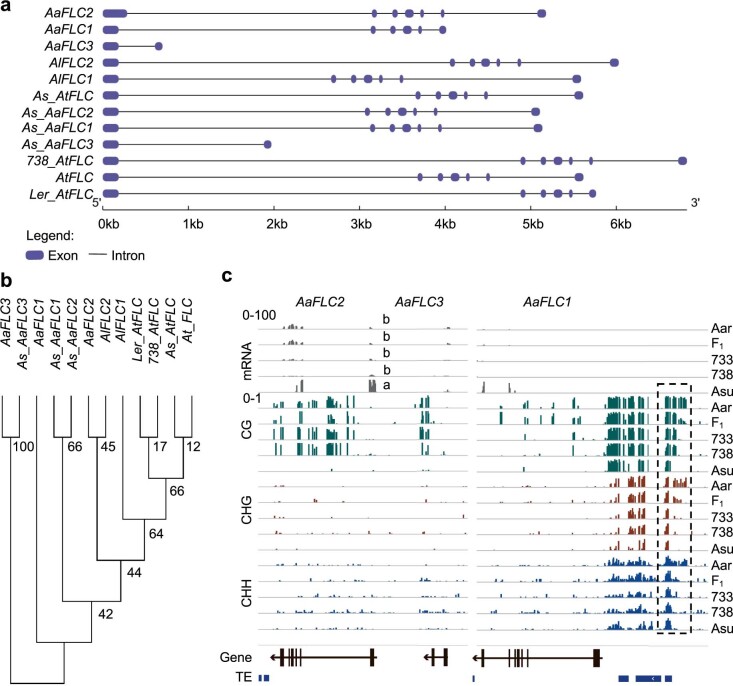
Comparative genomics of *FLC* loci in *A. suecica* and related species. **a**, Gene structure of *FLC* in different species: *A. thaliana* (Col*, AtFLC;* L*er_AtFLC)*, T subgenome of Allo738 (738_*AtFLC*), A subgenome (Aar) (*AaFLC1*, *AaFLC2*, *AaFLC3*), *A. suecica* (*As*_*AaFLC1*, *As_AaFLC2*, *As_AaFLC3*)*, A. lyrata* (*AlFLC1* and *AlFLC2*). **b**, Phylogenetic tree of *FLC* genes. **c**, CG, CHG and CHH methylation and mRNA expression patterns of *FLC* genes and their vicinity in A (Aar), F_1_, 733, 738 and *A. suecica* (Asu). The y axis scales shown above gBrowse tracks indicate mRNA (1–100) and methylation (0–1) levels. Differentially methylated regions are shown in a dashed box. Shown below are diagrams of three *FLC* loci (arrows indicating transcription direction) and TEs. Different letters in mRNA (TPM) indicate statistical significance of *P* < 0.05 (ANOVA test, *n* = 3). Source data

Copy number variation has functional consequences^40^. *FLOWERING LOCUS C* (*FLC*), a MADS-box transcription factor, inhibits early flowering^41^. *FLC* has a copy number variation among *Arabidopsis* species, one in *A. thaliana*, two in *A. lyrata* and three in *A. arenosa* and A subgenome of *A. suecica*^42^ (Fig. 2d). The first intron of *FLC* diverged dramatically (Extended Data Fig. 5a), for its role in *FLC* expression in response to vernalization via long non-coding RNAs^43,44^. Interestingly, *AaFLC1* and *AaFLC2* in *A. suecica* were clustered in one clade (Extended Data Fig. 5b), suggesting concerted evolution. The *FLC* copy number variation correlated with flowering time among these species^20^, earliest in *A. thaliana*, followed by *A. arenosa*, resynthesized allotetraploid F_1_ and stable Allo738 and Allo733 and the latest in natural *A. suecica*, which was consistent with higher *FLC* expression with lower DNA methylation levels in rosette leaves before bolting (Fig. 2e). Methylated regions are also target sites of small interfering RNAs^45^, which may induce RNA-directed DNA methylation (RdDM)^46^. Similar results were observed for other A-lineage *FLC* homologues in *A. arenosa* and *A. suecica* (Extended Data Fig. 5c). Thus, RdDM may also regulate *FLC* expression and vernalization.

Allopolyploids often become self-compatible, regardless of outcrossing behaviours in progenitors, suggesting silencing of self-incompatibility (S) locus from outcrossing *A. arenosa* in neo-allotetraploids and natural *A. suecica*^7,47,48^. S locus system comprises a combination of S locus cysteine-rich (SCR) protein in pollen coat and S locus receptor kinase (SRK) expressed on stigma surface^49^. *SRK* genes in A subgenome of resynthesized and natural *A. suecica* resembled *AhSRK1* and *AhSRK2* haplotypes^50^, respectively, both of which are weak alleles in the S locus dominance hierarchy than *AhSRK4* haplotype in T subgenome^51^ (Supplementary Fig. 2). These weak alleles that were immediately silenced by microRNA may contribute to a loss of self-incompatibility in early stages of allotetraploids and become non-functional in natural *A. suecica* (Supplementary Information).

### Dynamic changes of DNA methylation in allotetraploids

**Fig. 3:**
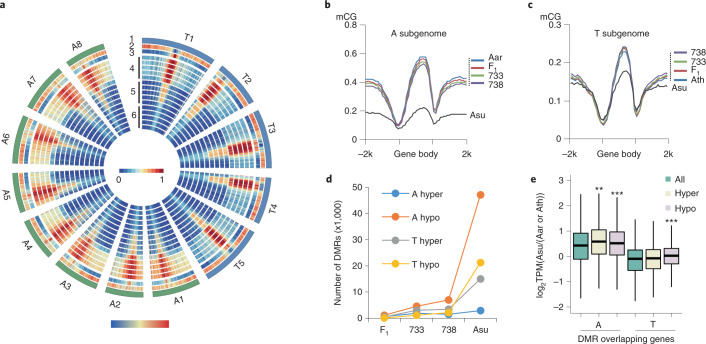
DNA methylation dynamics during the formation and evolution of allotetraploid *Arabidopsis*. **a**, Chromosome features and methylation distributions. Notes in circos plots: (1) chromosomes, (2) gene and (3) TE density and (4) CG, (5) CHG and (6) CHH methylation levels using 100-kb windows in Ath (Ler4) or Aar, F_1_, Allo733, Allo738 and *A. suecica* (in that order from outside to inside in each methylation context). Note that strain identity is omitted in naming T and A chromosomes. **b**,**c**, CG methylation levels in the gene body and flanking (2-kb) sequences of the A (**b**) and T (**c**) subgenomes in F_1_, Allo733 (733), Allo738 (738) and *A. suecica* (Asu), relative to *A. thaliana* (Ath) and *A. arenosa* (Aar), respectively. **d**, Numbers of DMRs between T subgenome and Ath (Col) or A subgenome and Aar in F_1_, 733, 738 and Asu, respectively. **e**, Expression ratio log_2_TPM(Asu/(Aar or Ath)) of the genes flanking a 2-kb region of hypo- or hyper-DMRs between *A. suecica* (Asu) and *A. arenosa* (Aar) or *A. thaliana* (Ath, Ler4). Three asterisks indicate a statistical significance level of *P* < 0.001 (Mann–Whitney U-test). TPM, transcripts per kilobase per million. Source data

**Extended Data Fig. 6:**
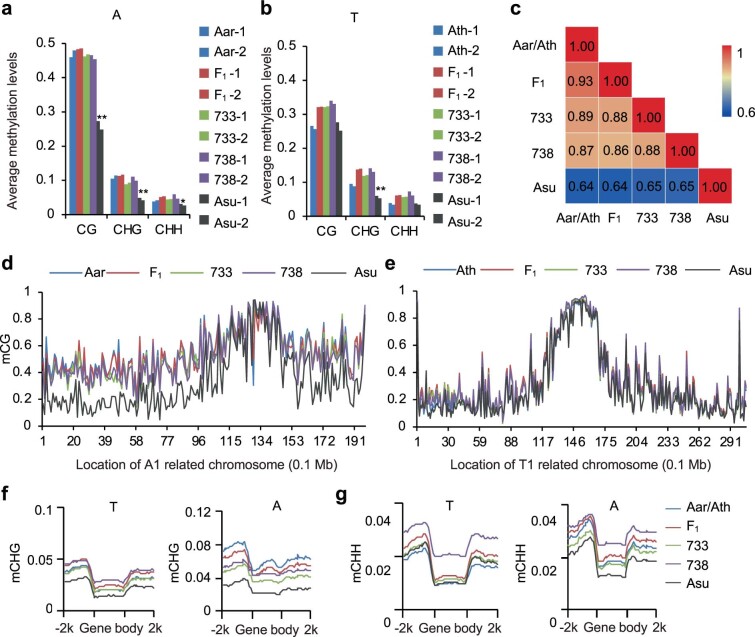
DNA methylation levels in F_1_, Allo733 (733), Allo738 (738) and *A. suecica* (Asu) allotetraploids and their related progenitors (Aar and Ath Ler4). **a**, **b**, Average methylation levels with two biological replicates in A (**a**) and T (**b**) related genomes or subgenomes in allotetraploids. Two asterisks indicate statistical significance level of *P* < 0.01 (Mann–Whitney U-test). **c**, Heatmap of pairwise comparisons between correlation coefficients of methylated cytosines in CG context of Aar, Ath L*er*4 (T), and respective subgenomes of F_1_, 733, 738 and Asu. **d**, **e**, CG methylation levels in A1 (**d**) or T1 (**e**) related chromosomes of *A. arenosa* (Aar), *A. thaliana* (Ath L*er*4), F_1_, Allo733 (733), Allo738 (738), and *A. suecica* (Asu). **f**, **g**, CHG and CHH methylation levels in genic regions of Ath L*er*4 (T) and Aar (A) relative to respective subgenomes in allotetraploids (F_1_, Allo733, Allo738, and Asu). Source data

Conserved genomic synteny between allotetraploids and related species may suggest a role for epigenetic modifications in non-additive gene expression in resynthesized and natural allopolyploids^7,22,52,53^. We examined methylome diversity in *A. thaliana* (Ath, Ler4), *A. arenosa* (Aar, 4*x*), F_1_, Allo738 and Allo733^11,12,27^ and natural *A. suecica* (Asu) (Extended Data Figs. 6a,b). To improve data comparability, we used shared methylation sites (35,853,727) and conserved cytosine with three or more reads among different lines for further analysis (Extended Data Fig. 6a,b). DNA methylation in plants occurs in CG, CHG and CHH (H = A, T or C) contexts^54^. Despite a similar proportion of repetitive DNA between *A. thaliana* and *A. arenosa* (Table 1), overall CG methylation levels were higher in *A. arenosa* than in *A. thaliana* (Fig. 3a and Extended Data Fig. 6a,b). Moreover, average methylation levels were highly correlated between parents (Aar/Ath) and F_1_, Allo733, Allo738 or *A. suecica* (from the highest to the lowest) (Extended Data Fig. 6c). However, in *A. suecica*, A subgenome had lower methylation levels in all contexts especially the CG sites than *A. arenosa* (*P* < 0.01, Mann–Whitney U-test), while methylation levels in CHG sites were lower in T subgenome (*P* < 0.01, Mann–Whitney U-test) than in *A. thaliana* (Fig. 3a and Extended Data Fig. 6a,b,d,e). This CG hypomethylation between Asu and F_1_, Allo733 or Allo738 was observed in all allotetraploids and more profound in A subgenome with a sharp reduction of methylation levels in the gene body and 5′ and 3′ sequences (*P* < 0.001, Asu versus Aar, Mann–Whitney U-test) (Fig. 3b), whereas in T subgenome hypomethylation might occur mainly in the gene body (*P* > 0.05, Asu versus Ath, Mann–Whitney U-test) (Fig. 3c). A similar trend was also observed in CHG methylation levels (Extended Data Fig. 6f) and to a lesser degree in CHH context (Extended Data Figs. 6g) of A subgenome. The data suggest that epigenomic modifications are dynamic, which occur largely in CG and CHG sites of natural *A. suecica* and throughout coding sequences, including 5′ and 3′ untranslated regions (UTRs) of A subgenome and in the gene body of T subgenome.

**Extended Data Fig. 7:**
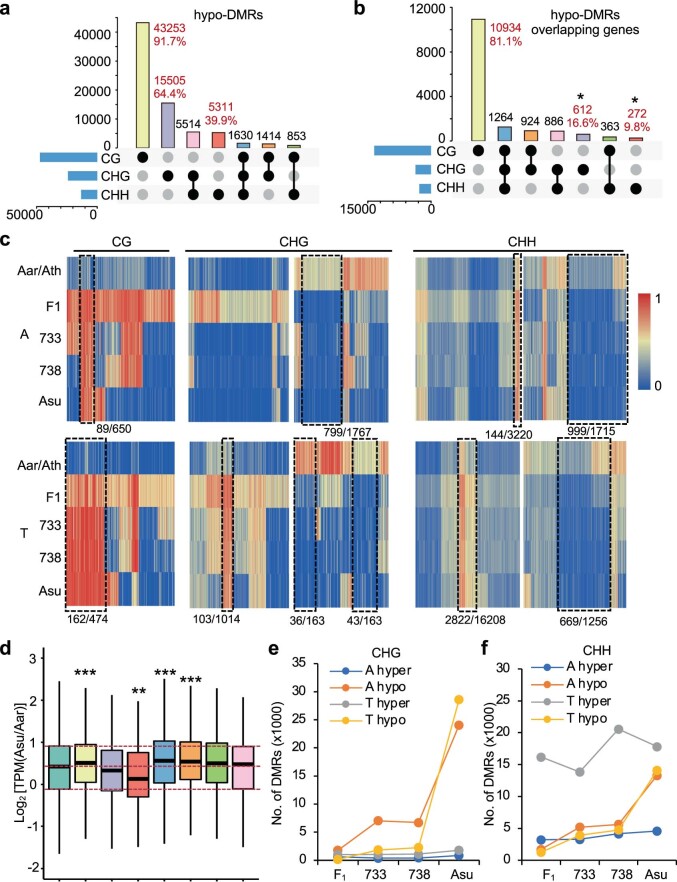
Association of gene expression with hypo-DMRs between sA subgenome of *A. suecica* and *A. arenosa* (Aar, A) and between sT subgenome and *A. thaliana* L*er*4 (Ath, T). **a**, Upset diagram of hypo-DMRs in CG, CHG and CHH context between sA and A. Numbers and percentages specific to each context were shown in red. **b**, Upset diagram of hypo-DMR-overlapping genes in CG, CHG and CHH context between sA and A. An asterisk indicates the fraction of the unique CHG or CHH DMRs (unique number / total number of DMRs) compared to that of their unique overlapping genes (unique number / total number of associated genes) was significantly reduced (*P* < 0.05, Fisher’s exact test). **c**, Heatmap of CG, CHG, and CHH DMRs (sT-T or sA-A) in Aar/Ath, F_1_, 733, 738, and Asu. Boxed regions show maintenance of DMRs from F1 to Allo733, Allo738, and *A. suecica*. **d**, Expression ratio (Log2[TPM(Asu/Aar)] of the genes as shown in (**b**). Colours (from left to right) indicate all genes (pale blue) and the genes flanked (2 kb) with hypo-DMRs in CG only (yellow), CHG only (purple), CHH only (red), all three (dark blue), both CG and CHG (orange), both CHG and CHH (green), and both CHH and CG (pink). One and three asterisks indicate statistical significance levels of *P* < 0.05 and P < 0.001, respectively (Mann–Whitney U-test). **e**, **f**, Number of CHG (**e**) and CHH (**f**) differentially methylated regions (DMRs) (sT-T or sA-A) in F_1_, 733, 738, and Asu, respectively. Source data

To track methylation changes during polyploid formation and evolution, we analysed differentially methylated regions (DMRs) between T subgenome and *A. thaliana* (Ath) or A subgenome and *A. arenosa* (Aar) in each allotetraploid. The majority of two DMR groups did not overlap (Extended Data Fig. 7a). Among 13,485 CG, 3,686 CHG and 2,785 CHH hypo-DMRs that overlapped with genes (within a 2-kb flanking region), 10,934 (81.8%), 612 (16.6%) and 272 (9.8%) were specific to CG, CHG and CHH DMRs, respectively (Extended Data Fig. 7b), suggesting association of most CG DMRs with genes. Some (14–62% in A subgenome and 14–44% in T subgenome) of these DMRs induced in F_1_ were maintained in resynthesized and natural *A. suecica* (Extended Data Figs. 7c), as observed in cotton allotetraploids^53^. Notably, hypo-DMRs in CG context were negatively associated with expression levels of DMR-associated genes in natural *A. suecica* (Extended Data Fig. 7d). Relative to DMRs between parents (Aar and Ath), the number of hypo-DMRs in A subgenome was substantially higher than that of hyper-DMRs in *A. suecica*, but hypo- and hyper-DMRs in T subgenome were similar and increased to a middle level in Asu (Fig. 3d). Moreover, expression levels of DMR-associated genes correlated negatively with hypo-DMRs but not with hyper-DMRs in both A and T subgenomes (Fig. 3e).

The number of CHG and CHH hypo-DMRs had similar changes in A subgenome, which increased slightly in F_1_ and neo-allotetraploids and dramatically in *A. suecica*, while CHG hypo-DMRs in T subgenome increased dramatically only in *A. suecica* (Extended Data Fig. 7e,f). CHH hypo-DMRs displayed a similar trend to CHG hypo-DMRs, except that CHH hyper-DMRs had the highest number in T subgenome among all allotetraploids (Extended Data Fig. 7f). Considering that CG methylation is relatively abundant and stable and correlates with gene expression, we focused most analyses on CG methylation dynamics.

**Extended Data Fig. 8:**
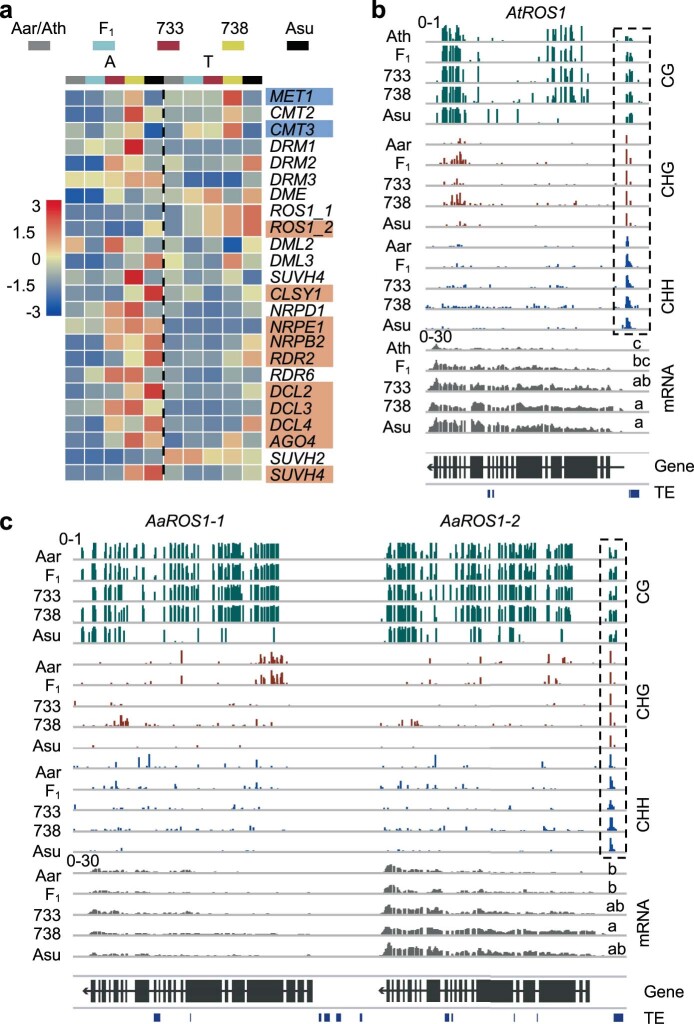
Differential expression of methylation pathway genes including *AtROS1*, *AaROS1–1* and *AaROS1–2* in allotetraploids. **a**, Heatmap of transcript levels (TPM: transcripts per kilobase per million) of methylation pathway genes in A and T subgenomes of Arabidopsis allotetraploids, respectively. From parents (Aar/Ath) to *A. suecica* (Asu), the upregulated genes was marked orange, while down-regulated genes was marked blue. **b**, **c**, gBrowse tracks showing CG (green), CHG (red), and CHH (blue) methylation and mRNA expression (grey) levels in *AtROS1* (**b**) and *AaROS1–1* and *AaROS1–2* (**c**) genes and their vicinity in *A. thaliana* (Ath) and *A. arenosa* (Aar), F_1_, 733, 738 and *A. suecica* (Asu). The regions associated with a TE in the 5′ sequence are shown in a dashed box. The y axis scales shown above the gBrowse tracks indicate mRNA (0–30) and methylation (0–1) levels. Different letters in mRNA (TPM) indicate statistical significance of *P* < 0.05 (ANOVA test, *n* = 3). Source data

In plants, CG and CHG methylation is largely maintained by methyltransferase 1 (MET1)^55^ and chromo methyltransferase 3 (CMT3)^56^, respectively. CHH methylation is controlled by RdDM or RdDM-independent pathway^46^. *Repressor of Silencing1* (*ROS1*), encoding DNA glycosylase/AP lyase^57^, is responsible for demethylation and maintains methylation homoeostasis by RdDM^58^. Consistent with genome-wide hypomethylation in *A. suecica*, *MET1* and *CMT3* were expressed at the lowest level in *A. suecica* and slightly higher levels in F_1_ and two neo-allotetraploids, whereas *AtROS1-1* and *AaROS1–2* were expressed at high levels in *A. suecica* (Extended Data Fig. 8a). Upregulation of *ROS1* correlated with increased CHH methylation levels in promoter region (within a TE) and decreased CG methylation levels in gene body of *AtROS1* (Extended Data Fig. 8b) and *AaROS1–2* (Extended Data Fig. 8c) in neo-allotetraploids and *A. suecica*. *AaROS1–1* expression level was low in all lines tested. This type of allelic expression variation was also observed for *CCA1* and *FLC* homologous loci in allotetraploids^20,59^, which may be controlled by a mechanism similar to nucleolar dominance^19,60^. Consistent with feedback regulation of *ROS1* expression by RdDM pathway^58^, expression of several RdDM pathway genes examined was upregulated in *A. suecica* and neo-allotetraploids (Extended Data Fig. 8a), while CHH methylation levels were higher in the F_1_, Allo733 and Allo738 than in *A. thaliana* or *A. arenosa* (Extended Data Fig. 6a,b and Extended Data Fig. 8b). We speculate that increased CHH methylation via RdDM pathway may lead to upregulation of *ROS1* expression, reducing overall methylation levels of A subgenome in natural *A. suecica*.

### Homologous convergence of methylation changes in allotetraploids

**Fig. 4:**
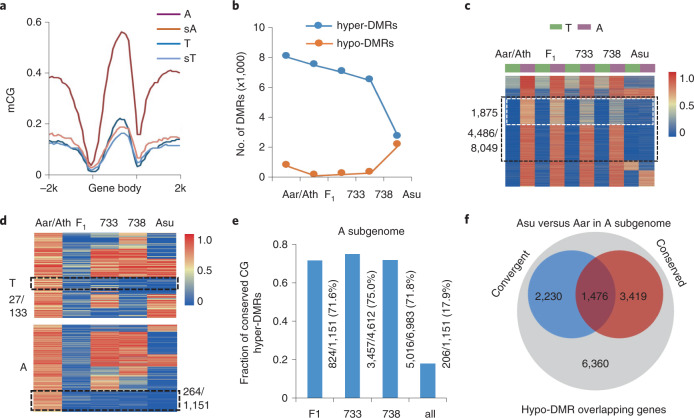
Convergence and inheritance of CG methylation levels between two subgenomes in allotetraploids. **a**, CG methylation levels of homologues in *A. thaliana* (Ler4, T), *A. arenosa* (Aar, A), sT and sA subgenomes of *A. suecica*. **b**, Numbers of DMRs between T subgenome and Ath (Col) or A subgenome and Aar in F_1_, 733, 738 and Asu, respectively. Note that Allo733 and Allo738 may be treated as biological replicates of resynthesized allotetraploids. **c**, Clustering analysis of CG hyper-DMRs (A-T) in Aar/Ath and their changes in F_1_, Allo733 (733), Allo738 (738) and *A. suecica* (Asu), respectively. Dashed black box indicates 4,486 convergent DMRs where hyper-DMRs between A and T (Ler4) were conserved in newly formed allotetraploids and reduced to the sT subgenome level in Asu. Note that the upper portion (white dashed box) indicates the overlap group (1,875) with conserved DMRs (also see **e**). **d**, Clustering of CG hypo-DMRs in F_1_ and their changes in 733, 738 and Asu relative to Aar/Ath. Black dashed boxes indicate hypo-DMRs between T subgenome and Ath (upper panel) and between A subgenome and Aar (lower panel) in F_1_ were conserved in Allo733, Allo738 and Asu. **e**, Fraction of conserved CG hypo-DMRs in F_1_, Allo733, Allo738 and all three lines and their inheritance in Asu relative to Aar, with the numbers (conserved/total) shown to the left of each column. **f**, Venn diagram of the genes that overlapped with convergent (blue) and conserved (red) CG hypo-DMRs in Asu relative to Aar. Absolute values of CG methylation change thresholds were 0.5 in **c**,**d**. Source data

Changes in DNA methylation levels between *A. arenosa* and A subgenome of allotetraploids can become convergent or conserved. Conserved DMRs were defined as hypo-DMRs in Asu and consistently present in F_1_, Allo733 or Allo738, while convergent DMRs were identified as hyper-DMRs between Aar and Ath and in F_1_ and neo-allotetraploids and decreased to a similar level to T subgenome in Asu. We examined CG methylation levels of homologous gene pairs between two subgenomes in *A. suecica* and between Ath Ler4 (T) and Aar (A). In contrast to substantially overall higher methylation levels in *A. arenosa* than in *A. thaliana*, *A. suecica* had similar methylation levels between A and T homologues (Fig. 4a). This was accompanied by dramatic reduction of CG methylation levels in A homologues, which convergently reached a similar level to T homologues in *A. suecica*. Two subgenomes tend to maintain similar methylation levels during allopolyploid evolution.

We further analysed dynamics of hypo- and hyper-DMRs between Aar (A) and Ath (T) and between two subgenomes among different allotetraploids (Fig. 4b). The number of hyper-DMRs was reduced slightly from F_1_ to Allo733 and Allo738 (~F_10_) and dramatically in natural *A. suecica*, while the number of hypo-DMRs was relatively similar among F_1_ and neo-allotetraploids but increased in *A. suecica*. Remarkably, 55.7% (4,486/8,049) of these hyper-DMRs (A versus T) were conserved in F_1_, Allo733 and Allo738 and became hypomethylated at the same level in *A. suecica* (Fig. 4c), while smaller fractions of DMRs that remained hypermethylated in the A subgenome became hypermethylated in T subgenome or both. In cotton, it is the low methylated subgenome that has hypermethylated to reach a similar level in allotetraploids^53^. Although the mode of changes is different between *Arabidopsis* and cotton allotetraploids, most DMRs between two subgenomes reach similar methylation levels and evolve convergently during allotetraploid evolution.

In addition to convergent changes in DMRs, subsets of hypo-DMRs induced in the F_1_ were maintained after ten or more generations in Allo733 and Allo738, some of which were also conserved in *A. suecica* (Fig. 4d). The overlap between convergent and conserved groups represented those DMRs convergent in neo-allotetraploids and maintained in Asu (Fig. 4c). Although methylated DMRs in CG, CHG and CHH contexts could be inherited across generations, more hypo-DMRs were inherited than hyper-DMRs (Fig. 4d,e and Extended Data Fig. 7c), consistent with global demethylation of the A subgenome. For example, CG hypo-DMRs in A subgenome of *A. suecica* overlapped ~71.6% (824/1,151) in F_1_, ~75.0% in Allo733 and 71.8% in Allo738 (Fig. 4e). Among 13,485 genes that overlapped with CG hypo-DMRs in Asu A subgenome, 3,706 (27.5%) were convergent (*P* < 8.01 × 10^–6^) and 4,895 (36.3%) were conserved (*P* < 0.29), of which 1,476 (11.0%) overlapped (*P* = 1, all with Fisher’s exact test) (Fig. 4f).

### DNA methylation and expression correlation of reproduction-associated genes in *A. suecica*

**Extended Data Fig. 9:**
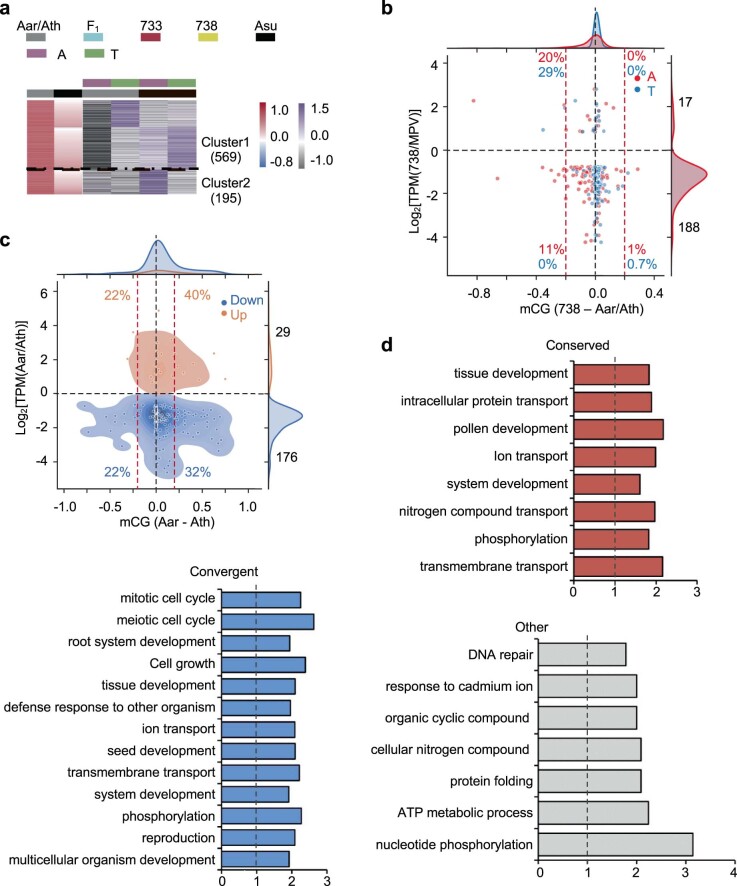
Gene ontology (GO) enrichment terms for hypo-DMR-associated genes and association of CG methylation with non-additive gene expression in allotetraploids. **a**, The first two columns show CG DMR (A-T) levels (difference threshold > 0.5) of homologous genes in *A. thaliana* (*Ler*4, T) and *A. arenosa* (Aar/Ath) and in *A. suecica* (Asu). The remaining four columns indicate expression levels (TPM) of these genes in Aar/Ath (L*er*4 and Aar) and Asu, respectively. **b**, Correlation between expression fold changes of shared differentially expressed genes (DEGs) between Aar and Ath (Wang *et al*., 2016) (y axis) and methylation differences (x axis). **c**, Correlation between expression fold changes of shared DEGs between Allo738 and MPV (Wang *et al*., 2016) (y axis) and methylation differences between A (red) and T (blue) subgenomes in Allo738 against Aar/Ath (x axis). The red dashed line indicates 0.2 value of methylation difference; the percentage statistics indicates the fraction of genes with more than 0.2 methylation differences in each quadrant. The genes used in (**b**) and (**c**) were the shared DEGs of Aar vs. L*er*4 and Allo738 vs. MPV. **d**, GO term overrepresentation for the genes showing conserved (red), convergent (blue), and other (grey) CG hypo-DMRs between sA of *A. suecica* and *A. arenosa* (Aar, A). Dashed line indicates onefold enrichment. Source data

These methylation changes affect homologue expression in *A. suecica*. Among 764 genes that were differentially methylated between Aar and Ath but have similar homologue methylation levels in *A. suecica*, 74.5% (569/764) showed decreased expression difference in two subgenomes of *A. suecica* relative to their parents (Extended Data Fig. 9a), suggesting that methylation may contribute to concerted expression level between homologues. This result may explain genome-wide non-additive gene expression in *A. suecica* as observed using microarrays^11^. However, the microarray data did not correlate well with allelic DNA methylation patterns (Extended Data Fig. 9b, c), probably because allelic expression cannot be discriminated in microarray experiments. Alternatively, DNA methylation might not explain non-additive gene expression in early generations of allotetraploids; other modifications such as histone H3K27me3 may be involved, as observed in an interspecific hybrid^61^. Over time, convergent and concerted methylation changes between subgenomes may contribute to gene expression variation and stability in *A. suecica*.

To test consequences of convergent and conserved DMRs in *A. suecica*, we analysed GO enrichments of hypo-DMR-associated genes in natural *A. suecica*. Convergent CG hypo-DMR-associated genes were overrepresented in reproduction, seed development, system development and cell cycle, whereas the conserved hypo-DMR-associated genes were involved in transmembrane transport, pollen development and protein phosphorylation (Extended Data Fig. 9d). Those genes involved in several distinct pathways may suggest roles of DNA methylation in shaping plant growth, development and response to stresses and genome stability in allopolyploids.

**Fig. 5:**
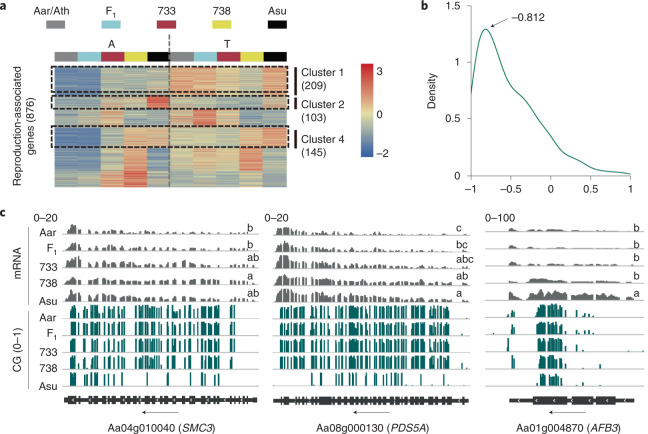
Association of CG methylation with expression of reproduction-related genes in *Arabidopsis* allotetraploids. **a**, Clustering analysis of expression levels of reproduction-related genes (GO:0000003) in *A. arenosa* (Aar, A) and *A.thaliana* (Ler4, T) (Aar/Ath), F_1_, Allo733 (733), Allo738 (738) and *A. suecica* (Asu). **b**, Density plot of correlation coefficients between expression and CG methylation levels of the reproduction-related genes from clusters 1, 2 and 4 in the A subgenome. **c**, CG methylation near genic regions of *SMC3*, *PSD5A* and *AFB3* and their mRNA expression patterns in Aar, F_1_, Allo733 (733), Allo738 (738) and Asu. Black arrows indicate the orientation of genes. SMC3, STRUCTURAL MAINTENANCE OF CHROMOSOMES3; PDS5A, PHYTOENE DESATURASE; AFB3, AUXIN SIGNALING F-BOX3. Scales indicate mRNA (0–20 and 0–100) and CG methylation density (0–1) levels. Different letters in mRNA (TPM) indicate statistical significance of *P* < 0.05 (ANOVA test, *n* = 3). Source data

**Extended Data Fig. 10:**
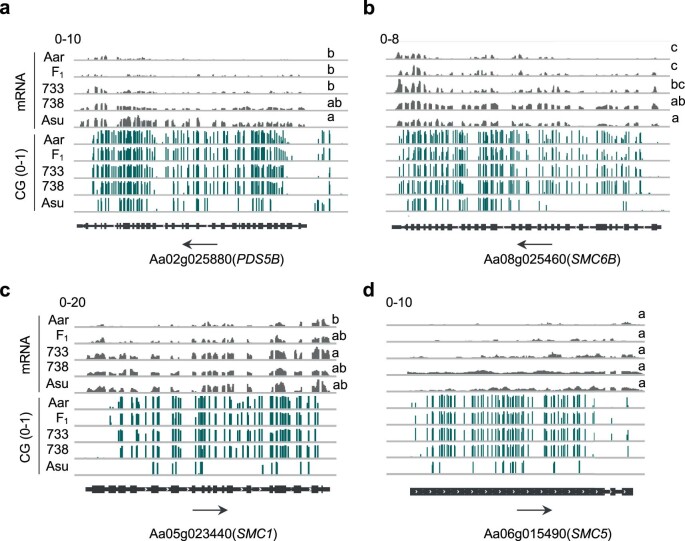
Association of CG methylation with expression of reproduction-related genes in *Arabidopsis* allotetraploids. **a-d**, CG methylation near genic regions of *PSD5B* (**a**), *SMC6B* (**b**), *SMC1* (**c**), and *SMC5* (**d**) and their mRNA expression patterns in *A. arenosa* (Aar), F_1_, Allo733 (733), Allo738 (738), and natural *A. suecica* (Asu). Black arrow indicate the orientation of gene. PDS5B: One of 5 PO76/PDS5 cohesion cofactor orthologs of *Arabidopsis*; SMC: STRUCTURAL MAINTENANCE OF CHROMOSOMES. Scales indicate mRNA (0–10 and 0–8) and CG methylation density (0–1) levels. Different letters in mRNA (TPM) indicate statistical significance of *P* < 0.05 (ANOVA test, *n* = 3). Source data

Interestingly, GO term of reproduction (GO:0000003) was overrepresented for convergent DMR-associated genes (Extended Data Fig. 9d) and 52.2% (457/876) of reproduction-related genes were upregulated in *A. suecica* (Fig. 5a), including upregulation of 209 A and 248 both homologues. Expression levels of these three gene clusters correlated negatively with CG methylation levels (Fig. 5b). For example, *STRUCTURAL MAINTENANCE OF CHROMOSOMES3* (*SMC3*) is an essential gene for sister chromatid alignment and plant viability^62,63^. *PHYTOENE DESATURASE5A* (*PDS5A*) regulates mitotic sister chromatid cohesion^64^ and *AUXIN SIGNALING F-BOX3 (AFB3)* is associated with pollen maturation and stamen development^65^. CG methylation levels of three genes (*SMC1*, *SMC6B* and *PDS5B*) in the same family of *SMC3* and *PDS5A* were reduced from Allo733 and Allo738 to *A. suecica* and their expression was upregulated in *A. suecica*, compared to that in Ath and F_1_ (Fig. 5c and Extended Data Fig. 10a–d). Notably, downregulation of *PDS5* (Traes_7DS_0DA047A5F), a homologue of *PDS5A* and *SMC6B* (Traes_5DL_67A6B8CEB), a homologue of *SMC3*, in allohexaploid wheat led to meiotic instability^66^. Moreover, some of these genes, including *PDS5B* and *SMC3,* are highly diverged and under strong selection in *A. arenosa* tetraploids^64^. Meiotic instability is often associated with newly formed allotetraploids (F_1_) and is gradually improved in resynthesized allotetraploids by self-pollination^52,67^. We predict that demethylation and upregulation of A homologues of reproduction-related genes may contribute to reproductive stability during evolution of *A. suecica* allotetraploids.

## Discussion

In this study, we generated high-quality sequences of *A. suecica* natural and neo-allotetraploids including progenitors and interrogated genomic and epigenomic contributions to polyploid formation and evolution. *A. suecica* allotetraploids have maintained genomic synteny and gene content, which is another example of stable allopolyploids, following cotton allotetraploids^14^. The genomic stability is associated with subtle genomic, TE and gene family changes, including copy number and SNP variation in the genes related to flowering time and other adaptive traits. For example, *The Boy Named Sue* (*BYS*) is a fertility quantitative trait locus (QTL)^67^ and spans ~240 kb on A4 chromosome, consisting of 56 annotated genes including a *FIS2* homologue^68^. *FIS2* is absent in *A. lyrata* and has variable sequences in *A. arenosa* and *A. suecica* (Supplementary Fig. 3). Function of candidate genes for the *BYS* locus remains to be investigated.

Newly formed allotetraploids have low fertility^12^ due to self-incompatibility locus^7,47^ (Supplementary Fig. 2), as well as meiotic instability^67^. Hypomethylation of the A subgenome may lead to upregulation of many genes involved in reproduction (meiosis, mitosis and pollination) and adaptation (stress responses), which can improve fertility and stability in allotetraploids. In wheat, downregulation of meiosis-related genes such as *PDS5* and *SMC6* is sufficient to confer unstable meiotic phenotypes^66^. Hypermethylation of reproduction-related genes may lead to gene loss, as some essential genes including meiotic genes can rapidly return to single copy following genome duplication^69,70^. For example, *ASY2* (asynaptic mutant2), a homologue of *ASY1* (ref. ^71^), is heavily methylated and poorly expressed in Allo733, Allo738 and *A. suecica* and possesses a frameshift mutation, which are not observed in *A. thaliana* or *A. arenosa* (Supplementary Fig. 4). More transcriptome, methylome and resequencing data of *A. arenosa* and *A. suecica* populations in specific developmental stages such as meiosis are needed to elucidate this relationship between hypermethylation and retention of duplicate genes in allopolyploids.

Remarkably, balanced genomic diversifications in allotetraploids are accompanied by convergent and concerted changes in DNA methylation between two subgenomes. On one hand, DNA methylation of the A subgenome is reduced immediately in F_1_, gradually during selfing in allotetraploids and convergently to the T-subgenome level in natural *A. suecica*. In cotton allotetraploids, it was the low methylated subgenome that became highly methylated to reach a similar level in the allotetraploids^53^, resulting in convergent methylation levels of the two subgenomes. On the other hand, subsets of differentially methylated regions are conserved from F_1_ to resynthesized allotetraploids and natural *A. suecica*, as observed in cotton allotetraploids^53^. These dual processes of convergent and conserved epigenomic modifications may provide a basis for allotetraploids to stabilize the two subgenomes derived from divergent hybridizing species. A combination of balanced genomic diversity and pervasive epigenomic modifications may be responsible for stabilizing subgenomes in cotton allotetraploids, which were formed ~1.5 million years ago^14,53^, as well as in resynthesized hexaploid wheat^72^ and tetraploid *A. suecica*. An obvious question is why other plant polyploids, including newly formed *B. napus*^9,73^, *T. miscellus*^10^ and resynthesized tetraploid wheat^74^, display rapid genomic reshuffling. One possibility is that new species form at the right time and under suitable conditions. The species or strains used to form *B. napus* or wheat 8,000–10,000 years ago^75^ may become extinct. Alternatively, homologous chromosomes from closely related progenitors may pair as in *Tragopogon*^10^. In *A. suecica*, sT and sA subgenomes are divergent enough to prevent homologous exchanges and subject to convergent and concerted changes in DNA methylation and gene expression including silencing of uniparental ribosomal DNA (rDNA) loci epigenetically via nucleolar dominance^19,60^. With advanced sequencing and epigenomic technologies, this paradox of rapid genomic reshuffling and genomic stability will be addressed to illuminate our understanding of polyploid genome evolution and to empower our efforts on editing genes and modifying epigenetic landscapes for crop improvement.

## Methods

### Plant materials

Plant materials included *A. thaliana* autotetraploid (Ler4, CS3900), *A. arenosa* (Care-1, CS3901), F_1_ resynthesized allotetraploids, two F_10_ synthetic allotetraploids with verified chromosome compositions (Allo733, Allo738)^27^ and a natural allotetraploid of *A. suecica* (As9502). All plants were grown in the growth chamber under the 16 h light/ 8 h dark cycle at 20 °C.

### Genome sequencing and assembly

DNA was extracted from young leaves of Allo738 and *A. suecica* and sequenced on the PacBio Sequel platform using 11 and eight SMRT cells to produce 37.02 gigabases (Gb) (136X genome equivalent) and 35.52 Gb (132X) of raw data, respectively. The PacBio long clean reads were corrected and assembled to contigs by MECAT (v.1.0) with parameters (correctedErrorRate 0.02)^76^. Next, the clean subreads were mapped to the assembled contigs using BLASR of SMRTLINK and errors were corrected by ARROW of SMRTLINK (v.5.0.1.9585)^77^. The Illumina pair-end reads (~80X) were mapped to consensus contigs by BWA (v.0.7.15-r1140)^78^ and further polished by Pillon (v.1.22) with the following parameters (--fix bases --changes --diploid)^79^. For Allo738 and *A. suecica*, chromatin conformation capture (3C or Hi-C) sequencing data consisting of 80–90 millions of effective read pairs were mapped to final contigs by Juicer (v.1.6.2)^80^ with default parameters and scaffolded to the chromosome-scale assembly by a three-dimensional de novo DNA assembly (3D DNA) pipeline (v.180114) with parameters (-r 3 -m diploid)^81^. Finally, we manually modified the assembly error using Juicebox (v.1.8.8)^82^ and generated the ultimate scaffolds, whose largest 13 scaffolds represented 13 chromosomes. The A subgenome of Allo738 represents the genome of *A. arenosa* (CS3901) and the T subgenome represents the genome of the autotetraploid *A. thaliana* (Ler), as Allo738 was generated by pollinating autotetraploid *A. thaliana* with tetraploid *A. arenosa* and self-pollinated for more than ten generations to minimize heterozygosity^11,27^.

### Repeat identification

Repeats were de novo annotated and classified as repeat consensus database for Allo738 and *A. suecica* assemblies using RepeatModeler (v.1.0.11) (http://www.repeatmasker.org/). The *Arabidopsis* section of Repbase (v.20181026) and RepeatPeps (v.20181026) (https://www.girinst.org/) and MIPS (mipsREdat_9.3p)^83^ repeat databases were used to correct de novo repeat database by BLASTN (v.2.5.0+) with criteria of more than 80% identity, 50% coverage and 80-base pair (bp) length^84^. The corrected repeat database with more than 80% identity and 50% coverage of protein-coding genes (without TE-associated genes) of *Arabidopsis* was removed. We then combined the corrected de novo database with the *Arabidopsis* section of Repbase and whole-genome repeat sequences of TAIR10 (https://www.arabidopsis.org/) to generate a final repeat database. In addition, intact LTR retrotransposons were de novo annotated using LTR-FINDER (v.1.0.7) with parameters (-D 20000 -d 1000 -L 3500 -l 100 -p 20 -C -M 0.9)^85^ and LTR_retriever (v.2.0) with parameters (-similar 90 -vic 10 -seed 20 -seqids yes -minlenltr 100 -maxlenltr 7000 -mintsd 4 -maxtsd 6 -motif TGCA -motifmis 1)^86^. Lastly, repeats were identified from the intact LTR-masked assembly by RepeatMasker (v.4.0.7) with parameters (-cutoff 250) (http://www.repeatmasker.org/) against the final repeat database. To estimate the insertion time of LTR, we used the Jukes–Cantor model to calculate the distance *K* (ref. ^87^). Then the insertion time *t* was calculated as *t* = *K*/2*r*, where *r* is the rate of nucleotide substitution, which was 7 × 10^−9^ per site per generation (assumed to equal one year) by LTR_retriever.

### Gene annotation

Genes were annotated by the integration of ab initio prediction, homology-based prediction and RNA sequencing (RNA-seq) data evidence for Allo738 and *A. suecica*. RNA-seq reads from different tissues were mapped to the assembly using HISAT2 (v.2.1.0)^88^ to generate transcripts by StringTie (v.1.3.3b)^89^. Simultaneously, the genome-guided pipeline of Trinity (v.2.6.6) with parameters (-I 20000)^90^ based on GSNAP (v.2018-07-04)^91^ software was used to assemble transcripts which then aligned to the assembly by PASApipeline (v.2.3.3) with parameters (--ALIGNERS blat,gmap --MAX_INTRON_LENGTH 20000 --transcribed_is_aligned_orient --stringent_alignment_overlap 30.0)^92^. Next, we used TransDecoder (v.5.3.0) to identify candidate coding regions within transcript sequences generated by both Trinity and StringTie. AUGUSTUS (v.3.2.2)^93^ was used for ab initio gene prediction on the basis of the hints of intron–exon boundaries from bam files of HISAT2 and repeat boundaries from RepeatMasker and model training was based on the transcripts assembled from Trinity. The homology-based prediction was conducted via Exonerate (v.2.2.0) with parameters (--percent 50 --maxintron 20000 -n 1)^94^ on the basis of *Arabidopsis* protein sequences against the assembly. EVidenceModeler (v.1.1.1) with parameters (--segment size 500000 --overlapSize 10000)^95^ was used to integrate the gene annotation files generated by these three methods with different weights: 1 for Augustus, 14 for Exonerate, 5 for PASA and 14 for TransDecoder. Finally, UTRs and alternatively spliced models were added by PASApipeline.

Genes were characterized for their putative function by performing InterProScan (v.5.32-71.0) with parameters (-appl ProDom, SMART,TIGRFAM, Pfam and SUPERFAMILY,PrositeProfiles -goterms -pa -iprlookup)^96^. Small RNAs were inferred by Infernal (v.1.1.2)^97^ against the Rfam database (release 14.1)^98^ and tRNAs were annotated by tRNAscan-SE (v.2.0)^99^.

### Assessment of assembly accuracy and integrity

We evaluated the integrity of the assembly by BUSCO (v.3.0.2)^29^ and the accuracy of the assembly through whole-genome alignment against the reference genome of *A. thaliana* (TAIR10, Ler)^33^ or *A. lyrata* (Alyrata_384_v2.1 from JGI)^100^ by MUMmer (v.4.0.0beta2) with parameters (--mum -l 100 -c 1000 -d 10 --banded -D 5 && delta-filter -i 95 -o 95)^101^, which identified one-to-one and multiple-to-multiple (M-to-M, including duplicates) alignment regions. Dotplots were constructed using mummerplot in MUMmer. For analysis of Allo738 genome stability, whole-genome alignments were performed between Allo738 and Allo733 or Aar4 (bioRxiv, 10.1101/2020.08.24.264432) and Asu and Aar4. Local variants (SNP and indel) were identified in one-to-one alignment region using the dnadiff function of MUMmer^101^.

### Variant calling and phylogenetic analysis

Paired-end resequence reads of 39 *A. arenosa* and 15 *A. suecica* were downloaded from NCBI Short Reads Archive (PRJNA309923 and PRJNA284572)^17^. Downloaded reads and the reads of Asu, Allo733 and Allo738 were filtered using Trimmomatic (v.0.39) with parameters (TruSeq3-PE.fa:2:30:10:8:true LEADING:20 TRAILING:20 SLIDINGWINDOW:5:20 MINLEN:50)^102^. Clean reads of *A. arenosa* were mapped to the Aar assembly and reads of *A. suecica*, Allo733 and Allo738 were mapped to the combination of Aar and At Col (TAIR10) assembly by BWA program (v.0.7.17-r1188) with default parameters. Only uniquely mapped paired reads (-f 3 -q 10) were used for analysing sequence variation and polymerase chain reaction (PCR) duplicates were removed using Picard Toolkit (v.2.18.15) with default parameters (Broad Institute, GitHub Repository http://broadinstitute.github.io/picard/, 2019). Variant was called through the Genome Analysis Toolkit (GATK, v.4.1.3.0) with parameters (--min-base-quality-score 25 && “QD < 2.0 || MQ < 40.0 || FS > 60.0 || SOR > 4.0 || MQRankSum < −12.5 || ReadPosRankSum < −8.0”--filter-name “Fail” -G-filter “DP < 5” -G-filter-name “LowDP” -G-filter “GQ < 20” -G-filter-name “LowGQ” -G-filter “isHet == 1” -G-filter-name “isHetFilter” for SNP filter && “QD < 2.0 || FS > 200.0 || SOR > 10.0 || InbreedingCoeff < −0.8 || ReadPosRankSum < −20.0”–filter-name “Fail” -G-filter “DP < 5” -G-filter-name “LowDP” -G-filter “GQ < 20” -G-filter-name “LowGQ” -G-filter “isHet == 1” -G-filter-name “isHetFilter” for InDel filter). Finally, we generated variants of A genome and T genomes, respectively. Variants of 1,035 individuals^17^ and of T subgenome of *A. suecica*, Allo733 and Allo738 were merged to the final variant file of T genome. Independent SNPs from A genome with minor allele frequency (MAF) < 0.05 and missing rate >0.05 were filtered by PLINK (v.1.9) with parameters (--geno 0.05 --maf 0.05 && --indep-pairwise 50 10 0.2)^103^. SNPs of the T genome were filtered using the same criteria except for missing rate >0.02. The filtered SNPs were used to construct phylogenetic trees by the neighbour-join method in TASSEL (v.5.0)^104^ and visualized using iTOL^105^.

### Identification of rearrangements and local differences

We used MUMmer (v.4.0.0beta2)^101^ with parameters (nucmer --mum -l 50 -c 100 -b 500 -g 100 && delta-filter -l 100 -i 90) for the whole-genome alignment of *A. suecica* and the combination of its assumed progenitors, *A. thaliana* and *A. arenosa*, to identify local and high-order variation. Local variants (SNP and indel) were identified in one-to-one alignment region using the dnadiff function of MUMmer^101^. High-order variation was analysed using SyRI (v.1.1)^106^.

### Syntenic analysis

Synthetic blocks were identified by MCscan (Python version) of jcvi (v.0.8.12) (10.5281/zenodo.31631) (parameters: --cscore = .99) with 30 genes spanned per block^107^.

### Identification of orthologous genes for *K*_a_/*K*_s_ calculations and phylogenetic inference

Orthologous gene clusters were recognized using OrthoFinder^108^ (v.2.2.7) with parameters (-S diamond -M msa -T raxml)^109^. Single-copy genes of *A. thaliana*, *A. arenosa*, *A. suecica* and *A. lyrata* were used to calculate *K*_s_, *K*_a_ and *K*_a_/*K*_s_ values^110^ by KaKs_Calculator (v.1.2)^111^. For gene family analysis, single-copy genes of *A. thaliana*, *A. arenosa*, *A. suecica*, *A. lyrata* and *A. halleri* were extracted using OrthoFinder^108^ (v.2.2.7) and parameters (-S diamond -M msa -T raxml)^109^ and r8s (v.1.81) were used to estimate divergence time to construct phylogenetic trees^112^ with the constrained divergence time range following TimeTree^113^. Contraction and expansion of gene families were identified by CAFE (v.4.2.1) (parameters: -p 0.05 -filter)^114^, which accounted for phylogenetic history and provided a statistical basis for evolutionary inference. *P* values were used to estimate the likelihood of the observed sizes given average rates of gain and loss and used to determine expansion or contraction for individual gene families in each node.

### Small RNA-seq data analysis

Small RNA data were collected in rosette leaves before bolting for *A. thaliana*, *A. arenosa*, F_1_, Allo733 and *A. suecica*^45^ and downloaded from NCBI (GSE15443). Small RNA reads were mapped onto Allo738 genome using ShortStack (v.3.8.5)^115^.

### mRNA-seq data analysis

Total RNA was isolated from rosette leaves (6–7 weeks old), seedlings, flowers and fruit pods in Allo738 and *A. suecica* and was used for messenger RNA sequencing with three biological replicates with ~6.5 Gb per replicate on Illumina HiSeq X Ten platform. The mRNA-seq data were also collected for *A. thaliana*, *A. arenosa*, F_1_, Allo733, *A. suecica* from (GSE29687)^116^ and (GSE50715)^27^. Low-quality reads were filtered using Trimmomatic (v.0.39) with parameters (TruSeq3-PE.fa:2:30:10:8:true LEADING:20 TRAILING:20 SLIDINGWINDOW:5:20 MINLEN:50)^102^. To exclude expression bias between *A. thaliana* and *A. arenosa* due to depth difference, reads of *A. thaliana* and *A. arenosa* were down-sampled to the same level and combined. Reads of *A. thaliana*, *A. arenosa*, F_1_, Allo733 and *A. suecica* were mapped to the Allo738 genome along with the SNP table of Asu and Allo733 genomes, respectively, using HISAT2 and StringTie^117^ with parameters (--score-min L, 0.0,−0.4). Reads of Allo738 were mapped to the Allo738 genome using HISAT2 and StringTie with default parameters. Only uniquely mapped reads were kept for further analysis. The expression level of each gene was calculated using StringTie. We selected homologous genes between Asu and Allo738 for expression between allotetraploid species and homologous gene pairs between A and T subgenomes for expression within an allotetraploid.

### MethylC-seq data analysis

Total genomic DNA was extracted from rosette leaves before bolting (3–4 weeks for *A. thaliana* and 6–7 weeks for *A. arenosa*, F_1_, Allo733, Allo738 and *A. suecica*). MethylC-seq libraries were constructed using a bisulfite method as previously described^53^ and sequenced on Illumina HiSeq X Ten platform (~11 Gb per replicate). Low-quality reads were filtered using Trimmomatic (v.0.39) with parameters (TruSeq3-PE.fa:2:30:10:8:true LEADING:20 TRAILING:20 SLIDINGWINDOW:5:20 MINLEN:50)^102^. MethylC-seq reads of *A. suecica* and Allo738 were mapped to the *A. suecica* and Allo738 genome using Bismark (v.0.15.1) with parameters (--score_min L,0,−0.2), respectively^118^. MethylC-seq reads of *A. thaliana*, *A. arenosa*, F_1_, Allo733 were mapped to the Allo738 genome using Bismark (v.0.15.1) with parameters (--score_min L,0,−0.4). Reads of Allo733 were mapped onto the Allo733-SNP-substituted Allo738 genome. To remove bias, only the uniquely mapping reads and conserved cytosines were used for downstream analyses following a previous method^53^ (also see Github: https://github.com/Anticyclone-op/Ara-genome-methly). To identify conserved regions of 1 kb or longer in *A. suecica* and Allo738, we aligned the *A. suecica* genome against the Allo738 genome by LAST (v.869) (parameters: last -q3 -m50 -e35 -P10 && last-split -m1 -s200)^119^ and then swapped the sequences and extracted the best alignments. Finally, alignments with scores <1,000 were removed. The conserved cytosines between *A. suecica* and Allo738 were extracted using Python scripts. The same method was used to identify the conserved region and conserved cytosines between the A and T subgenomes. Shared methylation sites in two replicates were merged for further analysis.

DMRs between the T subgenome and *A. thaliana* or between the A subgenome and *A. arenosa* were analysed using 100-bp sliding windows, including four or more cytosines for CG and CHG contexts and 16 or more cytosines for CHH context. The hyper- and hypo-DMRs mean allotetraploid relative to parent. The weighted methylation level was calculated for each window. Significant differences were assessed using Fisher’s exact test (FDR < 0.05), using the following cutoff values of the minimum difference of the methylation levels: 0.5 for CG DMRs, 0.3 for CHG DMRs and 0.1 for CHH DMRs. For DMRs between A and T genomes and in F_1_, Allo733, Allo738 and Asu, using the same criteria, either as hyper-DMRs (A > T) or hypo-DMRs (T < A). DMR-overlapping genes were defined as those that overlapped with DMRs within a 2-kb region. Conserved DMRs were defined as the hypo-DMRs in Asu and consistently present in F_1_, Allo733 or Allo738. Convergent DMRs were identified as the hyper-DMRs between Aar and Ath and in F_1_ and resynthesized allotetraploids and decreased to a similar level to the T subgenome in Asu, while the overlap between two groups represented those DMRs convergent in newly formed allotetraploid and remained in Asu.

### Reporting Summary

Further information on research design is available in the Nature Research Reporting Summary linked to this article.

## Supplementary information

Supplementary Information Supplementary text, Figs. 1–4 and refs. 1–9.

Reporting Summary

Peer Review Information

Source Data Supplementary Figs. 1–4.

## Data Availability

Sequencing data are accessible under NCBI BioProject numbers (PRJNA669593). All datasets generated and/or analysed in the study are available in the main text, Table 1, Figs. 1–5, Extended Data Figs. 1–10, Supplementary Information and the Reporting Summary. Source data are provided with this paper.

## Source data

Source Data Fig. 1 Statistical source data.
Source Data Fig. 2 Statistical source data.
Source Data Fig. 3 Statistical source data.
Source Data Fig. 4 Statistical source data.
Source Data Fig. 5 Statistical source data.
Source Data Extended Data Fig. 1 Statistical source data.
Source Data Extended Data Fig. 2 Statistical source data.
Source Data Extended Data Fig. 3 Statistical source data.
Source Data Extended Data Fig. 4 Statistical source data.
Source Data Extended Data Fig. 5 Statistical source data.
Source Data Extended Data Fig. 6 Statistical source data.
Source Data Extended Data Fig. 7 Statistical source data.
Source Data Extended Data Fig. 8 Statistical source data.
Source Data Extended Data Fig. 9 Statistical source data.
Source Data Extended Data Fig. 10 Statistical source data.
