# Supplementary material for: Concerted genomic and epigenomic changes accompany stabilization of Arabidopsis allopolyploids
Source: Nat Ecol Evol. 2021 Aug 19;5(10):1382–93. doi: 10.1038/s41559-021-01523-y (PMC8484014; doi:10.1038/s41559-021-01523-y)
Supplement: Supplementary file 1 — Supplementary text, Figs. 1–4 and refs. 1–9. [file 41559_2021_1523_MOESM1_ESM.pdf]

nature portfolio

https://doi.org/10.1038/s41559-021-01523-y

Supplementary information

Concerted genomic and epigenomic changes accompany stabilization of *Arabidopsis* allopolyploids

In the format provided by the authors and unedited

Concerted genomic and epigenomic changes accompany stabilization of *Arabidopsis* allopolyploids

Xinyu Jiang[1], Qingxin Song[1,2], Wenxue Ye[1], and Z. Jeffrey Chen[1,2] *

[1]State Key Laboratory of Crop Genetics and Germplasm Enhancement, Nanjing Agricultural University, 1 Weigang Road, Nanjing 210095, China

[2]Department of Molecular Biosciences, The University of Texas at Austin, Austin, TX 78712, USA

Short title: Methylation and sequencing in *Arabidopsis* allotetraploids

Keywords: Polyploidy, Sequencing, DNA Methylation, Gene Expression, Evolution

*Corresponding author: zjchen@austin.utexas.edu

Genomic stability of resynthesized allotetraploids

We sequenced another neo-allotetraploid Allo733 (Supplementary Fig. 1) and compared Allo733 and Allo738 genomes with L*er*[1] and other *Arabidopsis* species[2,3] including *A. arenosa* (Aar) accession (https://doi.org/10.1101/2020.08.24.264432). We found that (1) Allo733 and Allo738 had similar levels of divergence to L*er* and Aar, respectively (Supplementary Figure 1a); (2) A subgenome of Allo733 and Allo738 was closely related to *A. arenosa* accessions that were in a different clade from A subgenomes of *A. suecica* accessions (Supplementary Figure 1b); and (3) T subgenome of Allo733 and Allo738 was closely related to L*er*, which was different from T subgenome of *A. suecica* accessions (Supplementary Figure 1c). Neighbor-joining evolutionary tree also indicated that the A-subgenome donor of Allo733 and Allo738 was closest to *A. arenosa* [3] (Supplementary Figure 1b), and T-subgenome donor of Asu was closely related to ecotypes from Russia-Asia admixture among 1,135 strains analyzed (Supplementary Figure 1c)[2,4]. At a fine-scale level, we found that frequencies of SNPs and indels were very low (0.04 SNPs and 0.04 indels per kb) in the T subgenome between the two resynthesized allotetraploids Allo733 and Allo738, while they were higher (2.15 SNPs and 1.81 indels per kb) in the A subgenome; these levels of variation were also comparable with their corresponding extant parents, L*er* and Aar4 (Supplementary Tables 1).

S locus evolution in allopolyploids

S locus genes were more polymorphic within than between species (Supplementary Fig. 2a), suggesting a long-term balanced selection, leading to highly diverged S-alleles shared across

species[3,5]. In *A. thaliana* and related species, all SRKs are functional (Supplementary Fig. 2b)[6]. *A. suecica* has two additional *SRK* genes related to *A. halleri AhSRK4* (T) and *AhSRK2* (A) haplotypes, respectively (Supplementary Fig. 2a). In male components, *SCR* genes were polymorphic between *A. suecica* and other species (Supplementary Fig. 2c). Notably, different S locus haplotypes have a hierarchical dominance relationship [7]. The self-compatible *A. thaliana SCR04* is dominant over *A. arenosa SCR01* alleles of Allo733 and Allo738 (Supplementary Fig. 2c), probably through targeting the 5' UTR of *AaSCR01* by *A. thaliana* miR1887 of *SCR04* (Supplementary Fig. 2d). Moreover, *AaSCR02* allele was silenced by miR867 of *SCR04* targeting the first exon of *AaSCR02* with a frameshift mutation (Supplementary Figure 2d)[3]. RNA-seq data showed that *AaSCR* was not expressed in flowers of both Allo738 and Asu, while *AtSCR* expression level was very low in Allo738 and undetectable in Asu, supporting silencing of *AaSCR* genes in these allotetraploids (Supplementary Fig. 2e).

Reproduction-associated genes

We analyzed the *Boy Named Sue* (*BYS*) locus[8]. Among 56 annotated genes, *FIS2* is absent in *A. lyrata* and has variable sequences in *A. arenosa* and *A. suecica* and is a likely candidate (Supplementary Fig. 3). In addition, hypermethylation of reproduction-associated genes may lead to gene loss. For example, *ASY2* (asynaptic mutant2), a homolog of *ASY1*[9], is heavily methylated and poorly expressed in Allo733, Allo738, and *A. suecica* and possesses a frameshift mutation, which are not observed in *A. thaliana* or *A. arenosa* (Supplementary Fig. 4).

References

1. Zapata, L. et al. Chromosome-level assembly of Arabidopsis thaliana Ler reveals the extent of translocation and inversion polymorphisms. *Proc Natl Acad Sci U S A* **113**, E4052-60 (2016).
2. Novikova, P.Y. et al. Sequencing of the genus Arabidopsis identifies a complex history of nonbifurcating speciation and abundant trans-specific polymorphism. *Nat Genet* **48**, 1077-82 (2016).
3. Novikova, P.Y. et al. Genome Sequencing Reveals the Origin of the Allotetraploid Arabidopsis suecica. *Mol Biol Evol* **34**, 957-968 (2017).
4. Consortium, G. 1,135 Genomes Reveal the Global Pattern of Polymorphism in Arabidopsis thaliana. *Cell* **166**, 481-491 (2016).
5. Mable, B.K. Breaking down taxonomic barriers in polyploidy research. *Trends in Plant Science* **8**, 582-590 (2003).
6. Tsuchimatsu, T. et al. Evolution of self-compatibility in Arabidopsis by a mutation in the male specificity gene. *Nature* **464**, 1342-6 (2010).

7. Durand, E. et al. Dominance hierarchy arising from the evolution of a complex small RNA regulatory network. *Science* **346**, 1200-5 (2014).
8. Chaudhury, A.M. et al. Fertilization-independent seed development in Arabidopsis thaliana. *Proc Natl Acad Sci U S A* **94**, 4223-8 (1997).
9. Caryl, A.P., Armstrong, S.J., Jones, G.H. & Franklin, F.C. A homologue of the yeast HOP1 gene is inactivated in the Arabidopsis meiotic mutant asy1. *Chromosoma* **109**, 62-71 (2000).

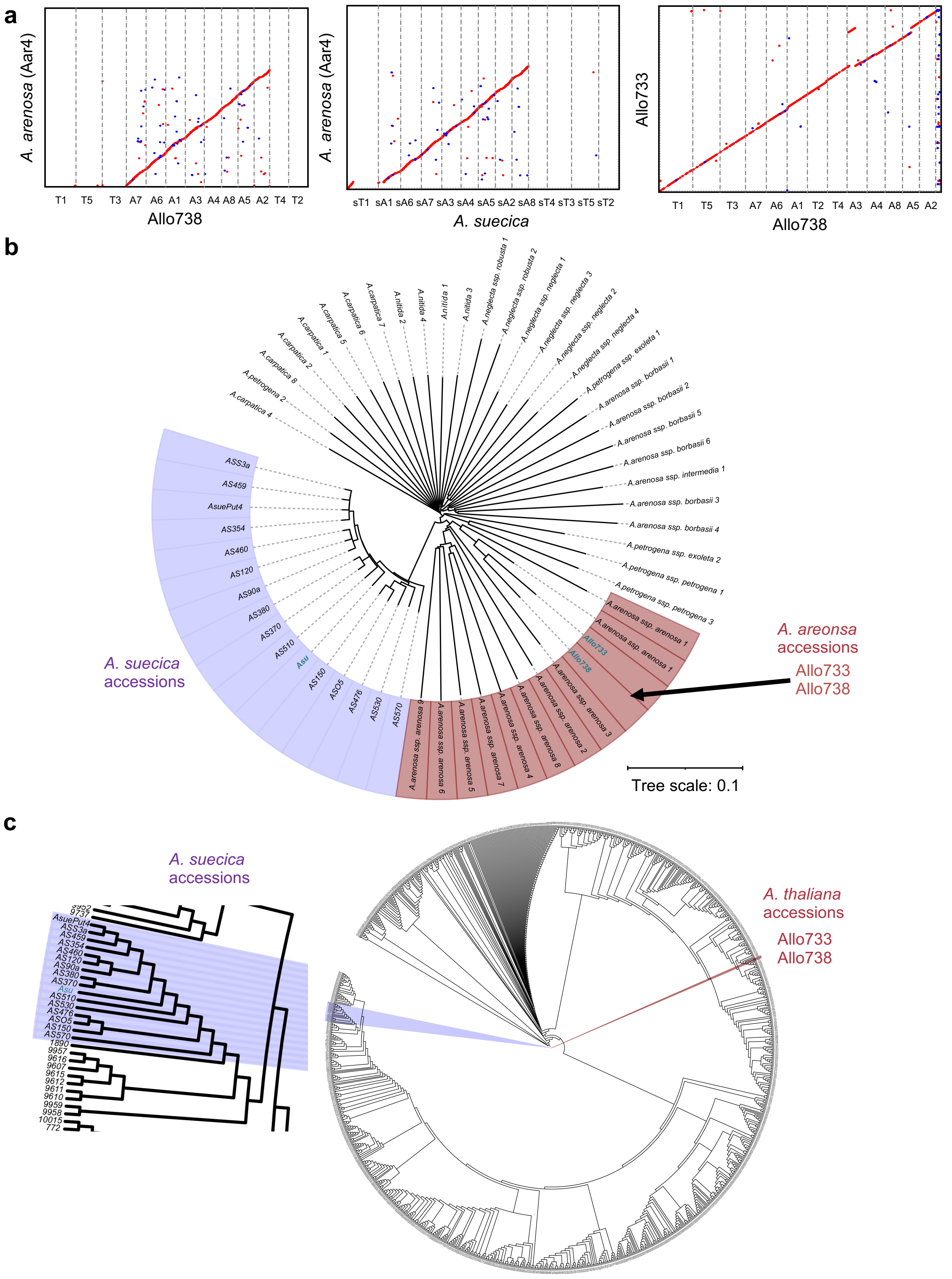

Supplementary Fig. 1 - Genome stability in Allo738 and Allo733 and origin of sA and sT subgenomes in A. suecica.

a, Dotplots of genome assemblies between *A. arenosa* (Aar4) and Allo738 (left) or *A. suecica* (Asu) (middle) and between Allo733 and Allo738 (right). Colinear (red line) or disrupted (blue line or dots) regions are shown. **b** and **c,** Clustering of sequenced genomes using polymorphism data from *A. arenosa* accessions (**b**) and *A. thaliana* accessions (**c**). Clades of *A. suecica* accessions (purple) and Allo733 and Allo738 (brown) are indicated. Branch lengths in (**c**) may not represent phylogenetic distances due to missing data.

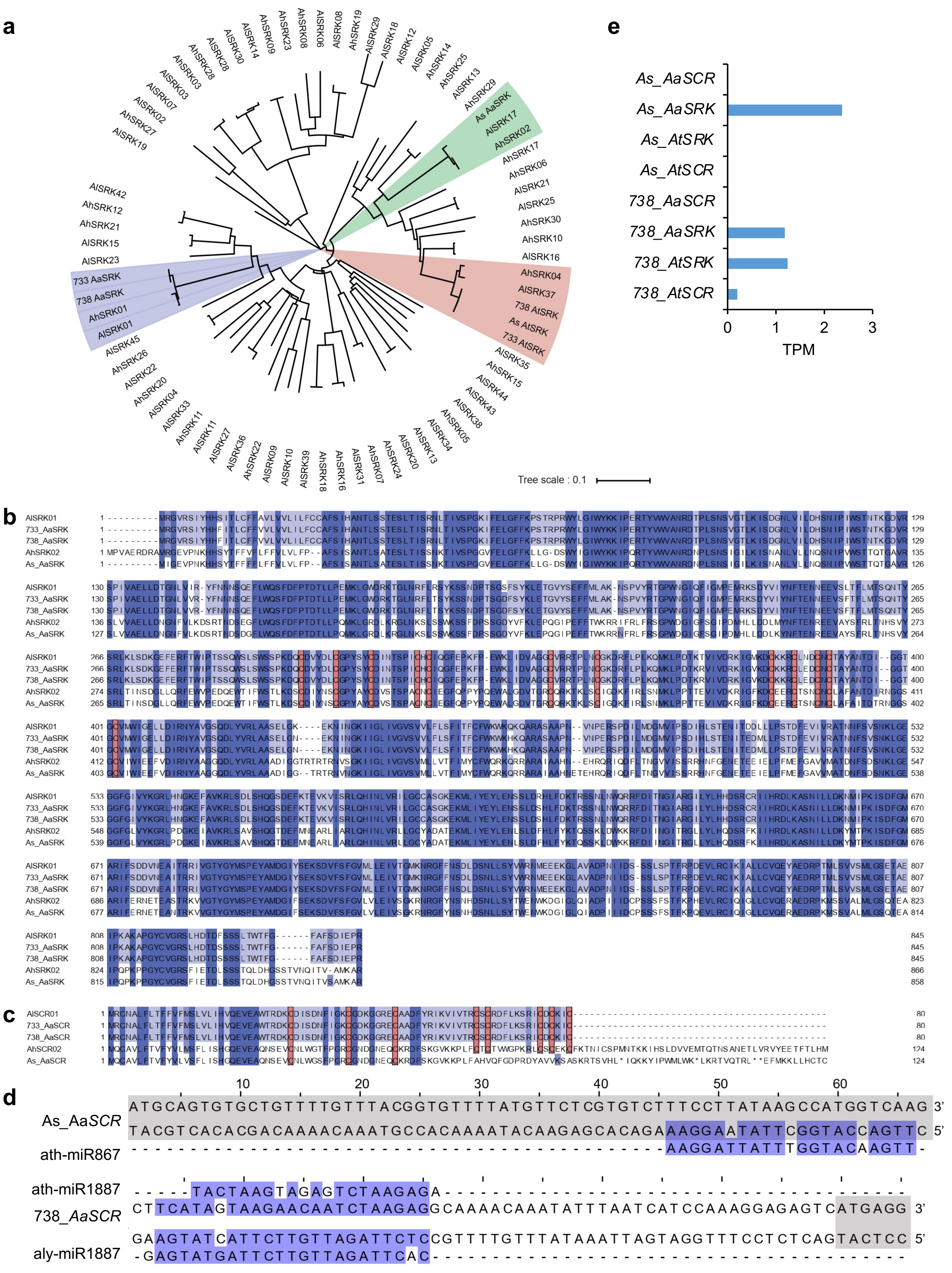

Supplementary Fig. 2 - Sequences analyses of SCR and SRK genes and miRNA targets of AaSCR genes. **a**, Phylogenetic tree of *SRK* genes (neighbor-joining tree). Colors indicate AhSRK01 (purple), AhSRK02 (green), and AhSRK04 (red) haplotypes. Aha: *A. halleri*; Aly: *A. lyrata*; Aar: *A. arenosa*; Ath: *A. thaliana*. **b**, **c**, Multiple protein sequence alignment of *SRK* (**b**) and *SCR* (**c**) genes in A and sA subgenome of Asu. **d**, Ath-miR867 targets the first exon (grey box) of *A. suecica SCR02* (*As_AaSCR*). Ath-miR1887 and *A. lyrata* miR1887 (aly-miR1887) target the 5' untranscribed region (UTR) of *SCR01* in Allo733 and Allo738 (738_*AaSCR*). **e**, *SCR* and *SRK* expression levels (TPM) in open flowers of resynthesized allotetraploid (Allo738) and Asu.

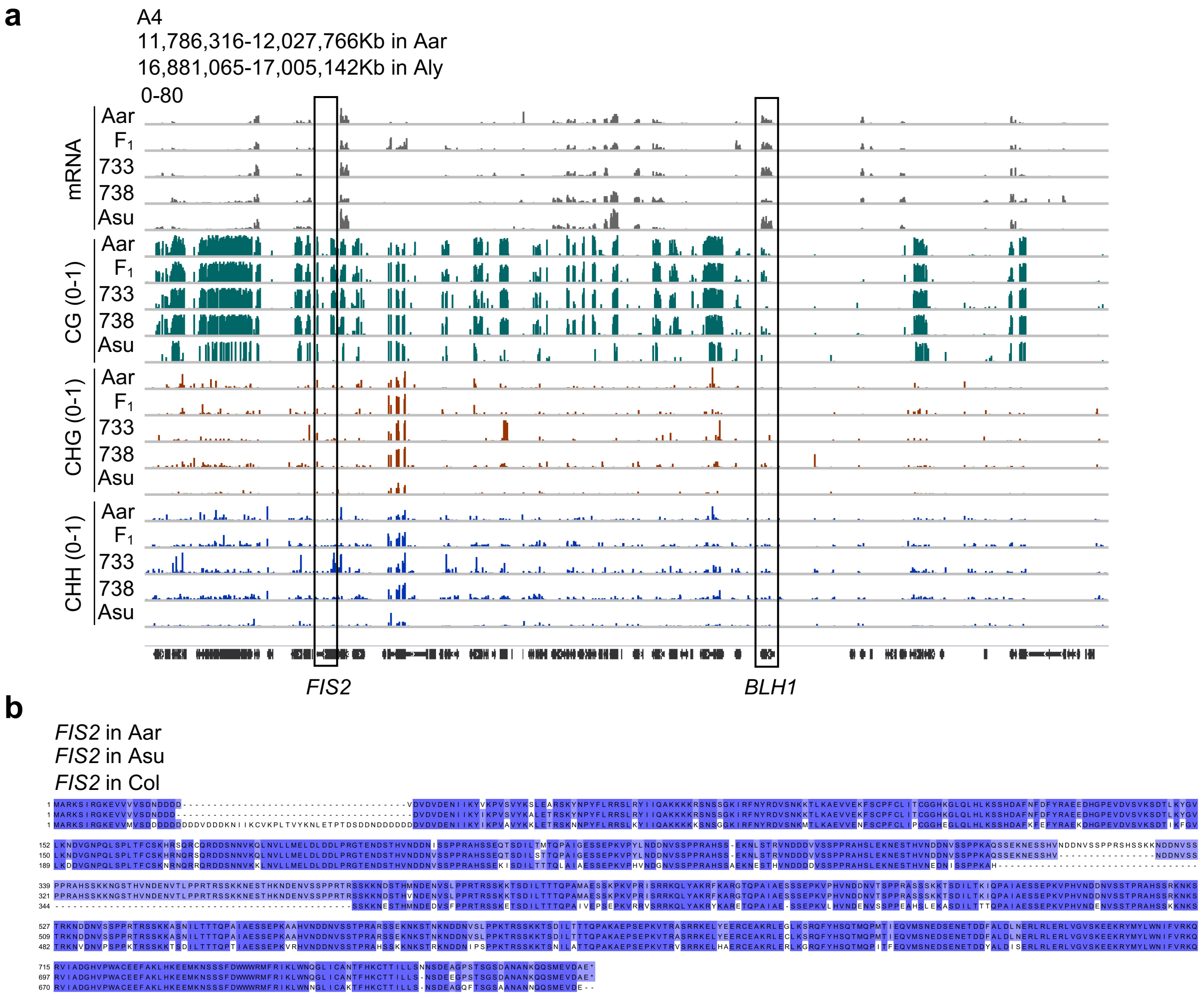

Supplementary Fig. 3 - Expression and methylation levels of BYS locus and sequences analyses of a candidate gene FIS2.

a, Methylation and mRNA expression patterns near *BYS* locus (11.8-12Mb of A4) in *A. arenosa* (Aar), F_1, Allo733 (733), Allo738 (738), and natural *A. suecica* (Asu). Scales indicate mRNA (0-80) and methylation density (0-1) levels. The black boxs indicate the candidate genes of *BYS* locus. **b**, Multiple protein sequence alignment of *FIS2* genes in *A. arenosa, A. thaliana* and A subgenome of *A. suecica*.

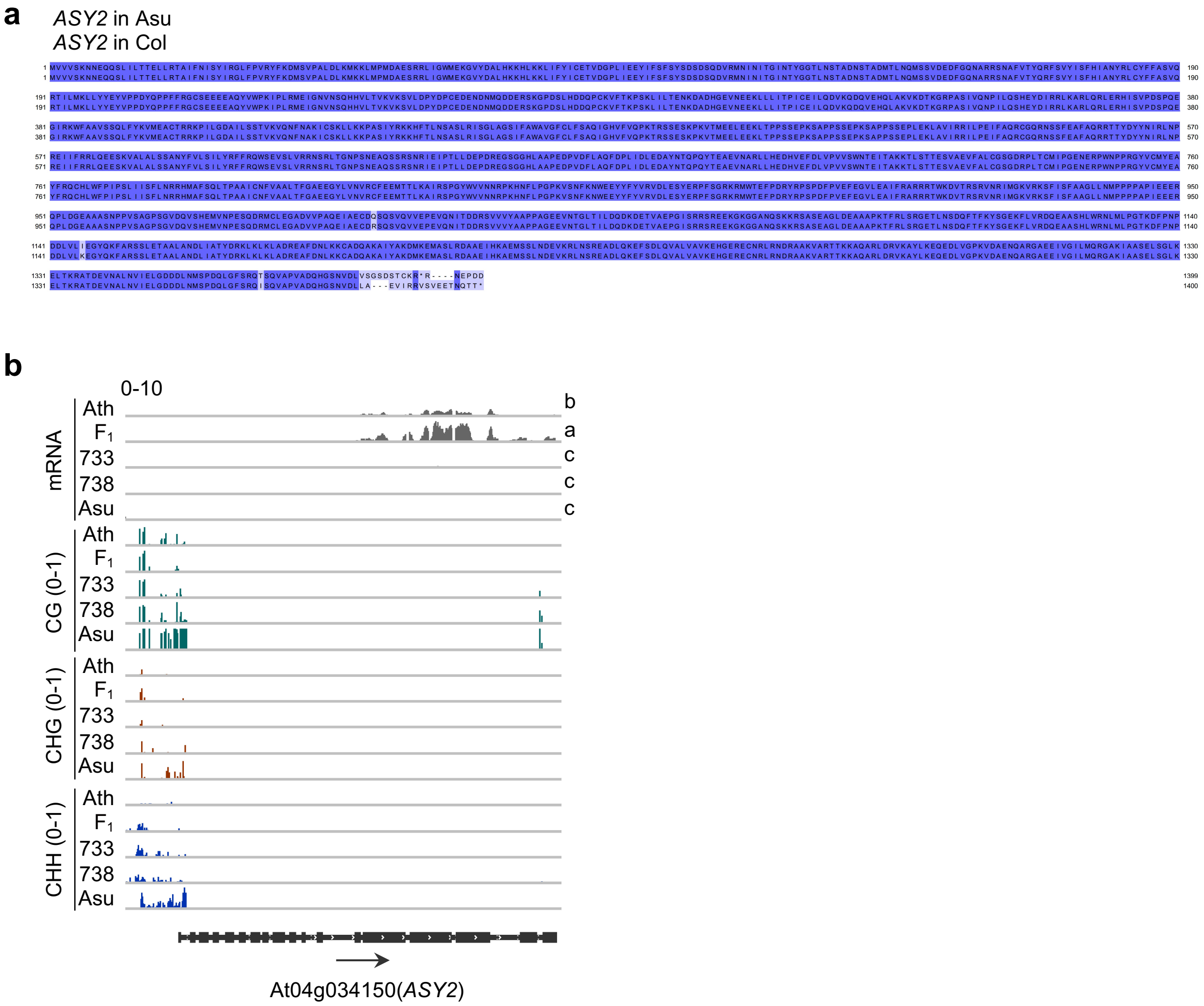

Supplementary Fig. 4 - Sequences analyses of ASY2 genes and expression and methylation levels of AtASY2.

a, Multiple protein sequence alignment of *ASY2* genes in *A. thaliana* and T subgenome of *A. suecica*. **b**, Methylation near genic regions of *ASY2* and its mRNA expression patterns in *A. thaliana* (Ath), F_1, Allo733 (733), Allo738 (738), and natural *A. suecica* (Asu). Scales indicate mRNA (0-10) and methylation density (0-1) levels. Different letters indicate statistical significance (ANOVA test with three biological replications).
