# Supplementary material for: Concerted genomic and epigenomic changes accompany stabilization of Arabidopsis allopolyploids
Source: Nat Ecol Evol. 2021 Aug 19;5(10):1382–93. doi: 10.1038/s41559-021-01523-y (PMC8484014; doi:10.1038/s41559-021-01523-y)
Supplement: Supplementary file 2 — Reporting Summary [file 41559_2021_1523_MOESM2_ESM.pdf]

nature research

Corresponding author(s): Z. Jeffrey Chen

Last updated by author(s): Feb 20, 2021

Reporting Summary

Nature Research wishes to improve the reproducibility of the work that we publish. This form provides structure for consistency and transparency in reporting. For further information on Nature Research policies, see our Editorial Policies and the Editorial Policy Checklist.

Statistics

For all statistical analyses, confirm that the following items are present in the figure legend, table legend, main text, or Methods section.

n/a	Confirmed	
☐	☒	The exact sample size (*n*) for each experimental group/condition, given as a discrete number and unit of measurement
☐	☒	A statement on whether measurements were taken from distinct samples or whether the same sample was measured repeatedly
☐	☒	The statistical test(s) used AND whether they are one- or two-sided<br>*Only common tests should be described solely by name; describe more complex techniques in the Methods section.*
☒	☐	A description of all covariates tested
☐	☒	A description of any assumptions or corrections, such as tests of normality and adjustment for multiple comparisons
☐	☒	A full description of the statistical parameters including central tendency (e.g. means) or other basic estimates (e.g. regression coefficient) AND variation (e.g. standard deviation) or associated estimates of uncertainty (e.g. confidence intervals)
☐	☒	For null hypothesis testing, the test statistic (e.g. *F*, *t*, *r*) with confidence intervals, effect sizes, degrees of freedom and *P* value noted<br>*Give P values as exact values whenever suitable.*
☒	☐	For Bayesian analysis, information on the choice of priors and Markov chain Monte Carlo settings
☒	☐	For hierarchical and complex designs, identification of the appropriate level for tests and full reporting of outcomes
☐	☒	Estimates of effect sizes (e.g. Cohen's *d*, Pearson's *r*), indicating how they were calculated

Our web collection on statistics for biologists contains articles on many of the points above.

Software and code

Policy information about availability of computer code

Data collection

(1) DNA sequencing was performed using Illumina X10, PacBio - SEQUEL and corresponding software from the manufacturers.
(2) RNA-seq data were generated using Illumina X10 (2X150 bp paired-end reads) and its software.
(3) Methylome (MethylC seq) data were generated using paired-end sequencing using Illumina X10.
(4) Hi-C sequencing was performed using Illumina X10 (2X150 bp paired-end reads), and reads were mapped using Juicer (v1.6.2).

Data analysis

(1) Assembly and annotation: We used MECAT (v1.0), ARROW (v5.0.1.9585), Pillon (v1.22), Juicer (v1.5.6), 3D-DNA (v180114) and Juicebox (v1.9.0) for genome assembly. Following tools were used for genome annotation: Augustus (v3.2.2); TransDecoder (v5.3.0); PASA (v2.3.3); Exonerate (v2.2.0); EVidenceModeler (v1.1.1); InterProScan (v 5.32-71.0); RepeatModeler (v1.0.11); Repeatmasker (v4.0.7); LTR_retriever (v2.0); LTR-FINDER (v1.07); infernal (v1.1.2); tRNAscan-SE (v2.0).

(2) Assessment of genome completeness: We evaluated the genome assembly completeness by BUSCO (v3.0.2) and the accuracy of the assembly through whole-genome alignment against the reference genome of A. thaliana (TAIR10) or A. lyrata (Alyrata_384_v2.1) by MUMmer (v4.0.0beta2). Genome comparisons using HiC data: HiC libraries A. suecica (Asu) and A. thaliana x A. arenosa (Allo738) were aligned to published Ath and Aly reference genomes using BWA-MEM. Heatmaps were generated using the JUICER-pre command, and visualized using JUICEBOX. Inversions and rearrangements were further identified using JUICEBOX.

(3) Analysis of chromosomal collinearity, structural rearrangements and gene family composition between A. suecica and the combination of its assumed progenitors, A. thaliana and A. arenosa: Ath (TAIR10) and Aar (A subgenome of Allo738) assemblies were aligned to the Asu assemblies generated in this study using MUMmer with parameters (nucmer --mum -l 50 -c 100 -b 500 -g 100 && delta-filter -l 100 -i 90). The resulting alignments were used to identify structural rearrangements and local variations using SyRI. Synthetic blocks were identified by MCscan of jcvi (v0.8.12). The gene copy numbers and gene families between assemblies were identified using OrthoFinder based on all annotated protein coding sequences.

(4) LTR analyses: LTR-FINDER (v1.07) and LTR-harvest (v1.5.10) was used to identify full-length retrotransposons. LTR-retriever was used to

integrate those TEs generated by LTR-finder and LTR-harvest, as well as to predict the TE insertion time using the Arabidopsis mutation rate (r=7x10-9). Box plots of insertion time were generated using ggplot2 in R.

(5) Analysis of orthologs and homoeologs: We used diamond (v0.9.24) and OrthoFinder (v2.2.7) to identify homoeologous and orthologous sequences. GO functional enrichment analysis was performed using the clusterProfiler R package.

(6) The non-synonymous/synonymous (Ka/Ks) values estimate: The 14,668 orthologs pairs of each Arabidopsis species were used for estimating Ka/Ks values by KaKs_Calculator (v1.2).

(7) Evolutionary analysis: We used OrthoFinder (v2.2.7), RAxML (v8.2.11), r8s (v1.81) and CAFE (v4.2.1) for phylogenetic analysis and contraction and expansion of gene families estimates. Domain enrichment analysis of contraction/expansion gene families using a Fisher's-exact-test and FDR correction for multiple test.

(8) RNA-seq analysis of homoeolog expression: To exclude expression bias between Ath and Aar due to depth difference, reads of Ath and Aar were down-sampled to the same level and combined. Reads of Ath, Aar, F1, Allo733, and A. suecica were mapped to the Allo738 genome using HISAT2 (v2.1.0) (--score-min L, 0.0,-0.4). Reads of Allo738 were mapped to the Allo738 genome using HISAT2 with default parameters. Only uniquely mapped reads were kept for further analysis. The expression level of each gene was calculated using StringTie (v1.3.3b).

(9) MethylC seq analyses: MethylC-seq reads of Asu and Allo738 were mapped to the Asu and Allo738 genome using Bismark (v0.15.1) with parameters (--score_min L,0,-0.2), respectively. MethylC-seq reads of Ath, Aar, F1, Allo733 were mapped to the Allo738 genome using Bismark with parameters (--score_min L,0,-0.4). To remove bias, only the conserved cytosines were used for downstream analyses using custom Python scripts. To identify conserved regions of 1 kb or longer in A. suecica and Allo738, we aligned the Asu genome against the Allo738 genome by LAST (v869), swapped the sequences and extracted the best alignments. Finally, alignments with scores less than 1000 were removed. The same method was used to identify the conserved region and conserved cytosines between the A and T subgenomes. Differentially methylated regions (DMRs) between the T subgenome and Ath or between the A subgenome and Aar were analyzed using 100-bp sliding windows, including four or more cytosines for CG and CHG contexts and sixteen or more cytosines for CHH context. The weighted methylation level was calculated for each window. Significant differences were assessed using Fisher's-exact-test and FDR correction for multiple test (FDR<0.05), using the following cut-off values of the methylation levels: 0.5 for CG DMRs, 0.3 for CHG DMRs, and 0.1 for CHH DMRs.

(10) Variation calling and Phylogenetic analysis: The paired-end resequencing reads of 39 A. arenosa and 15 A. suecica were downloaded from PRJNA309923 and PRJNA284572 in NCBI Short Reads Archive. The downloaded reads and the reads of Asu, Allo733 and Allo738 were filtered using Trimmomatic (version 0.39). The clean reads of A. arenosa were mapping to the Aar assembly and reads of A. suecica, Allo733 and Allo738 were mapping to the combination of Aar and TAIR10 assembly by BWA program (version 0.7.17-r1188). Only uniquely mapped paired reads were used for the detection of genetic variation and remove PCR duplicates using Picard (version 2.18.15). Variation was called through the Genome Analysis Toolkit (GATK, version 4.1.3.0). Finally, we generate variants of A genome and T genome separately. The variants of 1035 individuals and the variants of T subgenome of A. suecica, Allo733 and Allo738 were merged to the final variants file of T genome. The independent SNPs from A genome with MAF>0.05, missing rate >0.05 were filtered by PLINK (version 1.9). While for SNPs of T genome were filtered same with A genome except the missing rate > 0.02. The filtered SNPs were used to construct phylogenetic trees using the Neighbor-Join method in TASSEL (version 5.0).

For manuscripts utilizing custom algorithms or software that are central to the research but not yet described in published literature, software must be made available to editors and reviewers. We strongly encourage code deposition in a community repository (e.g. GitHub). See the Nature Research guidelines for submitting code & software for further information.

Data

Policy information about availability of data

All manuscripts must include a data availability statement. This statement should provide the following information, where applicable:

- Accession codes, unique identifiers, or web links for publicly available datasets
- A list of figures that have associated raw data
- A description of any restrictions on data availability

SUBID	BioProject	BioSample	Accession	Organism
SUB8369755	PRJNA669593	SAMN16534086	JAEFBK000000000	Arabidopsis arenosa x Arabidopsis thaliana (Allo738)
SUB8369755	PRJNA669593	SAMN16534085	JAEFBJ000000000	Arabidopsis suecica (As)
SUB8902864	PRJNA669593	SAMN17369459	JAESVC000000000	Arabidopsis arenosa x Arabidopsis thaliana (Allo733)
SUB8323092	PRJNA669593	SAMN16456086	SRR12880892	Allo738_seedling_RNA-seq
SUB8323092	PRJNA669593	SAMN16456085	SRR12880893	As_pod_RNA-seq
SUB8323092	PRJNA669593	SAMN16456084	SRR12880894	As_flower_RNA-seq
SUB8323092	PRJNA669593	SAMN16456083	SRR12880895	As_seedling_RNA-seq
SUB8323092	PRJNA669593	SAMN16456082	SRR12880896	Allo738_HiC-seq
SUB8323092	PRJNA669593	SAMN16456081	SRR12880897	As_HiC-seq
SUB8323092	PRJNA669593	SAMN16456080	SRR12880898	Allo738_DNA-seq
SUB8323092	PRJNA669593	SAMN16456103	SRR12880899	As_BS-seq_rep2
SUB8323092	PRJNA669593	SAMN16456102	SRR12880900	As_BS-seq_rep1
SUB8323092	PRJNA669593	SAMN16456101	SRR12880901	Allo738_BS-seq_rep2
SUB8323092	PRJNA669593	SAMN16456100	SRR12880902	Allo738_BS-seq_rep1
SUB8323092	PRJNA669593	SAMN16456099	SRR12880903	Allo733_BS-seq_rep2
SUB8323092	PRJNA669593	SAMN16456098	SRR12880904	Allo733_BS-seq_rep1
SUB8323092	PRJNA669593	SAMN16456097	SRR12880905	F1_BS-seq_rep2
SUB8323092	PRJNA669593	SAMN16456079	SRR12880906	As_DNA-seq
SUB8323092	PRJNA669593	SAMN16456096	SRR12880907	F1_BS-seq_rep1
SUB8323092	PRJNA669593	SAMN16456095	SRR12880908	Aa_BS-seq_rep2
SUB8323092	PRJNA669593	SAMN16456094	SRR12880909	Aa_BS-seq_rep1

SUB8323092 PRJNA669593 SAMN16456093 SRR12880910 At_BS-seq_rep2
SUB8323092 PRJNA669593 SAMN16456092 SRR12880911 At_BS-seq_rep1
SUB8323092 PRJNA669593 SAMN16456091 SRR12880912 Allo738_leaf_RNA-seq_rep3
SUB8323092 PRJNA669593 SAMN16456090 SRR12880913 Allo738_leaf_RNA-seq_rep2
SUB8323092 PRJNA669593 SAMN16456089 SRR12880914 Allo738_leaf_RNA-seq_rep1
SUB8323092 PRJNA669593 SAMN16456088 SRR12880915 Allo738_pod_RNA-seq
SUB8323092 PRJNA669593 SAMN16456087 SRR12880916 Allo738_flower_RNA-seq
SUB8323092 PRJNA669593 SAMN16456078 SRR12880917 Allo738_PacBio
SUB8323092 PRJNA669593 SAMN16456077 SRR12880918 As_PacBio
SUB8902848 PRJNA669593 SAMN17371748 SRR13452155 Allo733_PacBio
SUB8902848 PRJNA669593 SAMN17371749 SRR13452154 Allo733_DNA-seq
Note: Assemblies are still in manual review and will be released under those accession numbers.

Field-specific reporting

Please select the one below that is the best fit for your research. If you are not sure, read the appropriate sections before making your selection.

☒ Life sciences ☐ Behavioural & social sciences ☐ Ecological, evolutionary & environmental sciences

For a reference copy of the document with all sections, see nature.com/documents/nr-reporting-summary-flat.pdf

Life sciences study design

All studies must disclose on these points even when the disclosure is negative.

Sample size	Sample size per group or condition was determined based on the minimum number of biological replicates required to perform differential expression and methylation analysis as per software tools used and previously published literature.
Data exclusions	Samples were excluded if they failed at the library preparation stage or those that displayed poor correlation between biological replicates.
Replication	Findings were consistent between biological replicates and different sequencing plates/batches. The replications of methylation data were merged to increase coverage.
Randomization	Order of sample processing for library preparation and sequencing were processed in multiple batches as and when they were received from collaborating laboratories, kind of randomization in itself, but following stringent standardized protocols.
Blinding	No blinding took place. To alleviate any complications from non-blinded analyses all samples were analyzed simultaneously in the same manner regardless of their condition/origin. All specimens' identities were encoded before submission for genotyping.

Reporting for specific materials, systems and methods

We require information from authors about some types of materials, experimental systems and methods used in many studies. Here, indicate whether each material, system or method listed is relevant to your study. If you are not sure if a list item applies to your research, read the appropriate section before selecting a response.

Materials & experimental systems

n/a	Involved in the study	
☒	☐	Antibodies
☒	☐	Eukaryotic cell lines
☒	☐	Palaeontology and archaeology
☒	☐	Animals and other organisms
☒	☐	Human research participants
☒	☐	Clinical data
☒	☐	Dual use research of concern

Methods

n/a	Involved in the study	
☒	☐	ChIP-seq
☒	☐	Flow cytometry
☒	☐	MRI-based neuroimaging
