# Supplementary material for: Concerted genomic and epigenomic changes accompany stabilization of Arabidopsis allopolyploids
Source: Nat Ecol Evol. 2021 Aug 19;5(10):1382–93. doi: 10.1038/s41559-021-01523-y (PMC8484014; doi:10.1038/s41559-021-01523-y)
Supplement: Supplementary file 3 — Peer Review Information [file 41559_2021_1523_MOESM3_ESM.pdf]

natureresearch

Peer Review Information

Journal: Nature Ecology & Evolution
Manuscript Title: Concerted genomic and epigenomic changes accompany stabilization of Arabidopsis allopolyploids
Corresponding author name(s): Z. Jeffrey Chen

Editorial Notes:

Reviewer Comments & Decisions:

Decision Letter, initial version:

17th December 2020

*Please ensure you delete the link to your author homepage in this e-mail if you wish to forward it to your co-authors.

Dear Professor Chen,

Your manuscript entitled "Concerted genomic and epigenomic changes stabilize Arabidopsis allopolyploids" has now been seen by 3 reviewers, whose comments are attached. The reviewers have raised a number of concerns which will need to be addressed before we can offer publication in Nature Ecology & Evolution. We will therefore need to see your responses to the criticisms raised and to some editorial concerns, along with a revised manuscript, before we can reach a final decision regarding publication.

We therefore invite you to revise your manuscript taking into account all reviewer and editor comments. Please highlight all changes in the manuscript text file in Microsoft Word format.

We are committed to providing a fair and constructive peer-review process. Do not hesitate to contact us if there are specific requests from the reviewers that you believe are technically impossible or unlikely to yield a meaningful outcome.

When revising your manuscript:

* Include a “Response to reviewers” document detailing, point-by-point, how you addressed each reviewer comment. If no action was taken to address a point, you must provide a compelling argument. This response will be sent back to the reviewers along with the revised manuscript.

* If you have not done so already please begin to revise your manuscript so that it conforms to our Article format instructions at http://www.nature.com/natecolevol/info/final-submission. Refer also to any guidelines provided in this letter.

* Include a revised version of any required reporting checklist. It will be available to referees (and, potentially, statisticians) to aid in their evaluation if the manuscript goes back for peer review. A revised checklist is essential for re-review of the paper.

Please use the link below to submit your revised manuscript and related files:

[REDACTED]

Note: This URL links to your confidential home page and associated information about manuscripts you may have submitted, or that you are reviewing for us. If you wish to forward this email to co-authors, please delete the link to your homepage.

We hope to receive your revised manuscript within four to eight weeks. If you cannot send it within this time, please let us know. We will be happy to consider your revision so long as nothing similar has been accepted for publication at Nature Ecology & Evolution or published elsewhere.

Nature Ecology & Evolution is committed to improving transparency in authorship. As part of our efforts in this direction, we are now requesting that all authors identified as ‘corresponding author’ on published papers create and link their Open Researcher and Contributor Identifier (ORCID) with their account on the Manuscript Tracking System (MTS), prior to acceptance. ORCID helps the scientific community achieve unambiguous attribution of all scholarly contributions. You can create and link your ORCID from the home page of the MTS by clicking on ‘Modify my Springer Nature account’. For more information please visit please visit www.springernature.com/orcid.

Please do not hesitate to contact me if you have any questions or would like to discuss these revisions further.

We look forward to seeing the revised manuscript and thank you for the opportunity to review your work.

Yours sincerely,

[***REDACTED]***

Reviewer expertise:

Reviewer #1: polyploid evolution in Arabidopsis

Reviewer #2: Arabidopsis genetics and epigenomics

Reviewer #3: polyploid evolution

natureresearch

Reviewers' comments:

Reviewer #1 (Remarks to the Author):

The authors focused on the genomic and epigenomic analysis of a model allopolyploid species Arabidopsis suecica. The authors first conducted chromosome-scale assembly of natural and synthetic A. suecica. The latter was used as a substitute of a parental species A. arenosa, which I will discuss further below. Standard analyses of synteny and gene families as well as a focus on flowering and self-incompatibility genes follow. The authors did not find a major change that may be called genome shock. Then, the authors reported the main part of this manuscript, genome-wide DNA methylation analysis. Similar to previous studies using cotton, the methylation level of the two subgenomes became similar after hybridization, but in cotton low methylated subgenome became highly methylated in contrast to the decrease of high methylated subgenome in A. suecica. The authors identified differentially methylated regions.

The topic of epigenome of polyploid species is topical, and the dataset of the model allopolyploid A. suecica would be valuable. However, writing should be substantially improved. The methods and figure legends are often too short to follow. The correspondence between the main text and figures are often unclear.

A major issue that would need additional analysis is a circular argument about the genome conservation. The authors did not assemble directly the parental species A. arenosa, but the arenosa-derived subgenome of the synthetic A. suecica which experienced about 10 generations after allopolyploidization. Technically, it is a good idea to obtain homozygous state, because high heterozygosity of the autotetraploid A. arenosa would be a major barrier for genome assembly. The authors simply said in line 86 "Because of genome conservation, our further analysis considered the A and T subgenomes of resynthesized Allo738 to be A. arenosa (A) and A. thaliana (T, Ler) genomes, respectively." However, validation and careful interpretation would be necessary. It is not clear what aspect the authors say "genome conservation", or between which individuals the genomes were conserved. In other words, in this experimental setting, they cannot compare the genome before and after the hybridization (I mean, allopolyploidization). They were comparing the sequence right AFTER allopolyploidization and that after thousands of years.
In DNA sequences, it is no surprise if there were not be major changes in this laboratory 10 generations, if so, the genome may be conserved at the allopolyploidization event. However, there is no direct evidence shown in the manuscript. I would propose a few possibilities to check if the changes in the 10 generations were not huge, although the authors may find additional ways. First of all, at least, the Ler subgenome of synthetic A. suecica should be compared with the Ler genome. Fig. 1c showed a chromosome level synteny with Col accession of A. thaliana, but this is not adequate because the authors discussed smaller scales of changes in the text. There is a publication (Chromosome-level assembly of Arabidopsis thaliana Ler reveals the extent of translocation and inversion polymorphisms. Zapata et al. Proc Natl Acad Sci U S A. 2016 Jul 12;113(28):E4052-60) although I do not know if it is directly usable for your purpose. To compare arenosa subgenome with A. arenosa would be more important. Small amount of long-read data from natural A. arenosa (ideally close to the parent of the synthetic polyploid) may be adequate to validate the assembly.
Even when these validations are done, all texts including the abstract, results and discussion should be toned down when it comes to the interpretation of the conservation at the allopolyploidization.

In interpreting and discussing the results, the authors should consider seriously that variation within species cannot be examined with the analyzed samples. I do not mean that the authors should be increase the data, but the authors often consider the studied individuals as the representatives of a species. The manuscript did not discuss the distance between the individual of A. arenosa the author used to make the synthetic polyploid and the individual(s) that contributed to the origin of A. suecica 14,000-300,000 years ago. A. arenosa includes many subspecies and thus the distance can be fairly big. This difference would be particularly important for traits that are polymorphic within species, such as transposon insertion or small-scale rearrangement. The author did mention this general issue in studying polyploid species in the discussion line 357 "The species or strains used to form B. napus or wheat 8,000-10,00 years ago 60 may become extinct and different from the existing species." This is the same for their own data. The first example is found in line 95. "Interestingly, inversions and translocations occurred more frequently between A. arenosa and the A subgenome than between A. thaliana and the T subgenome of A. suecica (Fig. 1c; Extended Data Fig. 3a). This may suggest an increased rate of genetic diversity in the outcrossing A. arenosa or a different A. arenosa strain present in natural A. suecica."
However, another simple explanation is that the genotype of A. arenosa the authors used may be different from the very individual which contributed to the allopolyploidization 14,000-300,000 years ago. Thus the statement cannot be simply defended. Similarly, line 109 said " The Ks value distribution was higher between A. arenosa and the A subgenome than between A. thaliana and the T subgenome, suggesting a faster mutation rate in A. arenosa", but the issue is the same. If authors would like to discuss this point, population data of A. arenosa may give some insights, but it is difficult to exclude the possibility that unknown genotype existed previously, and so I would recommend to remove this conclusion. I do not think it is the reviewer's job to point out all similar issues throughout the manuscript, and I wish the authors would revise accordingly.

Abstract
In general, the abstract does not correspond to the content well.
L27 The sentence on results of self-incompatibility is not correct. The authors said "These epigenetic processes in the allotetraploids affect gene expression and phenotypic variation, including flowering, silencing of self-incompatibility". However, the result section described just the sequences of small RNA and its potential binding sites, which is nothing to do with "These epigenetic processes", which refer to DNA methylation in the previous sentence.

Results
L103, explanations other than homeologous exchanges seems also plausible. Please explain more if this conclusion is retained.

L117. The authors detected purifying selection, but no conclusions are drawn. There are already a few papers addressing the question whether purifying selection is weaker due to redundancy of homeologs in polyploid species (Capsella bursa-pastoris by Douglas et al. Proc Natl Acad Sci U S A. 2015 Mar 3;112(9):2806-11, A. kamchatica by Paape et al. Nat Commun. 2018 Sep 25;9(1):3909). These papers should be discussed in this context.

L118. Similarly, no reference was cited in the paragraph on the insertion time of transposable elements. There are studies in Arabidopsis species, and for example it is reported that recent insertion in A. thaliana was reduced associated with the transition to selfing (de la Chaux et al. Mob DNA. 2012 Feb 7;3(1):2). The conclusions should be discussed in the context of previous researches.

L127. The source of the A. halleri data was not found. Please add references. It may be Briskine et al. Mol Ecol Resour. 2017 Sep;17(5):1025-1036.

L132. The first sentence said similar, but the second sentence seems contradictory.

Fig. 2. A. lyrata and A. halleri are clustered and had zero common changes. Many phylogenetic studies including Novikova et al. Nat Genet. 2016 Sep;48(9):1077-82 showed that lyrata and arenosa cluster first, and then halleri comes outside. I hope then the data would make sense.

Fig. 2e. It is unclear which tissues were used for the analysis.

L155 Extended Data Fig. 5a,b do not seem relevant for the result of the sentence ("gain an exon from AaFLC1"). No figure legend explains a particular exon.

L174-184. The description and discussion on the self-incompatible genes should be revised thoroughly. First, the result in the line 178-180 "In A. suecica, the AaSCR allele is silenced by miR867 of SCR04 targeting the first exon of AaSCR with a frameshift mutation (Extended Data Fig. 6d)" was already reported by Novikova et al. (reference 15) and thus it should be cited here. There are indeed new results. It is unclear why "a long-term selection for selfing in the allotetraploid, leading to nonfunctional S-alleles" is suggested. With a single disruptive mutation in S genes, self-compatibility can evolve, and thus long-term selection is not relevant. More importantly, despite many studies, it has been difficult to detect the selection on self-compatibility from molecular evidences, and there is no molecular evidence in A. suecica supporting selection. A. suecica could have obtained self-compatibility at the origin, then selection was not relevant. Relevant review paper (for example, Shimizu and Tsuchimatsu, Ann Rev Ecol Evol Syst 46, 593-622, 2015) would provide further information. It is unclear what "gradual loss of self-incompatibility" in line 183 means. The sentence indicates no data or reference.

L194 and Fig.3a: it is not clear which row represents which individual, a second legend might be needed. There are five rows but the legend list six taxa. If a reader is very careful, one might notice that A. thaliana and A. arenosa are half and half in the same row, but still, it is not shown whether outside or inside correspond to them.

This figure is used to claim that the overall methylation levels were higher in A. arenosa than in A. thaliana, but this might only be true for CG methylation (which appears higher visually). For CHG and CHH methylation there might be an opposite trend, but all the judgement relies on visual inspection. This inspection might also be misleading because the average methylation level needs to be adjusted by the proportion of cytosines in each context, which is usually CHH >> CHG ~= CG. Genome size is also not taken into account. Methylation levels can be defined numerically, and one common approach is to calculate the global methylation level (see Vidalis et al. 2016).

L198-200, Fig. 3b, c and Extended Fig. 7d and e: there might be a conflict in the statement and the figures. The average methylation pattern in Fig. 3b shows A. suecica with lower average levels compared to all others. Visually one might assume that correlation between F1, Allo733, Allo738 and A/T should be much higher compared to A. suecica. We might come to similar conclusions based on the very close pattern observed in Extended Figures 7d and e too. Instead the correlation between F1, Allo733, Allo738 and A/T is very low, but it’s not clear why.

L198, "As a result" is not right. This is about the order of presentation, not about causal relationship.

L206: not clear how the epigenomic changes are “rapid and persistent” from the previous results. No difference between parents and synthetic polyploid are discussed here, so nothing can be rapid, and there is nothing persistent. This aspect should be addressed later when looking at DMRs and removed here.

L207 and L248. The definition of hypo and hyper DMRs are unclear, or the terminology is confusing.
Line 207 differentially methylated regions (DMRs) between the T or A subgenome in an allotetraploid and A. thaliana (T) or A. arenosa (A), respectively
Line 248 the DMRs between the subgenomes or A. arenosa (A) and A. thaliana (T)
Are they perhaps different or the same? The authors listed four genomes and connecting them with "and" "or", which made the sentences ambiguous.
After a long struggle, I suspect that line 207 means the difference between parent and polyploid (In Figure 3d, there are 4 categories, A hyper, A hypo, T hyper, T hypo.). In contrast I suspect line 248 means the difference between subgenomes. In line 248, then, it is a relative issue. Is hypo means higher in A subgenome or in T subgenome? Throughout the text, "higher methylation levels in T subgenome" or something equivalent should be used to clarify the meaning.
Regarding DMRs, the method must be much more detailed. It describes only one type of comparison (line 708). Is this consistent with the text?

L214: Extended Figure 8e: not present.

L219-237 and extended data fig. 9. The correspondence between the figure and the text is unclear. There seem two duplicated genes in A. arenosa, AaROS1-1 and AaROS1-2, in the figure, but the text describes only AsROS1. Please explain it. Seeing Extended Data Fig. 9a, only thaliana homeolog of ROS1 in A. suecica is upregulated, but the text did not distinguish homeologs and said "whereas ROS1 was expressed at the highest level in A suecica". These data do not support conclusions.

L235. "Predict" would be too strong, because there is only correlative evidence on ROS1 and methylation levels.

L249 and Fig. 4b. " (Fig. 4b). The number of hyper DMRs was reduced gradually from F1 to Allo733 and Allo738 and dramatically to natural A. suecica".
The way of figure presentation for this conclusion is deceiving. I do not think this conclusion is well supported. Allo733 and Allo738 are essentially biological replicates of the synthetic polyploid. It would be fair to show the two values on the same column. Then, the "gradual" pattern is not obvious with only 1 or 2 samples per class. To maintain the conclusion, please provide statistical support.

L263 and Fig. 4d. The legend does not explain what the dashed boxes in the figure are.

L269-270: how are “convergent” and “conserved” defined? What does the overlap mean? Is this from the line 241?

L313. "predict" is far too strong. All data are a kind of cherry picking and correlative.

Discussion

In this section the authors discuss DNA methylation in general terms, but most of the downstream analyses focus on CG methylation changes, meaning that most of the results refer to changes in CG context. This comes back to the comment of Fig. 3a, because not enough context is given to state that CG methylation changes represent the largest amount of changes in the genome. In addition, no DMR analysis was shown for the other two contexts. The global pattern suggests that the amount of DMRs might less, but numbers should confirm that. By better highlighting the importance and abundance of CG methylation changes, the reason behind continuing downstream analyses in CG context only would be more understandable and the discussion section would be better supported. For completeness, a short mention of the other two contexts could be considered as well.

Line 320 and 326. The term "ecological distribution" is unclear and unconventional. The reference papers do not seem to explain it. Then, in line 326, the authors discussed "despite diverse ecological distributions". The distribution range of A. suecica is rather narrow in the genus Arabidopsis.

Methods
Method are in general fairly brief. For RNA-seq and MethylC-seq data analysis, further details of mapping should be described. In allopolyploid species, a fragment may often be mapped to two homeologous regions with the same score, and their treatment may lead to errors (for example, Kuo et al. Brief Bioinform. 2020 Mar 23;21(2):395-407; Hu et al. Brief Bioinform. 2020 Mar 27:bbaa035). How were such reads treated?

L705-706: for reproducibility purposes, these Python scripts should be available. Also, how many cytosines were found to be conserved? This should be stated in the main text to better contextualize the amount of cytosines analyzed

L708-713: a sliding window approach has many limitations, but in the scope of this paper it makes more sense compared to other statistical approaches. One major limitation of sliding windows concerns multiple testing and power, which is something the authors do not seem to address in their methods where a threshold p-value is set, but no multiple testing correction is applied. In addition, the cut-off values of the methylation levels need to be clarified: were these values used as a minimum difference for testing
Extended Figure 8a and b: an upset plot might be better to show intersections and representing the size of the sets. An alternative would be to have the Venn diagram show circles proportional to the size. Figure b is really hard to understand, in particular what the asterisks refer to and what is the aim of the circle for all genes. There’s also no specification of how the overlap between DMRs and genes is defined: is a 1bp overlap enough to be associated to a gene?

Minor points
o L38-40: to rephrase. Polyploids do not generate genomic diversity (etc.) in response to selection, domestication or adaptation.
o L51: typo, "and"
o L144: typo in “allopolyploids”
o L191-192: not clear how that improves reproducibility.
o L233-235: Extended Fig. 7f and g show a slightly higher CHH methylation level in the tetraploids compared to A/T. The pattern is not as strong for Allo733, especially on the A-side where Allo733 has lower CHH levels.

o L246-247: Sentence should be less assertive.
o L270: typo in “pattern”
o L337: A. arenosa typo
o L358, 10,00 must be 10,000
o L386: A. lyrata typo.
o L684: add some details about sequencing platform and coverage.
o L694: add some details about sequencing platform and coverage.
o L700: any reason why the --score_min parameter was adjusted here?
o Legend Fig. 1, lyrate must be lyrata. translation must be translocation.
o Fig. 1c. The legend is too short to understand what the figure means.
o Extended Fig. 2a: axes unreadable.
o Extended Fig. 3d,e: why are the averages different.
o Extended Fig. 7: keep colors consistent
o Extended Fig. 9: ROS2 must be typo. ROS1-1 and ROS1-2?s Differentially expression must be differential expression.

Reviewer #2 (Remarks to the Author):

The authors of the manuscript by Jiang et al., “Concerted genomic and epigenomic changes stabilize Arabidopsis allopolyploids”, have generated high quality genomes for an allotetraploid species (A. suecica) and reconstituted equivalents. The latter were obtained by crossing the parental species believed to be the A. suecica ancestors, the naturally tetraploid A. arenosa (A genome) and a tetraploid version of the otherwise diploid A. thaliana (T genome). They describe that the genomes of synthetic and natural A suecica are highly similar, synthenic, and colinear, confirming the assumed ancestry as well as the genome quality. They also describe interesting differences between the frequency of polymorphism types between the A and the T genome. While most gene families are shared between A and T, lineage-specific genes include genes connected with outcrossing (A, plausible) or less explained other GO terms in T. Further analyses address individual genes (FLC) or functional group of genes (self incompatibility). A large part of the work describes the status of CG/CHG/CHH DNA methylation in genic or TE context and allows to conclude a gradual convergence of the methylation status in the evolution of alloploids, with the exception of maintaining differences at specific differentially methylated regions associated with genes of some functional groups. Finally, the authors can link different degrees of methylation in their material with different expression of genes related to reproduction.
The potential to compare the natural with the resynthesized allotetraploid species Arabidopsis suecica has been and still is a rewarding model to learn what happens upon the combinations of similar but different genomes after the formation of alloploids. The inclusion of two independent synthesized allotetraploids as well as a “fresh” F1 in the experimental design is appreciated. Although a lot of literature, including contributions from the same lab, addressed similar questions before, a detailed analysis was so far hampered by the lack of good reference genomes for the arenosa subgenome. The genomic information obtained from well-chosen material provided here is certainly a valuable resource and allows most of the conclusions drawn here. That said, most of the statements are not surprising: this is positive, as the data are congruent with partial data known before, or they are plausible by expectation (e.g. outcrossing), while it limits a bit the excitement to find unexpected new insight. Naturally, and not meant critically, the data are largely of descriptive nature. This provides a rich resource for future work that can address the role of specific pathways or functional groups of genes.

However, here, only the potential for such work is visible, and the brief mentioning of examples for genes for which the methylation differences and their dynamic changes might be relevant (p. 16, SMC3, PDS5A, AFB3) leaves the reviewer rather unsatisfied, especially as equally relevant genes (other SMCs or F-box factors) are not compared as “control group”. The Discussion could be freed from some redundance with the Introduction, but it is appreciated that the authors discuss the limited comparability of their system with the conditions for other allopolyploids that were likely formed at different times and between parents of larger, more diverse genomes.
A few minor points could make the manuscript also stronger: .
Some of the analyses involve data from A. lyrata and A. halleri, but the Introduction fails to introduce the connection of these species with the other plant material. This information should be added.
Copy number and structural variants of FLC are interesting to compare, but the DNA methylation analyses (Fig. 2e), in the absence of parallel chromatin and lncRNA analysis, is more irritating than clarifying and should be deleted.
Some proofreading is recommended (e.g. line 51).

Reviewer #3 (Remarks to the Author):

Please find the bibliographic references used for this report at the end of it.

Key Results

In this work, the authors deliver the genomic sequences and the epigenetic landscape of Arabidopsis arenosa and the two constitutve subgenomes of the allotetraploid A. suecica (A. thaliana and A. arenosa). Jiang and col. show how the constitutive genomes of A. suecica have remained stable after polyploidization, without drastic rearrangements, contrasting the genome shock that reported for other known allopolyploids. Nevertheless, some subtle changes are reported to differentially affect the two constitutive genomes (i.e.: gene family contraction/expansion and copy number variation). More remarkably, despite the higher initial levels of DNA methylation in one of the subgenomes in newly resynthetised allotetraploids, it is reduced during the first generations after allopolyploidization, to finally converge with the less methylated subgenome in the natural A. suecica. Finally, the authors identified some genes affected by the mentioned epigenetic changes as candidates to impact the reproductive performance during the stabilization of Arabidopsis allopolyploids.

Validity

In general, I am quite positive about the validity of these results and their interpretation. However, I do find some issues that should be addressed.

The genome data provided looks reliable given the quality parameters reported, and the validation of the assemblies performed. Moreover, the synteny of A. arenosa genomes with A. lyrate helps to evaluate comparatively the quality of the assembly. Regarding the strategy to sequence the A. arenosa genome, so long time hampered by its heterozygosity, I consider that introducing this genome in A. suecica, which is able to self-polinate, to get rid of the heterozygosity has some risks. This is because the possibility of having homoeologous exchanges (HEs) between subgenomes, which has been reported to happen in synthetic suecica (Henry et al., 2014). Though the chances some HEs could have got fixed in 10 generations of selfing are not negligible, it might not affect the results

and/or the conclusions of this work. It should be relatively simple to control this possibility, by performing coverage analyses (Henry et al., 2014). I think this could be especially relevant for the conclusions on expansion and contraction of gene families. Having this concern out of the way, I think that the validity of the genome quality is extensive to the downstream analyses performed.
Apart from that, there is an additional issue that I think it will be worth to address. I am sure the authors are aware about the preprint available in biorxiv (Burns et al 2020) on the A. suecica genome. Though, in fact, both papers, are quite complementary and address different biological questions they do overlap in one of them that happens to have apparently contradictive results: the TEs. While Jiang et al present very similar levels of TE in all the genomes analyzed (Exteded data fig 1a), Burns et al report that the A. arenosa genome from A. suecica have twice as TEs than the A. thaliana genomes. I wonder if the authors could address this apparent contradiction or propose any explanation for it (e.g. different A. suecica accession, different quality of the assemblies, etc).
Regarding the results on the epigenomic changes, I find them quite robust as they show a very nicely consistent trend from F1 to F10 to natural A. suecica. This holds true when data is assessed in different ways (i.e. in chromosome scale, as gene body and flanking sequence or when DMR analyses are performed). Moreover, in case of any interference created by HEs, I do not think it will affect the clear trend and consistency shown by the data. Though this is not the first time the epigenomic landscape is described in an allopolyploid species, the originality of this work lays on addressing the interplay of this feature with the process of stabilization that is obligatory for the survival of polyploids. How this stability is achieved is of great interest for the evolutionary point of view, but also for agriculture given the importance of polyploids.

My only validity issue would be with the tittle as “Concerted genomic and epigenomic changes stabilize Arabidopsis allopolyploids”, suggest evidence that causally links the stability of A. suecica to the epigenomic and genomic changes described. In my opinion, this evidence would be very complex to obtain as it might not be technically possible to verify if the stability of A. suecica would be compromised when the genomic and epigenomic changes do not happen. Alternatively, the authors report on a set of genomic and epigenomic features than can reasonably be hypothesized to be associated with the stability of A. suecica, but do no demonstrate, in my opinion, the causality expressed by the tittle. My recommendation would be to change the tittle to something like: “Concerted genomic and epigenomic changes accompanies stabilization of Arabidopsis allopolyploids” or, “Epigenomic convergence accompanies genomic stability during Arabidopsis allopolyploids formation” or something similar. I think it would be more accurate.

Other than these points, I also have some minor comments on the validity of particular statements that I have exposed in the section for Suggested Improvements of this report.

Significance

In my opinion, one of the pieces of added value delivered by this work is a long time awaited high-quality assembly of A. arenosa genome. This model species has enabled important work in the last two decades without having a good reference sequence. A. arenosa forms well-established auto- and allopolyploids so I would not be surprised if this work boosts the relevance and utility of this model in the study of polyploidy evolution.

In addition, this study illustrates very well the methylation landscape changes through the polyploidization process, impacting expression and generating variation that can be the raw material for adaptation. The data presented suggests that an important part of the epigenomic changes that

might contribute to stabilize allopolyploids emerges as a mere consequence of polyploidy while another part is remodelled after a few generations. This rises new interesting biological questions about the role of natural selection in this process that might inspire future research

Moreover, this study identifies some genes that could be potential candidates that could inspire future reverse genetics experiments to further characterize their role in polyploid stability, in Arabidopsis but also in crops. Some of these genes make a lot of sense in the light of the literature (e.g. effect of PDS5 reported by Bian et al, 2018).

Data and methodology

The procedures to assembly and annotate the genome are sufficiently described as well. Moreover, I find the analyses performed in this work appropriate to describe and quantify the genome structure and DNA methylation landscape of A. suecica and A. arenosa. Moreover the presentation of the data in very informative and visual graphs facilitates the reading. I didn't miss any piece of data within the provided files.

Analytical approach

In general, I found the analytical approach appropriate and the sample sizes are sufficient (3 biological replicates). I did miss some statistical test for some specific pieces of data but I have mentioned those cases in mi minor comments for the Suggested improvements part.

Suggested improvements

Major suggestions

All my major comments are manifested below in the corresponding sections of this report accordingly to the aspect involved.

Minor suggestions

Here, some suggestions that, in my opinion, might improve the paper:
-In the abstract (line18), it is mentioned that there are "concerted genomic and epigenomic diversifications in resynthesized and natural a. suecica". Personally, I find this sentence a bit confusing (considering that is the second sentence that the reader will read while approaching the paper. They my wonder: does it mean that resynthesized and the natural become different? or is it that their genomes -become different? Moreover, I also find the concept of "concerted diversification" not entirely clear. In the next lines (any case, I think that if it means that the A and T genomes become different in some aspects, two elements (A and T) are not enough to speak about diversity (diversification means diversity is enhanced).
-In my opinion, the word "diversifications" when the differences identified between the A and T genomes are referred to is not accurate, as two elements (A and T) are not enough to speak about diversity (as diversification suggests that diversity is enhanced). For instance, the word diversification is very appropriate to speak about the multiple species of Gossypium. However in this case...How about using the word "variation".
-The abstract mentions the concept of inter-genomic incompatibility which is not further elaborated as a problem for the stability of polyploids (lines 31 and 32).

-Though it is true that autopolyploid bananas exist, the allopolyploid varieties are the ones that prevail in the literature. So, if I had to place bananas only in one cathegory, it would not be in the autopolyploid box without using the word “some”. Line 36.
-I think it would improve the clarity and accuracy of figure1a if it includes the information (already stated in the main text) that both the parents and the F1 as well were already tetraploid.
-The table 1 shows the size of the assembled genome. I believe it would be interesting to contrast this number with DNA contents estimated by flow cytometry.
-As it has been proposed that the genome shock accompanies whole genome duplication often involves a boost in the frequency of transposable elements, I find interesting that there are not apparent differences in TE frequencies as shown in supplementary figure 1 of the extended data. I would suggest to mention this in the text and also, if possible, to verify this statistically. Moreover, I would also suggest to discuss this result in the light of the observations from Baduel et al 2019, which reported a boost in A. arenosa autopolyploids. Does this mean that the TE levels of thaliana are higher than the levels of diploid Arenosa, but comparable to tetraploid arenosa?.
-For the figure 1C. Personally, I like when the color key is shown in the figure and which is more direct than going to the text.
-Line 99-100. I would say “more collinear regions” rather than “higher collinear regions”. Moreover, here a statistical test might be missing here.
-Lines 102-106. From the figure I understand that the T genome shows higher SNP density in translocations between subgenomes, but I find the text a bit difficult to understand this. Likewise it is not very clear for me whether those SNPs are between subgenomes or between progenitor and natural suecica.
-Lines 110-111. In my opinion, the higher Ks between A and sA is more likely one more evidence (along with the higher frequency or rearrangements detected) that the actual donor of A-subgenome is less similar to the A-progenitors than the T-progenitor used in this study. This interpretation is more in agreement with the observations described in lines 111-112: constant rate of evolution of subgenomes.
-The legend of the Extended data Figure 3a mentions s values sA vs sA. I think that the authors actually meant sA vs A.
-In my opinion, a higher Ka does not necessarily mean faster evolution, as suggested in line lines 114-116 and extended Data Fig 3a, as it could mean simply greater distance or higher mutation rate. I think that a higher Ks/Ks, by contrast, would be indicative of faster evolution and it doesn’t seem to be the case here.
-Lines 116-117. Maybe some statistics would be required here, though no obvious differences can be seen.
-Lines 120-122 and figure 1f. It is suggested that polyploidy is responsible for the (on average) younger insertion time of TEs in T subgenome of A. suecica than in the progenitor. I think it would be good to provide the sample size but, in any case, it seems that most of the data contributing to the distribution is for insertion times of more than 0.3 MYA. It is not obvious for me how polyploidy can explain the differences here. I would suggest further clarification.
-In figure 2b, I think a threshold (for significance or showing the average fold enrichment) could help the reader to understand how much the fold enrichment for those particular GO stands out.
-For figure 2c, I found the blue color a bit difficult to distinguish, maybe I would recommend to use a different tone of blue. Moreover, as I mentioned, I think that adding the color code directly on the figure (rather than in the legend) would make it more comfortable to visualize.
-Lines 155-156. Here, I find a bit difficult to suggest that reproduction genes can be (especially) fast in experiencing homoeologous exchanges. A functional bias (towards reproduction genes) would likely imply selective pressures which are difficult to assume in ten generations of lab conditions. I would

just say that this informs on how fast homoeologous exchanges can happen. However, I find very intriguing the fact of having only one exon introduced which can only be explained with two events of HEs in a very narrow window of space and time. I wonder if the authors have checked if it also presents in plant 733?
-In the lines 159-160, there is a double negation that is a bit confusinng “The flowering time variation was consistent with negative correlation of higher FLC expression with lower DNA methylation levels”. I would just remove the “negative correlation”.
-Regarding the correlations between FLC expression and methylation, though the figure 2e is nice, maybe I miss some more more quantitative information. How about showing the expression in form of FPM with the corresponding statistics?
-I miss a reference in the text to the siRNA levels shown in figure 2e. Maybe some further explanation would be nice.
-I don’t understand (lines 173-174) why the presence of polymorphisms between A. suecica and other species suggest that there is a long term selection for selfing. Is it not that other obligate utcrossing species also have polymorphisms?
-Unless I am missing any data or previous literature, I would clarify that the silencing of the A-copy of SCR by the miRNA from the T-copy is just a prediction. I guess that the RNA- and Methyl-seq data come from tissues where the S-locus is not expressed but does it provide any information by any chance?
-The dominance hierarchy of the S-locus is an idea (line 181) that has not been introduced and some readers might not be totally aware of it.
-It is not sufficiently clear in the text (line 190) which A. thaliana plants were used to measure methylation levels, (diploid, tetraploid line, Ler, col etc..).
-In the legend of the extended Data Fig7c. Would not it be clearer to say that it is a heatmap of pairwise comparisons of natural A. suecica, with arenosa, thaliana and F1, 733, 738.
-Suddenly, the Allo733 is introduced in line 190 part without much information about it and the reader can just assume that is a similar to 738.
-Regarding the comparison of expression levels in lines 224-226 and Extended Data fig 9b and 9c, again, I believe showing expression in FPM and some statistics would be nice.
-In extended data Fig10a. I would also suggest to include a straight line with any of threshold (significance, genome-wide average or something like that).
-Lines 310-311. For the sake of accuracy, the cited study in A. arenosa autopolyploids what actually shows is that PDS5
- Lines 332-333. Again I am not entirely sure if the target prediction of one miRNA is sufficient to confirm the model proposed in this paper for the S-locus overcoming.
-Lines 635 636 Jukes-cantor to stimate LTR insertion age. Maybe I would include a reference for this method.
-The sequencing approaches used are appropriate for DNA and mRNA, but I maybe I missed one sentence on which technique was used for Methyl-seq. I know that a reference is provided in line 696, but I think it will not harm just to mention bisulfite sequencing.

If the authors want to expand the scope of the study, here are three suggestions:
1. Normally, it is assumed that natural selection plays a very important role in the stabilization of polyploids. This data suggests that some of the genes potentially involved in the stability of allopolyploids undergo epigenetic changes from the F1 itself (conserved DMR). I wonder if the authors could include some ideas on the discussion on the role of natural selection for the stability of allopolyploids (see Significance part of this report). Another way to approach this question could be to perform some analyses of the outliers of the Ka/Ks distribution regarding the epigenetic changes. Are

the genes with the strongest or weakest purifying selection enriched in DMR? Is this enrichment conserved from F1, or associeated to final convergence in natural A. suecica? I think this might show a beautiful interplay between selection and methylation.
2. I am curious if there is any correlation between genes that rapidly tend to get hypermethylated (presumably silenced) and the ones that end up getting lost (family contraction). Have the authors considered including these analyses? It could be interesting to look for some meiotic genes that are known for their rapid return to single copy in paleopoliploids (De Smet et al 2013, Lloyd et al 2014).
3. I am sure that the authors have already considered this, but I wonder if it has been given any attention to THE BOY NAMED SUE locus described to impact allopolyploid stability (Henry et al 2014). E.g. methylation changes of the locus, candidate genes.

Clarity and accessibility

I have two major comments regarding the clarity of the paper:

1. Personally, I find the nomenclature of genomes sometimes inconsistent or unclear. I feel that depending on which part of the results we are the same subgenomes is called in different forms. I know that it is difficult, but I would suggest to stick to the same nomenclature during the entire paper. Otherwise, the reader might doubt if you mean the subgenome or the progenitor species, the F1, the synthetic or the natural one. I have the feeling that these inconsistences happen several times during the text. Here some examples that were confusing for me during my reading:
• Abstract lines 21-22. Is this meaning about Arenosa progenitor? If so, I would use the word “progenitor” to avoid ambiguity.
• Lines 91 and 92 though the figures are clear about it. I think that the text should explicitly say that the high levels of coliniarity for sA and sT are in comparison with the progenitor genomes (A and T). Otherwise it can lead to other interpretations
• Extended data figure 2: the 738 and As is used again.
• In the figures, A and T refes to the genomes from 738 and the sA and sT from the natural A. suecica. However, in the text, A and T are often referred to as the genomes from the natural A. suecica (e.g. lines 109-112).
• In the figure 1f. What is the difference here between T and T(738)? I understand that T Ler is from the reference of diploid Ler…
• Lines 86 and 87: it is stated that the A and T subgenomes fo resynthesized will be referred to as A. arenosa (A) and A. thaliana (T, Ler). I feel it would help to state clearly that the A and T genomes from Allo738 will be there after considered as the reference for A. arenosa and A. thaliana progenitors, respectively.
• In line 190, it seems that all the abbreviations are again re-defined and though, I eventually, assumed that the methylome of A arenosa was obtained from autotetraploid A. arenosa (and not from Allo 733) it was not very intuitive to me. Moreover, it is not clear what material was used for A. thaliana, is it diploid or autotetraploid Ler? Or, is it Col?
• Moreover, the combination of all these denominations with the use of sA (for arenosa genome within suecica) and As (for A. suecicica in general) used in some figures (e.g. figure 3) adds more confusion, in my opinion.
• When the Methyl-seq results are introduced (line 190) and then, the nomenclature changes again: and T and A do no longer represent the data coming from Allo738 but from the actual thaliana and arenosa (autopolyploids? Not clarified).
In summary, I think that choosing one nomenclature and stick to it will significantly improve the reading experience and the clarity of the results. I let the authors decide which is the best system, on

option might be to use full names names: A. arenosa, A. thaliana, A. suecica A, and A, suecica T. Another option could be to do something similar to the system for Brassicas (sub)genomes: Aar.A, Ath.T, Asu.A, and Asu.T combined with full names when no subgenomes is specified. Irrespective of the nomenclature chosen, it would be important to stick to it as much as possible. I know that it is slightly complex to explain that the genome reference of A. arenosa and A. thaliana actually come from Allo 738 while the methyl-seq data doesn't, but I think it can be explained.

2. Personally, I like when every results section finishes with one sentence summarizing the main findings. Especially when a lot of data is provided. In my opinion, this is better than finishing with an interpretation or prediction based on the data that likely fits better in the discussion. I am totally fine with using the Results section to mention that an observation makes sense in the light of the literature (e.g. lines 142-145), but I do prefer to keep situations like proposing hypothesis (lines 155 to 156) or making predictions (e.g. lines 235 to 237 or 313 to 314) based on data for the discussion. I would suggest to slightly remodel the results part accordingly to make a more comprehensive discussion.

Other than these two issues, I feel that the paper was very accessible and the figures very illustrative, contributing to a smooth reading experience. I have some other minor suggestions to improve the clarity of some concrete parts that I have already manifested in the section of Suggested Improvements section of this report.

References
In my opinion, two minor changes regarding the references of this paper might provide a major improvement in the clarity and the strength of the paper:

-I think that if the introduction should state that the literature suggests, as the most likely origin, that autotetaploid (and not diploid) arenosa was the donor of the A genome of A. suecica. Otherwise, this could confuse the reader. For instance, someone criticism might arise from doubting whether the high methylation levels observed in synthetic allopolyploids were just a product of tens of thousands years of autopolyploidy and if the low levels of methylation in natural suecica were just a consequence of the diploid origin of its true parents. I believe that clarifying the origin of natural A. suecica in the introduction to would prevent this potential misconception, thus validating the plant materials and the approach used and the conclusions.

-I feel that it will further strengthen the validity and the relevance of the results if Bian et al., 2017 is cited while discussing the implications of downregulation of PDS5. In this work the authors artificially reduced the expression of this gene (using VIGS) in allohexaploid wheat, which resulted in meiotic instability. I think that this provides a good validation for the results of this paper.

Bibliography
Burns et al. Gradual evolution of allopolyploidy in Arabidopsis suecica. Biorxiv. 2020. https://www.biorxiv.org/content/10.1101/2020.08.24.264432v1
Baduel et al. Relaxed purifying selection in autopolyploids drives transposable element over-accumulation which provides variants for local adaptation. 2019. Nat. Comm.
Bian et al. Meiotic chromosome stability of a newly formed allohexaploid wheat is facilitated by selection under abiotic stress as a spandrel. New Phytologist, 2018. https://doi.org/10.1111/nph.15267
Henry et al. The BOY NAMED SUE quantitative trait locus confers increased meiotic stability to an adapted natural allopolyploid of Arabidopsis. Plant Cell. 2014.10.1105/tpc.113.120626

De Smet et al. Convergent gene loss following gene and genome duplications creates single-copy families in flowering plants. 2013 PNAS. 10.1073/pnas.1300127110
De Smet et al. Meiotic gene evolution: Can you teach a new dog new tricks? 2014 Mol. Biol. Evol. 10.1093/molbev/msu119

********************END********************

Author Rebuttal to Initial comments

Reviewer expertise:

Reviewer #1: polyploid evolution in Arabidopsis

Reviewer #2: Arabidopsis genetics and epigenomics

Reviewer #3: polyploid evolution

Reviewers' comments:

Reviewer #1 (Remarks to the Author):

The authors focused on the genomic and epigenomic analysis of a model allopolyploid species Arabidopsis suecica. The authors first conducted chromosome-scale assembly of natural and synthetic A. suecica. The latter was used as a substitute of a parental species A. arenosa, which I will discuss further below. Standard analyses of synteny and gene families as well as a focus on flowering and self-incompatibility genes follow. The authors did not find a major change that may be called genome shock. Then, the authors reported the main part of this manuscript, genome-wide DNA methylation analysis. Similar to previous studies using cotton, the methylation level of the two subgenomes became similar after hybridization, but in cotton low methylated subgenome became highly methylated in contrast to the decrease of high methylated subgenome in A. suecica. The authors identified differentially methylated regions.

The topic of epigenome of polyploid species is topical, and the dataset of the model allopolyploid A. suecica would be valuable. However, writing should be substantially improved. The methods and figure legends are often too short to follow. The correspondence between the main text and figures are often unclear.

Response: We appreciate the encouraging and constructive analysis of our work from this expert reviewer and have addressed the concerns in this revision.

A major issue that would need additional analysis is a circular argument about the genome conservation. The authors did not assemble directly the parental species A. arenosa, but the arenosa-derived subgenome of the synthetic A. suecica which experienced about 10 generations after allopolyploidization. Technically, it is a good idea to obtain homozygous state, because high heterozygosity of the autotetraploid A. arenosa would be a major barrier for genome assembly. The authors simply said in line 86 "Because of genome conservation, our further analysis considered the A and T subgenomes of resynthesized Allo738 to be A. arenosa (A) and A. thaliana (T, Ler) genomes, respectively." However, validation and careful interpretation would be necessary. It is not clear what aspect the authors say "genome conservation", or

between which individuals the genomes were conserved. In other words, in this experimental setting, they cannot compare the genome before and after the hybridization (I mean, allopolyploidization). They were comparing the sequence right after allopolyploidization and that after thousands of years.
In DNA sequences, it is no surprise if there were not be major changes in this laboratory 10 generations, if so, the genome may be conserved at the allopolyploidization event. However, there is no direct evidence shown in the manuscript. I would propose a few possibilities to check if the changes in the 10 generations were not huge, although the authors may find additional ways. First of all, at least, the Ler subgenome of synthetic A. suecica should be compared with the Ler genome. Fig. 1c showed a chromosome level synteny with Col accession of A. thaliana, but this is not adequate because the authors discussed smaller scales of changes in the text. There is a publication (Chromosome-level assembly of Arabidopsis thaliana Ler reveals the extent of translocation and inversion polymorphisms. Zapata et al. Proc Natl Acad Sci U S A. 2016 Jul 12;113(28):E4052-60) although I do not know if it is directly usable for your purpose. To compare arenosa subgenome with A. arenosa would be more important. Small amount of long-read data from natural A. arenosa (ideally close to the parent of the synthetic polyploid) may be adequate to validate the assembly.
Even when these validations are done, all texts including the abstract, results and discussion should be toned down when it comes to the interpretation of the conservation at the allopolyploidization.

Response: These are valid comments. We thank Dr. Magnus Nordborg for sharing A. arenosa sequence (bioRxiv, Burns et al. 2020). "To test stability of resynthesized *Arabidopsis* allotetraploid Allo738, we compared the genome of Allo738 with L*er* (Zapata et al., 2016) and other *Arabidopsis* species (Novikova et al., 2016; Novikova et al., 2017) including an *A. arenosa* accession (https://doi.org/10.1101/2020.08.24.264432), and Allo733 (a sibling of 738) (Extended Data Fig. 3). We found that (1) Allo733 and Allo738 have similar levels of divergence to L*er* and Aar4, respectively (Extended Data Fig. 3a); (2) A subgenomes of Allo733 and Allo738 are closely related to *A. arenosa* accessions that are in a different clade from A subgenomes of *A. suecica* accessions (Extended Data Fig. 3b); and (3) T subgenome of Allo733 and Allo738 are closely related to L*er*, which is different from T subgenome of *A. suecica* accessions (Extended Data Fig. 3c). Neighbor-joining evolutionary tree also indicated that the A-subgenome donor of Allo733 and Allo738 was closest to *A. arenosa* (Novikova et al., 2017) (Extended Data Fig. 3b), and T-subgenome donor of Asu was closely related to ecotypes from Russia of Asia admixture of 1135 strains analyzed (Extended Data Fig. 3c) (Consortium, 2016; Novikova et al., 2016). These analyses confirm that A and T subgenomes of resynthesized Allo738 (and Allo733) can be treated as *A. arenosa* (Aar) and *A. thaliana* (Ath, L*er*) genomes, respectively, for further analysis." We also updated source data with these new analyses.

In interpreting and discussing the results, the authors should consider seriously that variation

within species cannot be examined with the analyzed samples. I do not mean that the authors should be increase the data, but the authors often consider the studied individuals as the representatives of a species. The manuscript did not discuss the distance between the individual of A. arenosa the author used to make the synthetic polyploid and the individual(s) that contributed to the origin of A. suecica 14,000-300,000 years ago. A. arenosa includes many subspecies and thus the distance can be fairly big. This difference would be particularly important for traits that are polymorphic within species, such as transposon insertion or small-scale rearrangement. The author did mention this general issue in studying polyploid species in the discussion line 357 "The species or strains used to form B. napus or wheat 8,000-10,00 years ago 60 may become extinct and different from the existing
species." This is the same for their own data. The first example is found in line 95. "Interestingly, inversions and translocations occurred more frequently between A. arenosa and the A subgenome than between A. thaliana and the T subgenome of A. suecica (Fig. 1c; Extended Data Fig. 3a). This may suggest an increased rate of genetic diversity in the outcrossing A. arenosa or a different A. arenosa strain present in natural A. suecica."
However, another simple explanation is that the genotype of A. arenosa the authors used may be different from the very individual which contributed to the allopolyploidization 14,000-300,000 years ago. Thus the statement cannot be simply defended. Similarly, line 109 said " The Ks value distribution was higher between A. arenosa and the A subgenome than between A. thaliana and the T subgenome, suggesting a faster mutation rate in A. arenosa", but the issue is the same. If authors would like to discuss this point, population data of A. arenosa may give some insights, but it is difficult to exclude the possibility that unknown genotype existed previously, and so I would recommend to remove this conclusion. I do not think it is the reviewer's job to point out all similar issues throughout the manuscript, and I wish the authors would revise accordingly.

Response: We appreciate this comment on data interpretation. As noted by the reviewer, we did not intend to study diversity within progenitor species and presented alternative possibilities. For line 95, we did include this alternative notion (see below). For *Ks* value analysis, we revised, "The *K*s value distribution was higher between *A. arenosa* and the A subgenome than between *A. thaliana* and the T subgenome, which is consistent with more structural variation observed in A than T subgenome." We added, "This may suggest an increased rate of genetic diversity in the outcrossing *A. arenosa* or a different *A. arenosa* strain involved in the formation of natural *A. suecica*." From the published *A. arenosa* resequencing data (Burns et al. 2020, *biorxiv*), we found that the *A. areonsa* (Aar4) is indeed relatively close to the A genome donor of *A. suecica*. This conclusion is also consistent with the phylogenetic data previously reported (Novikova et al., 2016) (Extended Data Fig. 3). We have checked and toned down other relevant statements.

Abstract
In general, the abstract does not correspond to the content well.
L27 The sentence on results of self-incompatibility is not correct. The authors said "These

epigenetic processes in the allotetraploids affect gene expression and phenotypic variation, including flowering, silencing of self-incompatibility". However, the result section described just the sequences of small RNA and its potential binding sites, which is nothing to do with "These epigenetic processes", which refer to DNA methylation in the previous sentence.

Response: As suggested, we revised the sentence. "These epigenetic processes including small RNAs in the allotetraploids may affect gene expression and phenotypic variation, including flowering, silencing of self-incompatibility, and upregulation of meiosis- and mitosis-related genes."

Results
L103, explanations other than homeologous exchanges seems also plausible. Please explain more if this conclusion is retained.

Response: This could be a confusion. It was meant, as expected, that the SNP frequency in the T segment translocated to A subgenome is low, and the SNP frequency in the A segment translocated to in T subgenome is high. This suggests stable maintenance of high SNP frequency in the A segment and low SNP frequency in the T segment of these exchanged regions in allotetraploids. We clarified this in the revision.

L117. The authors detected purifying selection, but no conclusions are drawn. There are already a few papers addressing the question whether purifying selection is weaker due to redundancy of homeologs in polyploid species (Capsella bursa-pastoris by Douglas et al. Proc Natl Acad Sci U S A. 2015 Mar 3;112(9):2806-11, A. kamchatica by Paape et al. Nat Commun. 2018 Sep 25;9(1):3909). These papers should be discussed in this context.

Response: That's a valid comment. As suggested, we added a sentence to clarify this. "However, purifying selection is generally weaker due to redundancy of homoeologs in allopolyploids as reported in *A. kamchatica* (Douglas et al., 2015) and *Capsella bursa* (Paape et al., 2018), and allopolyploidy might have weakened natural selection because of this bottleneck effect."

L118. Similarly, no reference was cited in the paragraph on the insertion time of transposable elements. There are studies in Arabidopsis species, and for example it is reported that recent insertion in A. thaliana was reduced associated with the transition to selfing (de la Chaux et al. Mob DNA. 2012 Feb 7;3(1):2). The conclusions should be discussed in the context of previous researches.

Response: As suggested, we discussed the reduced insertion of LTR after selfing in *A. thaliana*. We revised to "The order of insertion time is *A. thaliana* > *A. lyrata* > *A. arenosa*. (Fig. 1f), which seems to correlate with different mating systems, as recent insertions in *A.*

***thaliana* were reduced from the transition of outcrossing in *A. lyrata* to selfing (de la Chaux et al., 2012)."**

L127. The source of the A. halleri data was not found. Please add references. It may be Briskine et al. Mol Ecol Resour. 2017 Sep;17(5):1025-1036.

Response: As suggested, we cited the reference for *A. halleri* along with *A. lyrata* and *A. kamchatica* in the Introduction.

L132. The first sentence said similar, but the second sentence seems contradictory.

Response: We revised, "Analysis of the gene family contraction and expansion revealed uneven rates of gain or loss among allopolyploid species examined (Fig. 2c)."

Fig. 2. A. lyrata and A. halleri are clustered and had zero common changes. Many phylogenetic studies including Novikova et al. Nat Genet. 2016 Sep;48(9):1077-82 showed that lyrata and arenosa cluster first, and then halleri comes outside. I hope then the data would make sense.

Response: This is a good comment. We have revisited the analysis of trees, and the result remained unchanged. This could be due to closeness of *A. arenosa* to *A. suecica* and a small number of species used in this study. We stated, "Note that clustering between *A. lyrata* and *A. halleri* could result from a small number of species used in the study, while *A. lyrata* and *A. arenosa* may be more closely related (Novikova et al., 2016)." However, this discrepancy does not affect interpretation of the data.

Fig. 2e. It is unclear which tissues were used for the analysis.

Response: Rosette leaves before bolting, 3-4 weeks for *A. thaliana* and 6-7 weeks for *A. arenosa*, F_1, Allo733, Allo738, and *A. suecica*, as previously reported in several studies (Wang et al., 2006a; Wang et al., 2006b; Ni et al., 2009; Shi et al., 2012; Shi et al., 2015) to standardize the stage of "prior to bolting."

L155 Extended Data Fig. 5a,b do not seem relevant for the result of the sentence ("gain an exon from AaFLC1"). No figure legend explains a particular exon.

Response: The figure shows gene structure of the longest transcript. We removed the sentence, which is a previously reported result (Nah and Jeffrey Chen, 2010).

L174-184. The description and discussion on the self-incompatible genes should be revised thoroughly. First, the result in the line 178-180 "In A. suecica, the AaSCR allele is silenced by miR867 of SCR04 targeting the first exon of AaSCR with a frameshift mutation (Extended Data

Fig. 6d)" was already reported by Novikova et al. (reference 15) and thus it should be cited here. There are indeed new results. It is unclear why "a long-term selection for selfing in the allotetraploid, leading to nonfunctional S-alleles" is suggested. With a single disruptive mutation in S genes, self-compatibility can evolve, and thus long-term selection is not relevant. More importantly, despite many studies, it has been difficult to detect the selection on self-compatibility from molecular evidences, and there is no molecular evidence in A. suecica supporting selection. A. suecica could have obtained self-compatibility at the origin, then selection was not relevant. Relevant review paper (for example, Shimizu and Tsuchimatsu, Ann Rev Ecol Evol Syst 46, 593-622, 2015) would provide further information. It is unclear what "gradual loss of self-incompatibility" in line 183 means. The sentence indicates no data or reference.

Response: These are valid comments. We removed the statement of long-term selection and gradual loss. Our intention was to discriminate the early stages of self-incompatibility in Allo733 and Allo738 (1-5 generations) and sequence variation between natural *A. suecica* and *A. kamchatica.* We added reference 15 in the line and clarified these results in the Results.

L194 and Fig.3a: it is not clear which row represents which individual, a second legend might be needed. There are five rows but the legend list six taxa. If a reader is very careful, one might notice that A. thaliana and A. arenosa are half and half in the same row, but still, it is not shown whether outside or inside correspond to them.

Response: As suggested, we revised the Fig 3a to "a, Chromosome features and methylation distributions. Notes in circos plots: (1) chromosomes, (2) gene and (3) TE density, and (4) CG, (5) CHG and (6) CHH methylation levels using 100-kb windows in *A. thaliana* or *A. arenosa*, F_1, Allo733, Allo738, and *A. suecica* (in that order from outside to inside in each methylation context)."

This figure is used to claim that the overall methylation levels were higher in A. arenosa than in A. thaliana, but this might only be true for CG methylation (which appears higher visually). For CHG and CHH methylation there might be an opposite trend, but all the judgement relies on visual inspection. This inspection might also be misleading because the average methylation level needs to be adjusted by the proportion of cytosines in each context, which is usually CHH >> CHG ~= CG. Genome size is also not taken into account. Methylation levels can be defined numerically, and one common approach is to calculate the global methylation level (see Vidalis et al. 2016).

Response: We might not fully understand the comment on "CHH >> CHG ~= CG", as this does not make sense. In the reference cited (Vidalis et al., 2016), "Early methylome sequencing studies of the *A. thaliana* Columbia reference accession revealed that this model

plant methylates about 10.5% of its cytosines globally (30% in context CG, 14% in CHG, and 6% in CHH, approximately) (Zhang et al., 2006; Cokus et al., 2008; Lister et al., 2008)." We estimated global methylation levels and included the data in Source Data Extended Data Fig. 8a, b. The results indicate a similar trend to each (CG, CHG, and CHH) context; the overall methylation levels were higher in *A. arenosa* than in *A. thaliana*, especially the CG methylation.

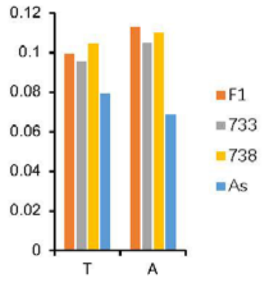

L198-200, Fig. 3b, c and Extended Fig. 7d and e: there might be a conflict in the statement and the figures. The average methylation pattern in Fig. 3b shows A. suecica with lower average levels compared to all others. Visually one might assume that correlation between F1, Allo733, Allo738 and A/T should be much higher compared to A. suecica. We might come to similar conclusions based on the very close pattern observed in Extended Figures 7d and e too. Instead the correlation between F1, Allo733, Allo738 and A/T is very low, but it's not clear why.

Response: Thanks for the comment. There were some errors in the statement. We revised, "Moreover, the average methylation levels were highly correlated between parents (Ath/Aar, T/A) and F_1, Allo733, Allo738 or *A. suecica* (at the lowest) (Extended Data Fig. 8c)."

L198, "As a result" is not right. This is about the order of presentation, not about causal relationship.

Response: We remove the "As a result" and revised to, "Moreover".

L206: not clear how the epigenomic changes are "rapid and persistent" from the previous results. No difference between parents and synthetic polyploid are discussed here, so nothing can be rapid, and there is nothing persistent. This aspect should be addressed later when looking at DMRs and removed here.

Response: This is a good comment. We revised the sentence, "These data suggest dynamic changes of epigenomic modifications in newly formed allotetraploids and natural *A. suecica*."

L207 and L248. The definition of hypo and hyper DMRs are unclear, or the terminology is confusing.
Line 207 differentially methylated regions (DMRs) between the T or A subgenome in an allotetraploid and A. thaliana (T) or A. arenosa (A), respectively
Line 248 the DMRs between the subgenomes or A. arenosa (A) and A. thaliana (T)
Are they perhaps different or the same? The authors listed four genomes and connecting them with "and" "or", which made the sentences ambiguous.
After a long struggle, I suspect that line 207 means the difference between parent and polyploid (In Figure 3d, there are 4 categories, A hyper, A hypo, T hyper, T hypo.). In contrast I suspect line 248 means the difference between subgenomes. In line 248, then, it is a relative issue. Is hypo means higher in A subgenome or in T subgenome? Throughout the text, "higher methylation levels in T subgenome" or something equivalent should be used to clarify the meaning.
Regarding DMRs, the method must be much more detailed. It describes only one type of comparison (line 708). Is this consistent with the text?

Response: As suggested, we revised Line 207, "…, we analyzed differentially methylated regions (DMRs) between T subgenome and *A. thaliana* (Ath, T genome) or A sugenome and *A. arenosa* (Aar, A genome) in each allotetraploid."

Line 248, we revised, "We further analyzed dynamic changes of hypo and hyper DMRs between Aar and Ath and between A and T subgenomes among different allotetraploids (Fig. 4b)." We also clarified them (708) in the Methods.

L214: Extended Figure 8e: not present.

Response: Thanks, and we removed the error.

L219-237 and extended data fig. 9. The correspondence between the figure and the text is unclear. There seem two duplicated genes in A. arenosa, AaROS1-1 and AaROS1-2, in the figure, but the text describes only AsROS1. Please explain it. Seeing Extended Data Fig. 9a, only thaliana homeolog of ROS1 in A. suecica is upregulated, but the text did not distinguish homeologs and said "whereas ROS1 was expressed at the highest level in A suecica". These data do not support conclusions.

Response: As suggested, we revised, "whereas *AtROS1* and *AaROS1-2* were expressed at high levels in *A. suecica* (Extended Data Fig. 10a)."

L235. "Predict" would be too strong, because there is only correlative evidence on ROS1 and methylation levels.

Response: We toned down and revised it to "speculate".

L249 and Fig. 4b. " (Fig. 4b). The number of hyper DMRs was reduced gradually from F1 to Allo733 and Allo738 and dramatically to natural A. suecica".
The way of figure presentation for this conclusion is deceiving. I do not think this conclusion is well supported. Allo733 and Allo738 are essentially biological replicates of the synthetic polyploid. It would be fair to show the two values on the same column. Then, the "gradual" pattern is not obvious with only 1 or 2 samples per class. To maintain the conclusion, please provide statistical support.

Response: We agree that Allo733 and Allo738 are biological replicates of resynthesized, which show similar levels of methylation changes. We removed "gradual" or changed to "slowly" or "slightly." "The number of hyper DMRs was reduced slightly from F_1 to resynthesized Allo733 and Allo738 and dramatically to natural *A. suecica*, while the number of hypo DMRs were relatively similar among F_1 and resynthesized allotetraploids but increased in *A. suecica*."

L263 and Fig. 4d. The legend does not explain what the dashed boxes in the figure are.

Response: As suggested, we revised to "Dashed black boxes indicate hypo DMRs between T subgenome and Ath (upper panel) and between A subgenome and Aar (lower panel) in F_1 were conserved in Allo733, Allo738 and Asu."

L269-270: how are "convergent" and "conserved" defined? What does the overlap mean? Is this from the line 241?

Response: As suggest, we briefly clarified in the Results added the definition in the Methods. "Conserved DMRs were defined as the hypo DMRs in Asu and consistently present in F_1, Allo733 or Allo738. Convergent DMRs were identified as the hyper DMRs between Aar and Ath and in F_1 and resynthesized allotetraploids and decreased to a similar level to T subgenome in Asu." The overlap between convergent and conserved groups represented those DMRs convergent in newly formed allotetraploid and remained in Asu (Fig. 4c).

L313. "predict" is far too strong. All data are a kind of cherry picking and correlative.

Response: We toned down and revised it to, "speculate".

Discussion

In this section the authors discuss DNA methylation in general terms, but most of the downstream analyses focus on CG methylation changes, meaning that most of the results refer to changes in CG context. This comes back to the comment of Fig. 3a, because not enough context is given to state that CG methylation changes represent the largest amount of changes in the genome. In addition, no DMR analysis was shown for the other two contexts. The global pattern suggests that the amount of DMRs might less, but numbers should confirm that. By better highlighting the importance and abundance of CG methylation changes, the reason behind continuing downstream analyses in CG context only would be more understandable and the discussion section would be better supported. For completeness, a short mention of the other two contexts could be considered as well.

Response: These are valid comments. We added the statistics of CHG and CHH DMRs in Extended Data Fig. 9e. We added this in the Results and also discussed. "CHG hypo DMRs in the A subgenome had a similar trend to CG hypo DMRs that increased slightly in F_1 and resynthesized allotetraploids and dramatically in *A. suecica*, while hypo DMRs in the T subgenome increased dramatically only in *A. suecica*. CHH hypo DMRs displayed a similar trend to CHG hypo DMRs, except that CHH hyper DMRs had the highest number in the T subgenome among all allotetraploids. Considering that CG methylation is relatively abundant and stable and correlates with expression levels of DMR-associated genes (Fig. 3e; Extended Data Fig. 9d), we focused most analyses on CG methylation dynamics."

Line 320 and 326. The term "ecological distribution" is unclear and unconventional. The reference papers do not seem to explain it. Then, in line 326, the authors discussed "despite diverse ecological distributions". The distribution range of A. suecica is rather narrow in the genus Arabidopsis.

Response: As suggested, we remove the "despite diverse ecological distributions" and revised it to, "*A. suecica* is estimated to form at 14,000 to 300,000 years ago and distributed in northern Fennoscandia."

Methods

Method are in general fairly brief. For RNA-seq and MethylC-seq data analysis, further details of mapping should be described. In allopolyploid species, a fragment may often be mapped to two homeologous regions with the same score, and their treatment may lead to errors (for example, Kuo et al. Brief Bioinform. 2020 Mar 23;21(2):395-407; Hu et al. Brief Bioinform. 2020 Mar 27:bbaa035). How were such reads treated?

Response: We are aware of difficulties to handle and map reads in allopolyploids. As the reviewer pointed out and to the best of our knowledge, there is no "perfect" software that is

error proof. Our general practice is filter out low-quality reads using Trimmomatic (version 0.39) (Bolger et al., 2014) and map the high-quality reads using variant calling software such as Picard Toolkit (Broad Institute, 2019). SNP tables were generated between subgenomes to partition reads using unique and perfect match. The reads that are mapped onto homoeologs are divided into homoelogs with weighted scores based on the length of reads mapped. We used the same criteria for both mRNA-seq and MethylC-seq data. Detailed procedures have been updated in the Methods.

L705-706: for reproducibility purposes, these Python scripts should be available. Also, how many cytosines were found to be conserved? This should be stated in the main text to better contextualize the amount of cytosines analyzed

Response: As suggested, Python scripts were included in the Methods and Github (https://github.com/Anticyclone-op/Ara-genome-methly). Statistics of conserved C is given in Source Data Extended Data Fig. 8a, b and some numbers were also mentioned in the main text. We selected conserved C with coverage 3 or more reads and shared among all materials for further analysis. We revised "To improve data reproducibility, we used shared methylation sites (35,853,727) with conserved cytosine and 3 or more reads among different lines for further analysis (Source Data Extended Data Fig. 8a, b)."

		Total C number	Conserved C number	Final C number	Final C number of A and T comparision
24					
25	At	43,394,826	42,616,903	22,989,440	2,936,998
26	Aa	51,794,377	47,923,117	12,864,287	2,936,998
27	F_1	95,189,203	84,244,397	35,853,727	2,936,998
28	Allo733	95,187,426	85,862,202	35,853,727	2,936,998
29	Allo738	95,189,203	91,925,175	35,853,727	2,936,998
30	As	94,572,509	94,572,509	35,853,727	2,936,998

L708-713: a sliding window approach has many limitations, but in the scope of this paper it makes more sense compared to other statistical approaches. One major limitation of sliding windows concerns multiple testing and power, which is something the authors do not seem to address in their methods where a threshold p-value is set, but no multiple testing correction is applied. In addition, the cut-off values of the methylation levels need to be clarified: were these values used as a minimum difference for testing

Response: These are valid comments. Statistical significance was analyzed using Fisher's-exact-test (*FDR*<0.05), with the following cut-off values of the minimum difference of methylation levels: 0.5 for CG DMRs, 0.3 for CHG DMRs, and 0.1 for CHH DMRs. We clarified this in the Methods.

Extended Figure 8a and b: an upset plot might be better to show intersections and representing the size of the sets. An alternative would be to have the Venn diagram show circles proportional

to the size. Figure b is really hard to understand, in particular what the asterisks refer to and what is the aim of the circle for all genes. There's also no specification of how the overlap between DMRs and genes is defined: is a 1bp overlap enough to be associated to a gene?

Response: As suggested, we replaced Venn plot with upset plot. An asterisk indicates the difference between numbers of the unique CHG or CHH DMRs and their overlapping genes was significantly reduced (Fisher's exact test), indicating CHG or CHH DMRs alone are unlikely associated with genes. DMR overlapping genes were defined as those that were overlapped with DMRs within a 2-kb flanking region.

Minor points

o L38-40: to rephrase. Polyploids do not generate genomic diversity (etc.) in response to selection, domestication or adaptation.

Response: As suggested, we replaced "generate" with "possess."

o L51: typo, "and"

Response: Removed as suggested.

o L144: typo in "allopolyploids"

Response: Corrected as suggested.

o L191-192: not clear how that improves reproducibility.

Response: We replaced "reproducibility" with "comparability", "To improve data comparability, we used shared methylation sites (35,853,727) with conserved cytosine and 3 or more reads among different lines for further analysis (Source Data Extended Data Fig. 8a, b)." and also in the Methods. This should improve comparability and accuracy across different species and possibly avoid variation of sequencing depth and uniformity.

o L233-235: Extended Fig. 7f and g show a slightly higher CHH methylation level in the tetraploids compared to A/T. The pattern is not as strong for Allo733, especially on the A-side where Allo733 has lower CHH levels.

Response: CHH methylation DMRs were rather unstable, probably because siRNAs that induce RdDM are variable in each species. "A similar trend was also observed in the CHG

methylation levels of A genome (Extended Data Fig. 8f) and to a lesser degree in the CHH context (Extended Data Figs. 8g).”

o L246-247: Sentence should be less assertive.

Response: We replaced “resulted from” with “accompanied by.”

o L270: typo in “pattern”

Response: Corrected as suggested.

o L337: A. arenosa typo

Response: Corrected as suggested.

o L358, 10,00 must be 10,000

Response: Corrected as suggested.

o L386: A. lyrata typo.

Response: Corrected as suggested.

o L684: add some details about sequencing platform and coverage.

Response: “….for mRNA sequencing with three biological replicates each with ~6.5 gigabases per replicate on Illumina HiSeq X Ten platform.”

o L694: add some details about sequencing platform and coverage.

Response: “MethylC-seq libraries were constructed using a bisulfite method as previously described (Song et al., 2017) and sequenced on Illumina HiSeq X Ten platform (~11 gigabases per replicate).”

o L700: any reason why the --score_min parameter was adjusted here?

Response: The score threshold was lowered because high heterozygosity in *A. arenosa* and also the differences between parents, F_1, Allo733, and Allo738.

o Legend Fig. 1, lyrate must be lyrata. translation must be translocation.

Response: Corrected as suggested.

o Fig. 1c. The legend is too short to understand what the figure means.

Response: We revised to "Rearrangements between T (sT1-sT5) and A (sA1-sA8) subgenomes of natural Asu and putative progenitors, *A. thaliana* (Col, T1-T5) and *A. arenosa* (A subgenome of Allo738 (A1-A8). Ribbons indicate translocations between Ath and A subgenomes (black), within Ath or A subgenome (blue), and in the same chromosomes (red)."

o Extended Fig. 2a: axes unreadable.

Response: The axes were revised as suggested.

o Extended Fig. 3d,e: why are the averages different.

Response: I believe that the question is about different averages of distributions between A and T subgenomes. This could be related to overall differences between DMRs and expression levels of the two subgenomes, as shown in many other allopolyploids such as cotton, oilseed rape, and wheat.

o Extended Fig. 7: keep colors consistent

Response: As suggested, colors were changed to be consistent.

o Extended Fig. 9: ROS2 must be typo. ROS1-1 and ROS1-2?s Differentially expression must be differential expression.

Response: As suggested, we revised to, "Differential expression of methylation pathway genes including *AtROS1*, *AaROS1-1* and *AaROS1-2* in allotetraploids."

Reviewer #2 (Remarks to the Author):

The authors of the manuscript by Jiang et al., "Concerted genomic and epigenomic changes stabilize Arabidopsis allopolyploids", have generated high quality genomes for an allotetraploid species (A. suecica) and reconstituted equivalents. The latter were obtained by crossing the parental species believed to be the A. suecica ancestors, the naturally tetraploid A. arenosa (A genome) and a tetraploid version of the otherwise diploid A. thaliana (T genome). They describe that the genomes of synthetic and natural A suecica are highly similar, synthenic, and colinear, confirming the assumed ancestry as well as the genome quality. They also describe interesting differences between the frequency of polymorphism types between the A and the T genome. While most gene families are shared between A and T, lineage-specific genes include genes connected with outcrossing (A, plausible) or less explained other GO terms in T.

Response: As suggested, we added a brief description. "GO enrichment terms of the T-lineage orthogroups (1,415) included basic molecular synthesis pathways of inner membrane system and peptide synthesis, as well as endomembrane system and tRNA aminoacylation for translation in *A. thaliana* and T subgenome of *A. suecica*."

Further analyses address individual genes (FLC) or functional group of genes (self incompatibility). A large part of the work describes the status of CG/CHG/CHH DNA methylation in genic or TE context and allows to conclude a gradual convergence of the methylation status in the evolution of alloploids, with the exception of maintaining differences at specific differentially methylated regions associated with genes of some functional groups. Finally, the authors can link different degrees of methylation in their material with different expression of genes related to reproduction.

The potential to compare the natural with the resynthesized allotetraploid species Arabidopsis suecica has been and still is a rewarding model to learn what happens upon the combinations of similar but different genomes after the formation of alloploids. The inclusion of two independent synthesized allotetraploids as well as a "fresh" F1 in the experimental design is appreciated. Although a lot of literature, including contributions from the same lab, addressed similar questions before, a detailed analysis was so far hampered by the lack of good reference genomes for the arenosa subgenome. The genomic information obtained from well-chosen material provided here is certainly a valuable resource and allows most of the conclusions drawn here. That said, most of the statements are not surprising: this is positive, as the data are congruent with partial data known before, or they are plausible by expectation (e.g. outcrossing), while it limits a bit the excitement to find unexpected new insight. Naturally, and not meant critically, the data are largely of descriptive nature. This provides a rich resource for future work that can address the role of specific pathways or functional groups of genes. However, here, only the potential for such work is visible, and the brief mentioning of examples for genes for which the methylation differences and their dynamic changes might be relevant (p. 16, SMC3, PDS5A,

AFB3) leaves the reviewer rather unsatisfied, especially as equally relevant genes (other SMCs or F-box factors) are not compared as "control group".

Response: We appreciated the positive and thoughtful analysis of our work by this expert reviewer. As suggested, we included additional analysis of *SMC3* and *PDS5A* homologous genes, *SMC1*, *SMC5*, *SMC6B*, and *PDS5B*. *SMC6A* is not expressed in leaves and expression of *SMC5* did not change between newly formed allotetraploids and *A. suecica*. We revised to "CG methylation levels of these three genes and three of homologous genes (*SMC1*, *SMC6B* and *PDS5B*) of *SMC3* and *PDS5A* were reduced from Allo733 and Allo738 to *A. suecica*, and their expression was upregulated in *A. suecica*, compared to that in Ath and F_1 (Fig. 5c; Extended Data Fig. 12a-d)."

The Discussion could be freed from some redundance with the Introduction, but it is appreciated that the authors discuss the limited comparability of their system with the conditions for other allopolyploids that were likely formed at different times and between parents of larger, more diverse genomes.

Response: We revised and removed most redundancy in the Discussion and included some relevance to BYS and hypermethylation and gene loss, as suggested by another reviewer.

A few minor points could make the manuscript also stronger:
Some of the analyses involve data from A. lyrata and A. halleri, but the Introduction fails to introduce the connection of these species with the other plant material. This information should be added.

Response: As suggested, we included these sequences in the Introduction. "However, despite 1,135 genomes of *A. thaliana* have been sequenced (Consortium, 2016), and sequences of several related species including *A. lyrata* (Hu et al., 2011), *A. halleri* (Briskine et al., 2017), and *A. kamchatica* (Paape et al., 2018) are reported (Hu et al., 2011; Novikova et al., 2016), *A. arenosa* and *A. suecica* genomes are unavailable, except for a draft sequence of *A. suecica* (Novikova et al., 2017)."

Copy number and structural variants of FLC are interesting to compare, but the DNA methylation analyses (Fig. 2e), in the absence of parallel chromatin and lncRNA analysis, is more irritating than clarifying and should be deleted.

Response: We included siRNA data from published data (Ha et al., 2009) and found a correlation of siRNAs with DNA methylation changes in the *FLC* locus. Long noncoding RNA of *FLC* is an active research field, and we should avoid this topic here.

Some proofreading is recommended (e.g. line 51).

Response: We corrected this and other typos in the revision.

Reviewer #3 (Remarks to the Author):

Please find the bibliographic references used for this report at the end of it.

Key Results

In this work, the authors deliver the genomic sequences and the epigenetic landscape of Arabidopsis arenosa and the two constitutve subgenomes of the allotetraploid A. suecica (A. thaliana and A. arenosa). Jiang and col. show how the constitutive genomes of A. suecica have remained stable after polyploidization, without drastic rearrangements, contrasting the genome shock that reported for other known allopolyploids. Nevertheless, some subtle changes are reported to differentially affect the two constitutive genomes (i.e.: gene family contraction/expansion and copy number variation). More remarkably, despite the higher initial levels of DNA methylation in one of the subgenomes in newly resynthetised allotetraploids, it is reduced during the first generations after allopolyploidization, to finally converge with the less methylated subgenome in the natural A. suecica. Finally, the authors identified some genes affected by the mentioned epigenetic changes as candidates to impact
the reproductive performance during the stabilization of Arabidopsis allopolyploids.

Response: We appreciate the positive and encouraging analysis by this expert reviewer and have addressed the clarity and validity issue as follows.

Validity

In general, I am quite positive about the validity of these results and their interpretation. However, I do find some issues that should be addressed.

The genome data provided looks reliable given the quality parameters reported, and the validation of the assemblies performed. Moreover, the synteny of A. arenosa genomes with A. lyrate helps to evaluate comparatively the quality of the assembly. Regarding the strategy to sequence the A. arenosa genome, so long time hampered by its heterozygosity, I consider that introducing this genome in A. suecica, which is able to self-polinate, to get rid of the heterozygosity has some risks. This is because the possibility of having homoeologous exchanges (HEs) between subgenomes, which has been reported to happen in synthetic suecica (Henry et al., 2014). Though the chances some HEs could have got fixed in 10 generations of selfing are not negligible, it might not affect the results and/or the conclusions of this work. It should be relatively simple to control this possibility, by performing coverage analyses (Henry et al., 2014). I think this could be especially relevant for the
conclusions on expansion and contraction of gene families. Having this concern out of the way, I think that the validity of the genome quality is extensive to the downstream analyses performed.

Response: As suggested, we compared Allo738 genome with L*er* and Aar4 (a newly sequenced, Burns et al. 2020, biorxiv) genomes and identified some HEs (Source Data Extended Data Fig. 3). We found 21,461-bp sequences of A1 were related to Chr1 of L*er*. There are more (1,400,252 bp) sequences of *A. arenosa* in T1, T2, T3 and T5 chromosomes of L*er*. Although some of these could be related to assembly issue, the degree of HEs is rather small and should not affect overall interpretation of other data.

Apart from that, there is an additional issue that I think it will be worth to address. I am sure the authors are aware about the preprint available in biorxiv (Burns et al 2020) on the A. suecica genome. Though, in fact, both papers, are quite complementary and address different biological questions they do overlap in one of them that happens to have apparently contradictive results: the TEs. While Jiang et al present very similar levels of TE in all the genomes analyzed (Exteded data fig 1a), Burns et al report that the A. arenosa genome from A. suecica have twice as TEs than the A. thaliana genomes. I wonder if the authors could address this apparent contradiction or propose any explanation for it (e.g. different A. suecica accession, different quality of the assemblies, etc).

Response: The two sets of data are comparable. We also found that A genome has twice as many TEs as T genome (Table 1), but the proportion of TE base in each genome is similar. We emphasized the similarity, while one could see the difference. Based on comparative analysis of A subgenome of Allo738 with tetraploid *A. arenosa*, we found a large portion of *A. arenosa* sequence is not aligned to the Allo738 genome, which may also affect the proportion of TEs. We clarified this in the revision.

Regarding the results on the epigenomic changes, I find them quite robust as they show a very nicely consistent trend from F1 to F10 to natural A. suecica. This holds true when data is assessed in different ways (i.e. in chromosome scale, as gene body and flanking sequence or when DMR analyses are performed). Moreover, in case of any interference created by HEs, I do not think it will affect the clear trend and consistency shown by the data. Though this is not the first time the epigenomic landscape is described in an allopolyploid species, the originality of this work lays on addressing the interplay of this feature with the process of stabilization that is obligatory for the survival of polyploids. How this stability is achieved is of great interest for the evolutionary point of view, but also for agriculture given the importance of polyploids.

Response: Again, we appreciate positive assessment of our work by this expert reviewer.

My only validity issue would be with the tittle as "Concerted genomic and epigenomic changes stabilize Arabidopsis allopolyploids", suggest evidence that causally links the stability of A. suecica to the epigenomic and genomic changes described. In my opinion, this evidence would be very complex to obtain as it might not be technically possible to verify if the stability of A. suecica would be compromised when the genomic and epigenomic changes do not happen.

Alternatively, the authors report on a set of genomic and epigenomic features than can reasonably be hypothesized to be associated with the stability of A. suecica, but do no demonstrate, in my opinion, the causality expressed by the tittle. My recommendation would be to change the tittle to something like: "Concerted genomic and epigenomic changes accompanies stabilization of Arabidopsis allopolyploids" or, "Epigenomic convergence accompanies genomic stability during Arabidopsis allopolyploids formation" or
something similar. I think it would be more accurate.

Response: This is a good suggestion, we revised the title, "Concerted genomic and epigenomic changes accompany stabilization of Arabidopsis allopolyploids".

Other than these points, I also have some minor comments on the validity of particular statements that I have exposed in the section for Suggested Improvements of this report.

Significance

In my opinion, one of the pieces of added value delivered by this work is a long time awaited high-quality assembly of A. arenosa genome. This model species has enabled important work in the last two decades without having a good reference sequence. A. arenosa forms well-established auto- and allopolyploids so I would not be surprised if this work boosts the relevance and utility of this model in the study of polyploidy evolution.

In addition, this study illustrates very well the methylation landscape changes through the polyploidization process, impacting expression and generating variation that can be the raw material for adaptation. The data presented suggests that an important part of the epigenomic changes that might contribute to stabilize allopolyploids emerges as a mere consequence of polyploidy while another part is remodelled after a few generations. This rises new interesting biological questions about the role of natural selection in this process that might inspire future research

Moreover, this study identifies some genes that could be potential candidates that could inspire future reverse genetics experiments to further characterize their role in polyploid stability, in Arabidopsis but also in crops. Some of these genes make a lot of sense in the light of the literature (e.g. effect of PDS5 reported by Bian et al, 2018).

Response: Indeed, we agree that these genomic insights and resources should lead to a lot more interesting studies in the future.

Data and methodology

The procedures to assembly and annotate the genome are sufficiently described as well.

Moreover, I find the analyses performed in this work appropriate to describe and quantify the genome structure and DNA methylation landscape of A. suecica and A. arenosa. Moreover the presentation of the data in very informative and visual graphs facilitates the reading. I didn't miss any piece of data within the provided files.

Analytical approach

In general, I found the analytical approach appropriate and the sample sizes are sufficient (3 biological replicates). I did miss some statistical test for some specific pieces of data but I have mentioned those cases in mi minor comments for the Suggested improvements part.

Response: We appreciate the reviewer for thorough analyses and evaluation of our work and have addressed remaining concerns in this revision.

Suggested improvements

Major suggestions

All my major comments are manifested below in the corresponding sections of this report accordingly to the aspect involved.

Minor suggestions

Here, some suggestions that, in my opinion, might improve the paper:
-In the abstract (line18), it is mentioned that there are "concerted genomic and epigenomic diversifications in resynthesized and natural a. suecica". Personally, I find this sentence a bit confusing (considering that is the second sentence that the reader will read while approaching the paper. They my wonder: does it mean that resynthesized and the natural become different? or is it that their genomes -become different? Moreover, I also find the concept of "concerted diversification" not entirely clear. In the next lines (any case, I think that if it means that the A and T genomes become different in some aspects, two elements (A and T) are not enough to speak about diversity (diversification means diversity is enhanced).
-In my opinion, the word "diversifications" when the differences identified between the A and T genomes are referred to is not accurate, as two elements (A and T) are not enough to speak about diversity (as diversification suggests that diversity is enhanced). For instance, the word diversification is very appropriate to speak about the multiple species of Gossypium. However in this case…How about using the word "variation".

Response: As suggested, we replaced "diversification" with "variation", " diversity" or simply "changes". What meant is that resynthesized and natural allotetraploids have consistent and different genetic and epigenetic changes relative to their progenitors. We

reserve diversification in two places, "balanced genomic diversifications in allotetraploids," which consist at least three Allo733, A. suecica, and many other A. suecica resequenced genomes (Novikova et al., 2016; Novikova et al., 2017). The latter was used to show stability of subgenomes in Allo733, Allo738 and A. suecica, in a response to the comment from another reviewer.

-The abstract mentions the concept of inter-genomic incompatibility which is not further elaborated as a problem for the stability of polyploids (lines 31 and 32).

Response: As suggested, we removed "inter-genomic incompatibility" and replaced "diversifications" with "modifications"

-Though it is true that autopolyploid bananas exist, the allopolyploid varieties are the ones that prevail in the literature. So, if I had to place bananas only in one cathegory, it would not be in the autopolyploid box without using the word "some". Line 36.

Response: It is debatable, and "bananas" is removed.

-I think it would improve the clarity and accuracy of figure1a if it includes the information (already stated in the main text) that both the parents and the F1 as well were already tetraploid.

Response: Revised, as suggested.

-The table 1 shows the size of the assembled genome. I believe it would be interesting to contrast this number with DNA contents estimated by flow cytometry.

Response: Flow cytometry estimated 1C value of 0.35 (Johnston et al., 2005), which is consistent with the Plant DNA C-values Database (release 7.1) {Pellicer, 2020 #5942}, which is ~343Mb. The genome size estimated by 19 kmer analysis is ~341 Mb for Asu, 340 Mb for Allo738, and 365 Mb for Allo733. We included this the revision.

-As it has been proposed that the genome shock accompanies whole genome duplication often involves a boost in the frequency of transposable elements, I find interesting that there are not apparent differences in TE frequencies as shown in supplementary figure 1 of the extended data. I would suggest to mention this in the text and also, if possible, to verify this statistically. Moreover, I would also suggest to discuss this result in the light of the observations from Baduel et al 2019, which reported a boost in A. arenosa autopolyploids. Does this mean that the TE levels of thaliana are higher than the levels of diploid Arenosa, but comparable to tetraploid arenosa?

Response: As in a previous response, we also found that A genome has twice as many TEs as T genome (Table 1), but the proportion of TE sequences in each genome is similar. We emphasized the similarity, while one could see the difference. Based on comparative analysis of A subgenome of Allo738 with tetraploid *A. arenosa*, we found a large portion of *A. arenosa* sequence is not aligned to the Allo738 genome, which may also affect the proportion of TEs. We clarified this in the revision. As our study does not involve population study, we should reserve our discussions from speculation, as noted by another reviewer.

-For the figure 1C. Personally, I like when the color key is shown in the figure and which is more direct than going to the text.

Response: As suggested, color key was added and also noted in the legend.

-Line 99-100. I would say "more collinear regions" rather than "higher collinear regions". Moreover, here a statistical test might be missing here.

Response: Revised, as suggested, and also added the Fisher's exact test.

-Lines 102-106. From the figure I understand that the T genome shows higher SNP density in translocations between subgenomes, but I find the text a bit difficult to understand this. Likewise it is not very clear for me whether those SNPs are between subgenomes or between progenitor and natural suecica.

Response: These SNPs are between progenitor and natural *A. suecica*. We revised to clarify the notion. "…the frequency of SNPs (between progenitor and *A. suecica*) in the A/T subgenomic translocation (homoeologous exchange) regions was twofold higher in the T than in the A subgenome (Extended Data Fig. 4c), suggesting stable maintenance of high SNP frequency in the A segment and low SNP frequency in the T segment of these exchanged regions in allotetraploids."

-Lines 110-111. In my opinion, the higher Ks between A and sA is more likely one more evidence (along with the higher frequency or rearrangements detected) that the actual donor of A-subgenome is less similar to the A-progenitors than the T-progenitor used in this study. This interpretation is more in agreement with the observations described in lines 111-112: constant rate of evolution of subgenomes.

Response: We agree completely but cannot assert this due to lack of population study, as noted by another reviewer. We revised, "The *Ks* value distribution was higher between *A. arenosa* and the A subgenome than between *A. thaliana* and the T subgenome, which is consistent with more structural variation observed in the A than T subgenome (Fig. 1c;

Extended Data Fig. 4a)." We noted in the next section, "This may suggest an increased rate of genetic diversity in the outcrossing *A. arenosa* or a different *A. arenosa* strain present in natural *A. suecica*. It is also possible that the *A. arenosa* accession used for making resynthesized allotetraploids may be different from the A-subgenome donor of *A. suecica*."

-The legend of the Extended data Figure 3a mentions s values sA vs sA. I think that the authors actually meant sA vs A.

Response: Corrected as suggested.

-In my opinion, a higher Ka does not necessarily mean faster evolution, as suggested in line lines 114-116 and extended Data Fig 3a, as it could mean simply greater distance or higher mutation rate. I think that a higher Ks/Ks, by contrast, would be indicative of faster evolution and it doesn't seem to be the case here.

Response: As suggested, we revised to "Considering that large structural variations affect the evolutionary rate (Navarro and Barton, 2003), the genic sequences in the exchanged regions between the subgenomes have slower neutral mutation rates than those in the colinear regions."

-Lines 116-117. Maybe some statistics would be required here, though no obvious differences can be seen.

Response: Wilcoxon rank sum test was performed and showed significance in Extended Data Fig. 4e.

-Lines 120-122 and figure 1f. It is suggested that polyploidy is responsible for the (on average) younger insertion time of TEs in T subgenome of A. suecica than in the progenitor. I think it would be good to provide the sample size but, in any case, it seems that most of the data contributing to the distribution is for insertion times of more than 0.3 MYA. It is not obvious for me how polyploidy can explain the differences here. I would suggest further clarification.

Response: Sorry for the confusion. We revised to "However, LTR retrotransposons were more active (younger insertion events) in the sT subgenome of *A. suecica* than in *A. thaliana Ler* and Col. Using 25 other *A. thaliana* ecotypes published previously (Gan et al., 2011; Jiao and Schneeberger, 2020), we found that all except one had older LTR insertion events than AsuT, and Kyo had similar LTR insertions time to AsuT subgenome (Extended Data Fig. 4h). This result may suggest that the T subgenome donor of Asu has more active LTRs."

-In figure 2b, I think a threshold (for significance or showing the average fold enrichment) could help the reader to understand how much the fold enrichment for those particular GO stands out.

Response: These GO terms passed the significance test of GO term enrichment. We added a dashed line to indicate onefold enrichment.

-For figure 2c, I found the blue color a bit difficult to distinguish, maybe I would recommend to use a different tone of blue. Moreover, as I mentioned, I think that adding the color code directly on the figure (rather than in the legend) would make it more comfortable to visualize.

Response: As suggested, we modified the blue color and added "+" and "-" to show gain and loss, respectively.

-Lines 155-156. Here, I find a bit difficult to suggest that reproduction genes can be (especially) fast in experiencing homoeologous exchanges. A functional bias (towards reproduction genes) would likely imply selective pressures which are difficult to assume in ten generations of lab conditions. I would just say that this informs on how fast homoeologous exchanges can happen. However, I find very intriguing the fact of having only one exon introduced which can only be explained with two events of HEs in a very narrow window of space and time. I wonder if the authors have checked if it also presents in plant 733?

Response: Thanks for the comment and sorry for the confusion. We misrepresented the data and removed the conclusion. This was an alternative splicing variant.

-In the lines 159-160, there is a double negation that is a bit confusinng "The flowering time variation was consistent with negative correlation of higher FLC expression with lower DNA methylation levels". I would just remove the "negative correlation".

Response: Removed as suggested.

-Regarding the correlations between FLC expression and methylation, though the figure 2e is nice, maybe I miss some more more quantitative information. How about showing the expression in form of FPM with the corresponding statistics?

Response: As suggested, we added ANOVA test for the TPM in these lines. "Different letters in mRNA (TPM) indicate statistical significance ($P < 0.05$, ANOVA test)."

-I miss a reference in the text to the siRNA levels shown in figure 2e. Maybe some further explanation would be nice.

Response: As suggested, we added, "These methylated regions are also target sites of siRNAs (Ha et al., 2009), which may induce RdDM."

-I don't understand (lines 173-174) why the presence of polymorphisms between A. suecica and other species suggest that there is a long term selection for selfing. Is it not that other obligate utcrossing species also have polymorphisms?

Response: This is a valid comment. We deleted the statement and revised to "In the male components, *SCR* genes are polymorphic between *A. suecica* and other species (Extended Data Fig. 7c)."

-Unless I am missing any data or previous literature, I would clarify that the silencing of the A-copy of SCR by the miRNA from the T-copy is just a prediction. I guess that the RNA- and Methyl-seq data come from tissues where the S-locus is not expressed but does it provide any information by any chance?

Response: We analysis the expression (TPM) of the *SCR* and *SRK* gene in young flowers of Allo738 and Asu. "RNA-seq data showed that *AaSCR* was not expressed in flowers of both Allo738 and Asu, while *AtSCR* expression level was very low in Allo738 and undetectable in Asu, supporting silencing of the *AaSCR* gene in these allotetraploids (Extended Data Fig. 7e)."

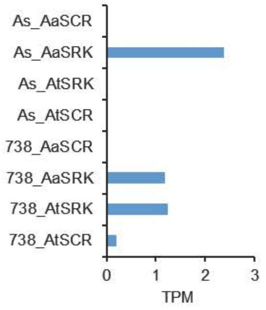

However, we did not find obvious changes of methylation levels in S locus of the T subgenome in Ath, F1, Allo733, Allo738 and Asu. For the A subgenome, methylation data cannot be compared due to high levels of polymorphism in the region.

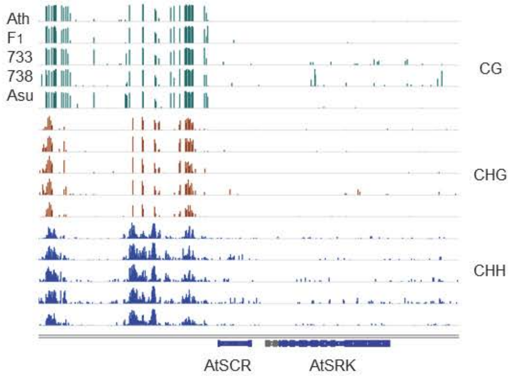

-The dominance hierarchy of the S-locus is an idea (line 181) that has not been introduced and some readers might not be totally aware of it.

Response: As suggested, we added, "Different haplotypes of the S locus have a hierarchical dominance relationship (Durand et al., 2014)."

-It is not sufficiently clear in the text (line 190) which A. thaliana plants were used to measure methylation levels, (diploid, tetraploid line, Ler, col etc..).

Response: We clarified this, "…we examined methylome diversity in *A. thaliana* (Ath, 4*x*, L*er*), *A. arenosa* (Aar, 4x), F_1, Allo733, Allo738, and natural *A. suecica* (Asu)".

-In the legend of the extended Data Fig7c. Would not it be clearer to say that it is a heatmap of pairwise comparisons of natural A. suecica, with arenosa, thaliana and F1, 733, 738.

Response: Revised, as suggested.

-Suddenly, the Allo733 is introduced in line 190 part without much information about it and the reader can just assume that is a similar to 738.

Response: As suggested, we revised, "…we examined methylome diversity in *A. thaliana* (Ath, 4*x*, L*er*4), *A. arenosa* (Aar, 4*x*), F_1, Allo738 and Allo733 (a sibling of 738) (Comai et al., 2000; Wang et al., 2006b; Shi et al., 2015), and natural *A. suecica* (Asu) (Extended Data Figs. 8a, b)."

-Regarding the comparison of expression levels in lines 224-226 and Extended Data fig 9b and 9c, again, I believe showing expression in FPM and some statistics would be nice.

Response: We performed ANOVA test and noted in the legends, "Different letters in mRNA (TPM) indicate statistical significance of $P < 0.05$ (ANVOA test, n= 3)."

-In extended data Fig10a. I would also suggest to include a straight line with any of threshold (significance, genome-wide average or something like that).

Response: Again, these are enrichment GO terms with statistical support. We added a dashed line to indicate onefold enrichment.

-Lines 310-311. For the sake of accuracy, the cited study in A. arenosa autopolyploids what actually shows is that PDS5

Response: We added the reference of PDS5 in *A. arenosa* (Yant et al., 2013).

- Lines 332-333. Again I am not entirely sure if the target prediction of one miRNA is sufficient to confirm the model proposed in this paper for the S-locus overcoming.

Response: Our expression data supports our model. To comprise, we toned down the statement. "The S locus is predicted to overcome by silencing…."

-Lines 635 636 Jukes-cantor to stimate LTR insertion age. Maybe I would include a reference for this method.

Response: As suggested, we cited Jukes-Cantor (Jukes and Cantor, 1969).

-The sequencing approaches used are appropriate for DNA and mRNA, but I maybe I missed one sentence on which technique was used for Methyl-seq. I know that a reference is provided in line 696, but I think it will not harm just to mention bisulfite sequencing.

Response: Revised, as suggested. "MethylC-seq libraries were constructed using a bisulfite method as previously described (Song et al., 2017) and sequenced on Illumina HiSeq X Ten platform (~11 gigabases)."

If the authors want to expand the scope of the study, here are three suggestions:
1. Normally, it is assumed that natural selection plays a very important role in the stabilization of polyploids. This data suggests that some of the genes potentially involved in the stability of allopolyploids undergo epigenetic changes from the F1 itself (conserved DMR). I wonder if the authors could include some ideas on the discussion on the role of natural selection for the

stability of allopolyploids (see Significance part of this report). Another way to approach this question could be to perform some analyses of the outliers of the Ka/Ks distribution regarding the epigenetic changes. Are the genes with the strongest or weakest purifying selection enriched in DMR? Is this enrichment conserved from F1, or associeated to final convergence in natural A. suecica? I think this might show a beautiful interplay between selection and methylation.

Response: We appreciate this suggestion and believe that that more population data are needed to investigate the interplay between selection and methylation. Nonetheless, we separated the genes into groups of the strongest (*Ka*/*Ks*<=0.001) and weakest (top 5%) purifying selection. We then tested an enrichment relationship between these genes and the genes related to CG hypomethylation in Asu relative to Ath or Aar. The results show that overlapping portions were statistically insignificant.

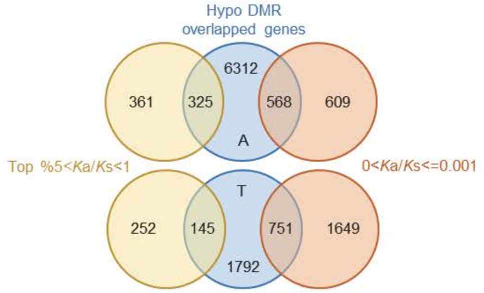

2. I am curious if there is any correlation between genes that rapidly tend to get hypermethylated (presumably silenced) and the ones that end up getting lost (family contraction). Have the authors considered including these analyses? It could be interesting to look for some meiotic genes that are known for their rapid return to single copy in paleopoliploids (De Smet et al 2013, Lloyd et al 2014).

Response: That suggestion is interesting. Among 71 meiosis-related genes (Yant et al., 2013), we found that only ASY2 (meiotic asynaptic mutant 2, homologue of ASY1) gene (Caryl et al., 2000) may be relevant. CHH methylation level of the upstream of *ASY2* was increased, and the expression level was nearly 0 in Allo733, Allo738 and Asu compared to the parent. Compared with the sequence of Col, the gene in Asu has a frameshift mutation and terminates early.

"…hypermethylation of these reproduction-related genes may lead to gene loss, as some essential genes including meiotic genes can rapidly return to single copy following genome duplication (De Smet et al., 2013; Lloyd et al., 2014). *ASY2* (asynaptic mutant2), a homolog of *ASY1* (Caryl et al., 2000), could be an example. *ASY2* is heavily methylated and expressed poorly in Allo733, Allo738, and *A. suecica* and has a frameshift mutation. However, our current data are limited to a few strains and tissue types of biological

relevance. More transcriptome, methylome, and resequencing data of *A. arenosa* and *A. suecica* populations in specific developmental stages such as meiosis are needed to interrogate this relationship between hypermethylation and retention of duplicate genes in allopolyploids."

3. I am sure that the authors have already considered this, but I wonder if it has been given any attention to THE BOY NAMED SUE locus described to impact allopolyploid stability (Henry et al 2014). E.g. methylation changes of the locus, candidate genes.

Response: Again, this is a good suggestion. We added some views in the Discussion related to the utility of the high-quality sequences. "Moreover, these high-quality sequences of *A. arenosa* and *A. suecica* should provide genomic resources for investigating interesting biological phenomena by cloning quantitative loci (QTLs) and dissecting allelic contributions in allotetraploids. *The Boy Named Sue* (*BYS*), a fertility QTL (Henry et al., 2014), spans ~240 kp on A4 chromosome, consisting of 56 annotated genes including *FIS2* (Chaudhury et al., 1997). *FIS2* is absent in *A. lyrata* and has variable sequences in *A. arenosa* and *A. suecica*. The function of candidate genes for the *BYS* locus remains to be investigated."

Clarity and accessibility

I have two major comments regarding the clarity of the paper:

1. Personally, I find the nomenclature of genomes sometimes inconsistent or unclear. I feel that depending on which part of the results we are the same subgenomes is called in different forms. I know that it is difficult, but I would suggest to stick to the same nomenclature during the entire paper. Otherwise, the reader might doubt if you mean the subgenome or the progenitor species, the F1, the synthetic or the natural one. I have the feeling that these inconsistences happen several times during the text. Here some examples that were confusing for me during my reading:
• Abstract lines 21-22. Is this meaning about Arenosa progenitor? If so, I would use the word "progenitor" to avoid ambiguity.

Response: This is a good suggestion, but it is difficult to do, as so many lines are involved. For example, we may indicate progenitor for Allo733 and Allo738 but cannot use this for natural *A. suecica*. We tried our best to make these nomenclatures consistent and yet not cumbersome (e.g., *A. thaliana*-derived subgenome, etc.). In the beginning of Results, we defined the nomenclature, "In this study, we adopted chromosome nomenclatures, T1-T5 (T subgenome) and A1-A8 (A subgenome) for resynthesized allotetraploids (Allo733 and Allo738), and sT1-sT5 and sA1-sA8 for natural *A. suecica*, while Col, L*er*2 (diploid) and

L*er*4 (tetraploid), Aar, and Asu were used to specify individual genomes or subgenomes." We are open to any suggestions for further improvement.

• Lines 91 and 92 though the figures are clear about it. I think that the text should explicitly say that the high levels of coliniarity for sA and sT are in comparison with the progenitor genomes (A and T). Otherwise it can lead to other interpretations

Response: Per suggestion, we revised, "*A. arenosa* and two subgenomes (sT and sA) of *A. suecica* have maintained high levels of colinearity and synteny compared to *A. lyrata* and extant progenitors (Aar and Ath, Col), respectively (Fig. 1b; Extended Data Figs. 1c, d). Noticeably, a large translocation between sA1 and T1 was observed in Asu (Fig. 1c), which were confirmed by a Hi-C contact matrix analysis (Fig. 1d)."

• Extended data figure 2: the 738 and As is used again.

Response: We have revised to be consistent.

• In the figures, A and T refes to the genomes from 738 and the sA and sT from the natural A. suecica. However, in the text, A and T are often referred to as the genomes from the natural A. suecica (e.g. lines 109-112).

Response: We have revised to be consistent.

• In the figure 1f. What is the difference here between T and T(738)? I understand that T Ler is from the reference of diploid Ler…

Response: This is a good suggestion to deal a complicated issue. "In this study, we adopted chromosome nomenclatures, T1-T5 (T subgenome) and A1-A8 (A subgenome) for resynthesized allotetraploids (Allo733 and Allo738), and sT1-sT5 and sA1-sA8 for natural *A. suecica*, while Col, L*er*2 (diploid) and L*er*4 (tetraploid), Aar, and Asu were used to specify individual genomes or subgenomes." We are open to any suggestions for further improvement.

• Lines 86 and 87: it is stated that the A and T subgenomes fo resynthesized will be referred to as A. arenosa (A) and A. thaliana (T, Ler). I feel it would help to state clearly that the A and T genomes from Allo738 will be there after considered as the reference for A. arenosa and A. thaliana progenitors, respectively.

Response: We removed these statements after we defined nomenclatures at the very beginning.

• In line 190, it seems that all the abbreviations are again re-defined and though, I eventually, assumed that the methylome of A arenosa was obtained from autotetraploid A. arenosa (and not from Allo 733) it was not very intuitive to me. Moreover, it is not clear what material was used for A. thaliana, is it diploid or autotetraploid Ler? Or, is it Col?

Response: We clarified this in the revision. "…we examined methylome diversity in *A. thaliana* (Ath, 4*x*, L*er*4), *A. arenosa* (Aar, 4*x*), F_1, Allo738 and Allo733 (a sibling of 738) (Comai et al., 2000; Wang et al., 2006b; Shi et al., 2015), and natural *A. suecica* (Asu) (Extended Data Figs. 8a, b)."

• Moreover, the combination of all these denominations with the use of sA (for arenosa genome within suecica) and As (for A. suecicica in general) used in some figures (e.g. figure 3) adds more confusion, in my opinion.

Response: Our intention was to simplify the nomenclature using 2 letters. In the revision, we used 3 letters (e.g., Asu for *A. suecica* and Ath for *A. thaliana*) for species designation, although we reserve our initial intention of using a shorter abbreviation.

• When the Methyl-seq results are introduced (line 190) and then, the nomenclature changes again: and T and A do no longer represent the data coming from Allo738 but from the actual thaliana and arenosa (autopolyploids? Not clarified).
In summary, I think that choosing one nomenclature and stick to it will significantly improve the reading experience and the clarity of the results. I let the authors decide which is the best system, on option might be to use full names names: A. arenosa, A. thaliana, A. suecica A, and A, suecica T. Another option could be to do something similar to the system for Brassicas (sub)genomes: Aar.A, Ath.T, Asu.A, and Asu.T combined with full names when no subgenomes is specified. Irrespective of the nomenclature chosen, it would be important to stick to it as much as possible. I know that it is slightly complex to explain that the genome reference of A. arenosa and A. thaliana actually come from Allo 738 while the methyl-seq data doesn't, but I think it can be explained.

Response: Again, "In this study, we adopted chromosome nomenclatures, T1-T5 (T subgenome) and A1-A8 (A subgenome) for resynthesized allotetraploids (Allo733 and Allo738), and sT1-sT5 and sA1-sA8 for natural *A. suecica*, while Col, L*er*2 (diploid) and L*er*4 (tetraploid), Aar, and Asu were used to specify individual genomes or subgenomes." In addition, we used 3 letters (e.g., Asu for *A. suecica* and Ath for *A. thaliana*) for species designation. We hope that these measures will help minimize or remove confusion.

2. Personally, I like when every results section finishes with one sentence summarizing the main findings. Especially when a lot of data is provided. In my opinion, this is better than finishing with an interpretation or prediction based on the data that likely fits better in the discussion. I am

totally fine with using the Results section to mention that an observation makes sense in the light of the literature (e.g. lines 142-145), but I do prefer to keep situations like proposing hypothesis (lines 155 to 156) or making predictions (e.g. lines 235 to 237 or 313 to 314) based on data for the discussion. I would suggest to slightly remodel the results part accordingly to make a more comprehensive discussion.

Response: We appreciate the encouraging comments. With respect to comments on specific genes such as *FLC* (lines 155-156) and *ROS1* (lines 235-237), we do not believe they deserve another line of Discussion, as they are relatively clear. The issue about meiosis and mitosis related genes and along with BYS locus has been included in the Discussion. In addition, the format of Nature brand journal articles limits the length of Discussion.

Other than these two issues, I feel that the paper was very accessible and the figures very illustrative, contributing to a smooth reading experience. I have some other minor suggestions to improve the clarity of some concrete parts that I have already manifested in the section of Suggested Improvements section of this report.

References
In my opinion, two minor changes regarding the references of this paper might provide a major improvement in the clarity and the strength of the paper:

-I think that if the introduction should state that the literature suggests, as the most likely origin, that autotetaploid (and not diploid) arenosa was the donor of the A genome of A. suecica. Otherwise, this could confuse the reader. For instance, someone criticism might arise from doubting whether the high methylation levels observed in synthetic allopolyploids were just a product of tens of thousands years of autopolyploidy and if the low levels of methylation in natural suecica were just a consequence of the diploid origin of its true parents. I believe that clarifying the origin of natural A. suecica in the introduction to would prevent this potential misconception, thus validating the plant materials and the approach used and the conclusions.

Response: Thanks for your suggestion. We revised, "A subgenome of *A. suecica* is reported to be more closely related to tetraploid than diploid *A. arenosa* (Novikova et al., 2016)."

-I feel that it will further strengthen the validity and the relevance of the results if Bian et al., 2017 is cited while discussing the implications of downregulation of PDS5. In this work the authors artificially reduced the expression of this gene (using VIGS) in allohexaploid wheat, which resulted in meiotic instability. I think that this provides a good validation for the results of this paper.

Response: Again, thanks for your suggestion. We included this reference. "Notably, down-regulation of *PDS5* (Traes_7DS_0DA047A5F), a homolog of *PSD5A*, and *SMC6B*

(Traes_5DL_67A6B8CEB), a homolog of *SMC3*, in allohexaploid wheat led to meiotic instability (Bian et al., 2018). Thus, low expression of these genes in *A. arenosa* is consistent with meiotic instability observed in this outcrossing autotetraploids (Yant et al., 2013)."

Bibliography
Burns et al. Gradual evolution of allopolyploidy in Arabidopsis suecica. Biorxiv. 2020. https://www.biorxiv.org/content/10.1101/2020.08.24.264432v1
Baduel et al. Relaxed purifying selection in autopolyploids drives transposable element over-accumulation which provides variants for local adaptation. 2019. Nat. Comm.
Bian et al. Meiotic chromosome stability of a newly formed allohexaploid wheat is facilitated by selection under abiotic stress as a spandrel. New Phytologist, 2018. https://doi.org/10.1111/nph.15267
Henry et al. The BOY NAMED SUE quantitative trait locus confers increased meiotic stability to an adapted natural allopolyploid of Arabidopsis. Plant Cell. 2014.10.1105/tpc.113.120626
De Smet et al. Convergent gene loss following gene and genome duplications creates single-copy families in flowering plants. 2013 PNAS. 10.1073/pnas.1300127110
De Smet et al. Meiotic gene evolution: Can you teach a new dog new tricks? 2014 Mol. Biol. Evol. 10.1093/molbev/msu119

********************END********************

References cited

Bian, Y., Yang, C., Ou, X., Zhang, Z., Wang, B., Ma, W., Gong, L., Zhang, H., and Liu, B. (2018). Meiotic chromosome stability of a newly formed allohexaploid wheat is facilitated by selection under abiotic stress as a spandrel. New Phytol **220,** 262-277.

Bolger, A.M., Lohse, M., and Usadel, B. (2014). Trimmomatic: a flexible trimmer for Illumina sequence data. Bioinformatics **30,** 2114-2120.

Briskine, R.V., Paape, T., Shimizu-Inatsugi, R., Nishiyama, T., Akama, S., Sese, J., and Shimizu, K.K. (2017). Genome assembly and annotation of Arabidopsis halleri, a model for heavy metal hyperaccumulation and evolutionary ecology. Mol Ecol Resour **17,** 1025-1036.

Caryl, A.P., Armstrong, S.J., Jones, G.H., and Franklin, F.C. (2000). A homologue of the yeast HOP1 gene is inactivated in the Arabidopsis meiotic mutant asy1. Chromosoma **109,** 62-71.

Chaudhury, A.M., Ming, L., Miller, C., Craig, S., Dennis, E.S., and Peacock, W.J. (1997). Fertilization-independent seed development in Arabidopsis thaliana. Proc Natl Acad Sci U S A **94,** 4223-4228.

Cokus, S.J., Feng, S., Zhang, X., Chen, Z., Merriman, B., Haudenschild, C.D., Pradhan, S., Nelson, S.F., Pellegrini, M., and Jacobsen, S.E. (2008). Shotgun bisulphite sequencing of the Arabidopsis genome reveals DNA methylation patterning. Nature **452,** 215-219.

Comai, L., Tyagi, A.P., Winter, K., Holmes-Davis, R., Reynolds, S.H., Stevens, Y., and Byers, B. (2000). Phenotypic instability and rapid gene silencing in newly formed *Arabidopsis* allotetraploids. Plant Cell **12,** 1551-1568.

Consortium, G. (2016). 1,135 Genomes Reveal the Global Pattern of Polymorphism in Arabidopsis thaliana. Cell **166,** 481-491.

de la Chaux, N., Tsuchimatsu, T., Shimizu, K.K., and Wagner, A. (2012). The predominantly selfing plant Arabidopsis thaliana experienced a recent reduction in transposable element abundance compared to its outcrossing relative Arabidopsis lyrata. Mob DNA **3,** 2.

De Smet, R., Adams, K.L., Vandepoele, K., Van Montagu, M.C., Maere, S., and Van de Peer, Y. (2013). Convergent gene loss following gene and genome duplications creates single-copy families in flowering plants. Proc Natl Acad Sci U S A **110,** 2898-2903.

Douglas, G.M., Gos, G., Steige, K.A., Salcedo, A., Holm, K., Josephs, E.B., Arunkumar, R., Agren, J.A., Hazzouri, K.M., Wang, W., Platts, A.E., Williamson, R.J., Neuffer, B., Lascoux, M., Slotte, T., and Wright, S.I. (2015). Hybrid origins and the earliest stages of diploidization in the highly successful recent polyploid Capsella bursa-pastoris. Proc Natl Acad Sci U S A **112,** 2806-2811.

Durand, E., Meheust, R., Soucaze, M., Goubet, P.M., Gallina, S., Poux, C., Fobis-Loisy, I., Guillon, E., Gaude, T., Sarazin, A., Figeac, M., Prat, E., Marande, W., Berges, H., Vekemans, X., Billiard, S., and Castric, V. (2014). Dominance hierarchy arising from the evolution of a complex small RNA regulatory network. Science **346,** 1200-1205.

Gan, X., Stegle, O., Behr, J., Steffen, J.G., Drewe, P., Hildebrand, K.L., Lyngsoe, R., Schultheiss, S.J., Osborne, E.J., Sreedharan, V.T., Kahles, A., Bohnert, R., Jean, G., Derwent, P., Kersey, P., Belfield, E.J., Harberd, N.P., Kemen, E., Toomajian, C., Kover, P.X., Clark, R.M., Ratsch, G., and Mott, R. (2011). Multiple reference genomes and transcriptomes for Arabidopsis thaliana. Nature **477,** 419-423.

Ha, M., Lu, J., Tian, L., Ramachandran, V., Kasschau, K.D., Chapman, E.J., Carrington, J.C., Chen, X., Wang, X.J., and Chen, Z.J. (2009). Small RNAs serve as a genetic buffer against genomic shock in *Arabidopsis* interspecific hybrids and allopolyploids. Proc Natl Acad Sci USA **106,** 17835-17840.

Henry, I.M., Dilkes, B.P., Tyagi, A., Gao, J., Christensen, B., and Comai, L. (2014). The BOY NAMED SUE quantitative trait locus confers increased meiotic stability to an adapted natural allopolyploid of Arabidopsis. Plant Cell **26,** 181-194.

Hu, T.T., Pattyn, P., Bakker, E.G., Cao, J., Cheng, J.F., Clark, R.M., Fahlgren, N., Fawcett, J.A., Grimwood, J., Gundlach, H., Haberer, G., Hollister, J.D., Ossowski, S., Ottilar, R.P., Salamov, A.A., Schneeberger, K., Spannagl, M., Wang, X., Yang, L., Nasrallah, M.E., Bergelson, J., Carrington, J.C., Gaut, B.S., Schmutz, J., Mayer, K.F., Van de Peer, Y., Grigoriev, I.V., Nordborg, M., Weigel, D., and Guo, Y.L.

(2011). The Arabidopsis lyrata genome sequence and the basis of rapid genome size change. Nat Genet **43,** 476-481.

Jiao, W.B., and Schneeberger, K. (2020). Chromosome-level assemblies of multiple Arabidopsis genomes reveal hotspots of rearrangements with altered evolutionary dynamics. Nat Commun **11,** 989.

Johnston, J.S., Pepper, A.E., Hall, A.E., Chen, Z.J., Hodnett, G., Drabek, J., Lopez, R., and Price, H.J. (2005). Evolution of genome size in Brassicaceae. Ann Bot-London **95,** 229-235.

Jukes, T.H., and Cantor, C.R. (1969). Evolution of protein molecules. In Mammalian Protein Metabolism, H.N. Munro, ed (New York: Academic Press), pp. 21-132.

Lister, R., O'Malley, R.C., Tonti-Filippini, J., Gregory, B.D., Berry, C.C., Millar, A.H., and Ecker, J.R. (2008). Highly integrated single-base resolution maps of the epigenome in Arabidopsis. Cell **133,** 523-536.

Lloyd, A.H., Ranoux, M., Vautrin, S., Glover, N., Fourment, J., Charif, D., Choulet, F., Lassalle, G., Marande, W., Tran, J., Granier, F., Pingault, L., Remay, A., Marquis, C., Belcram, H., Chalhoub, B., Feuillet, C., Berges, H., Sourdille, P., and Jenczewski, E. (2014). Meiotic gene evolution: can you teach a new dog new tricks? Mol Biol Evol **31,** 1724-1727.

Nah, G., and Jeffrey Chen, Z. (2010). Tandem duplication of the FLC locus and the origin of a new gene in Arabidopsis related species and their functional implications in allopolyploids. New Phytol **186,** 228-238.

Navarro, A., and Barton, N.H. (2003). Chromosomal speciation and molecular divergence--accelerated evolution in rearranged chromosomes. Science **300,** 321-324.

Ni, Z., Kim, E.D., Ha, M., Lackey, E., Liu, J., Zhang, Y., Sun, Q., and Chen, Z.J. (2009). Altered circadian rhythms regulate growth vigour in hybrids and allopolyploids. Nature **457,** 327-331.

Novikova, P.Y., Tsuchimatsu, T., Simon, S., Nizhynska, V., Voronin, V., Burns, R., Fedorenko, O.M., Holm, S., Sall, T., Prat, E., Marande, W., Castric, V., and Nordborg, M. (2017). Genome Sequencing Reveals the Origin of the Allotetraploid Arabidopsis suecica. Mol Biol Evol **34,** 957-968.

Novikova, P.Y., Hohmann, N., Nizhynska, V., Tsuchimatsu, T., Ali, J., Muir, G., Guggisberg, A., Paape, T., Schmid, K., Fedorenko, O.M., Holm, S., Sall, T., Schlotterer, C., Marhold, K., Widmer, A., Sese, J., Shimizu, K.K., Weigel, D., Kramer, U., Koch, M.A., and Nordborg, M. (2016). Sequencing of the genus Arabidopsis identifies a complex history of nonbifurcating speciation and abundant trans-specific polymorphism. Nat Genet **48,** 1077-1082.

Paape, T., Briskine, R.V., Halstead-Nussloch, G., Lischer, H.E.L., Shimizu-Inatsugi, R., Hatakeyama, M., Tanaka, K., Nishiyama, T., Sabirov, R., Sese, J., and Shimizu, K.K. (2018). Patterns of polymorphism and selection in the subgenomes of the allopolyploid Arabidopsis kamchatica. Nat Commun **9,** 3909.

Shi, X., Zhang, C., Ko, D.K., and Chen, Z.J. (2015). Genome-Wide Dosage-Dependent and -Independent Regulation Contributes to Gene Expression and Evolutionary Novelty in Plant Polyploids. Mol Biol Evol **32,** 2351-2366.

Shi, X., Ng, D.W., Zhang, C., Comai, L., Ye, W., and Jeffrey Chen, Z. (2012). Cis- and trans-regulatory divergence between progenitor species determines gene-expression novelty in Arabidopsis allopolyploids. Nat Commun **3,** 950.

Song, Q., Zhang, T., Stelly, D.M., and Chen, Z.J. (2017). Epigenomic and functional analyses reveal roles of epialleles in the loss of photoperiod sensitivity during domestication of allotetraploid cottons. Genome Biol **18,** 99.

Vidalis, A., Zivkovic, D., Wardenaar, R., Roquis, D., Tellier, A., and Johannes, F. (2016). Methylome evolution in plants. Genome Biol **17,** 264.

Wang, J., Tian, L., Lee, H.S., and Chen, Z.J. (2006a). Nonadditive Regulation of *FRI* and *FLC* Loci Mediates Flowering-Time Variation in *Arabidopsis* Allopolyploids. Genetics **173,** 965-974.

Wang, J., Tian, L., Lee, H.S., Wei, N.E., Jiang, H., Watson, B., Madlung, A., Osborn, T.C., Doerge, R.W., Comai, L., and Chen, Z.J. (2006b). Genomewide nonadditive gene regulation in *Arabidopsis* allotetraploids. Genetics **172,** 507-517.

Yant, L., Hollister, J.D., Wright, K.M., Arnold, B.J., Higgins, J.D., Franklin, F.C.H., and Bomblies, K. (2013). Meiotic adaptation to genome duplication in Arabidopsis arenosa. Curr Biol **23,** 2151-2156.

Zapata, L., Ding, J., Willing, E.M., Hartwig, B., Bezdan, D., Jiao, W.B., Patel, V., Velikkakam James, G., Koornneef, M., Ossowski, S., and Schneeberger, K. (2016). Chromosome-level assembly of Arabidopsis thaliana Ler reveals the extent of translocation and inversion polymorphisms. Proc Natl Acad Sci U S A **113,** E4052-4060.

Zhang, X., Yazaki, J., Sundaresan, A., Cokus, S., Chan, S.W., Chen, H., Henderson, I.R., Shinn, P., Pellegrini, M., Jacobsen, S.E., and Ecker, J.R. (2006). Genome-wide high-resolution mapping and functional analysis of DNA methylation in Arabidopsis. Cell **126,** 1189-1201.

natureresearch

Decision Letter, first revision:

6th April 2021

*Please ensure you delete the link to your author homepage in this e-mail if you wish to forward it to your co-authors.

Dear Jeff,

Your revised manuscript entitled "Concerted genomic and epigenomic changes accompany stabilization of Arabidopsis allopolyploids" has now been seen by the same three reviewers, whose comments are attached. The reviewers state that the manuscript has been improved but they still have a number of concerns, mostly regarding presentation, which will need to be addressed before we can offer publication in Nature Ecology & Evolution. We will therefore need to see your responses to the criticisms raised and to some editorial concerns, along with a revised manuscript, before we can reach a final decision regarding publication.

We therefore invite you to revise your manuscript taking into account all reviewer and editor comments. Please highlight all changes in the manuscript text file in Microsoft Word format.

We are committed to providing a fair and constructive peer-review process. Do not hesitate to contact us if there are specific requests from the reviewers that you believe are technically impossible or unlikely to yield a meaningful outcome.

When revising your manuscript:

* Include a “Response to reviewers” document detailing, point-by-point, how you addressed each reviewer comment. If no action was taken to address a point, you must provide a compelling argument. This response will be sent back to the reviewers along with the revised manuscript.

* If you have not done so already please begin to revise your manuscript so that it conforms to our Article format instructions at http://www.nature.com/natecolevol/info/final-submission. Refer also to any guidelines provided in this letter.

* Include a revised version of any required reporting checklist. It will be available to referees (and, potentially, statisticians) to aid in their evaluation if the manuscript goes back for peer review. A revised checklist is essential for re-review of the paper.

Please use the link below to submit your revised manuscript and related files:

[REDACTED]

Note: This URL links to your confidential home page and associated information about manuscripts you may have submitted, or that you are reviewing for us. If you wish to forward this email to co-authors, please delete the link to your homepage.

We hope to receive your revised manuscript within four to eight weeks. If you cannot send it within this time, please let us know. We will be happy to consider your revision so long as nothing similar has been accepted for publication at Nature Ecology & Evolution or published elsewhere.

Nature Ecology & Evolution is committed to improving transparency in authorship. As part of our efforts in this direction, we are now requesting that all authors identified as ‘corresponding author’ on published papers create and link their Open Researcher and Contributor Identifier (ORCID) with their account on the Manuscript Tracking System (MTS), prior to acceptance. ORCID helps the scientific community achieve unambiguous attribution of all scholarly contributions. You can create and link your ORCID from the home page of the MTS by clicking on ‘Modify my Springer Nature account’. For more information please visit please visit www.springernature.com/orcid.

Please do not hesitate to contact me if you have any questions or would like to discuss these revisions further.

We look forward to seeing the revised manuscript and thank you for the opportunity to review your work.

Yours sincerely,

[REDACTED]

Reviewers' comments:

Reviewer #1 (Remarks to the Author):

"Reply:" indicates the comments by reviewers below.

Reviewer #1 (Remarks to the Author):
The authors focused on the genomic and epigenomic analysis of a model allopolyploid species Arabidopsis suecica. The authors first conducted chromosome-scale assembly of natural and synthetic A. suecica. The latter was used as a substitute of a parental species A. arenosa, which I will discuss further below. Standard analyses of synteny and gene families as well as a focus on flowering and self-incompatibility genes follow. The authors did not find a major change that may be called genome shock. Then, the authors reported the main part of this manuscript, genome-wide DNA methylation analysis. Similar to previous studies using cotton, the methylation level of the two subgenomes became similar after hybridization, but in cotton low methylated subgenome became highly methylated in contrast to the decrease of high methylated subgenome in A. suecica. The authors identified differentially methylated regions.
The topic of epigenome of polyploid species is topical, and the dataset of the model allopolyploid A. suecica would be valuable. However, writing should be substantially improved. The methods and figure legends are often too short to follow. The correspondence between the main text and figures are often unclear.
Response: We appreciate the encouraging and constructive analysis of our work from this expert reviewer and have addressed the concerns in this revision.

Reply: Many points were indeed improved. However, I wished the authors took this point more thoroughly: "However, writing should be substantially improved. The methods and figure legends are often too short to follow. The correspondence between the main text and figures are often unclear." The points that are directly pointed out by reviewers are improved but many points indirectly related to them are left. I would mention some of them in the following.

A major issue that would need additional analysis is a circular argument about the genome conservation. The authors did not assemble directly the parental species A. arenosa, but the arenosa-derived subgenome of the synthetic A. suecica which experienced about 10 generations after allopolyploidization. Technically, it is a good idea to obtain homozygous state, because high heterozygosity of the autotetraploid A. arenosa would be a major barrier for genome assembly. The authors simply said in line 86 "Because of genome conservation, our further analysis considered the A and T subgenomes of resynthesized Allo738 to be A. arenosa (A) and A. thaliana (T, Ler) genomes, respectively." However, validation and careful interpretation would be necessary. It is not clear what aspect the authors say "genome conservation", or between which individuals the genomes were conserved. In other words, in this experimental setting, they cannot compare the genome before and after the hybridization (I mean, allopolyploidization). They were comparing the sequence right after allopolyploidization and that after thousands of years.
In DNA sequences, it is no surprise if there were not be major changes in this laboratory 10 generations, if so, the genome may be conserved at the allopolyploidization event. However, there is no direct evidence shown in the manuscript. I would propose a few possibilities to check if the changes in the 10 generations were not huge, although the authors may find additional ways. First of all, at least, the Ler subgenome of synthetic A. suecica should be compared with the Ler genome. Fig. 1c showed a chromosome level synteny with Col accession of A. thaliana, but this is not adequate because the authors discussed smaller scales of changes in the text. There is a publication (Chromosome-level assembly of Arabidopsis thaliana Ler reveals the extent of translocation and inversion polymorphisms. Zapata et al. Proc Natl Acad Sci U S A. 2016 Jul 12;113(28):E4052-60) although I do not know if it is directly usable for your purpose. To compare arenosa subgenome with A. arenosa would be more important. Small amount of long- read data from natural A. arenosa (ideally close to the parent of the synthetic polyploid) may be adequate to validate the assembly. Even when these validations are done, all texts including the abstract, results and discussion should be toned down when it comes to the interpretation of the conservation at the allopolyploidization.
Response: These are valid comments. We thank Dr. Magnus Nordborg for sharing A. arenosa sequence (bioRxiv, Burns et al. 2020). “To test stability of resynthesized Arabidopsis allotetraploid Allo738, we compared the genome of Allo738 with Ler (Zapata et al., 2016) and other Arabidopsis species (Novikova et al., 2016; Novikova et al., 2017) including an A. arenosa accession (https://doi.org/10.1101/2020.08.24.264432), and Allo733 (a sibling of 738) (Extended Data Fig. 3). We found that (1) Allo733 and Allo738 have similar levels of divergence to Ler and Aar4, respectively (Extended Data Fig. 3a); (2) A subgenomes of Allo733 and Allo738 are closely related to A. arenosa accessions that are in a different clade from A subgenomes of A. suecica accessions (Extended Data Fig. 3b); and (3) T subgenome of Allo733 and Allo738 are closely related to Ler, which is different from T subgenome of A. suecica accessions (Extended Data Fig. 3c). Neighbor-joining evolutionary tree also indicated that the A-subgenome donor of Allo733 and Allo738 was closest to A. arenosa (Novikova et al., 2017) (Extended Data Fig. 3b), and T-subgenome donor of Asu was closely related to ecotypes from Russia of Asia admixture of 1135 strains analyzed (Extended Data Fig. 3c) (Consortium, 2016; Novikova et al., 2016). These analyses confirm that A and T subgenomes of resynthesized Allo738 (and Allo733) can be treated as A. arenosa (Aar) and A. thaliana (Ath, Ler) genomes, respectively, for further analysis.” We also updated source data with these new analyses.

Reply: I appreciate the substantial effort of validation by the authors, but it only deals with the large scale synteny. This may be fine for epigenetic analysis. However, the validation at more fine scales is lacking, although the assumption that A-subgenome of A. suecica represent A. arenosa sequence probably exist for DNA sequencing analysis. The method section is too short to tell which ones do or do not include this assumption. For example, the Ka/Ks calculations are likely suffered from it. There would be two feasible solutions. First, validation can be done at a single nucleotide level. For the T-subgenome, it can be straightforward. Is the sequence of the published Ler genome and the newly assembled T-subgenome of the synthetics nearly identical? (If not, it may be interesting but raises new questions). It may not be necessary to evaluate complete genome. Note that phylogenetic tree shown in Extended Dat Fig. 3 is not very informative at a fine scale (it just takes the pattern of majority). Second, the SNPs of the natural arenosa may be used for the Ka/Ks, now that you included such data. Also for analyses other than Ka/Ks, I hope authors would check the validation carefully.
Track Line 2411. "Identification of orthologous genes and Ka/Ks calculations. Orthologous gene clusters were recognized using OrthoFinder 108 (version 2.2.7) using parameters (-S diamond -M msa -T raxml) 109. The single-copy genes of A. thaliana, A. arenosa, A. suecica, and A. lyrata were used to calculate Ks, Ka, and Ka/Ks values 110 by KaKs_Calculator (version 1.2) 111.
In short, I do not think the following statement by the author is valid. " These analyses confirm that A and T subgenomes of resynthesized Allo738 (and Allo733) can be treated as A. arenosa (Aar) and A. thaliana (Ath, Ler) genomes, respectively, for further analysis. "
Track line 605. In a new sentence, "Kyo" appears without explanation. Please explain it. Citing a reference is not enough (readers or reviewers are not expected to spend effort to obtain the reference). From the context, it may be a thaliana "ecotype from Russia of Asia". By the way, "Russia of Asia" must be a typo.

Extended Data Fig. 1. What is the "proportion" of genes and TEs? Is it per base? If so, how did you classify the genome, gene, TE and intergenic regions?

In interpreting and discussing the results, the authors should consider seriously that variation within species cannot be examined with the analyzed samples. I do not mean that the authors should be increase the data, but the authors often consider the studied individuals as the representatives of a species. The manuscript did not discuss the distance between the individual of A. arenosa the author used to make the synthetic polyploid and the individual(s) that contributed to the origin of A. suecica 14,000-300,000 years ago. A. arenosa includes many subspecies and thus the distance can be fairly big. This difference would be particularly important for traits that are polymorphic within species, such as transposon insertion or small- scale rearrangement. The author did mention this general issue in studying polyploid species in the discussion line 357 "The species or strains used to form B. napus or wheat 8,000-10,00 years ago 60 may become extinct and different from the existing species." This is the same for their own data. The first example is found in line 95. "Interestingly, inversions and translocations occurred more frequently between A. arenosa and the A subgenome than between A. thaliana and the T subgenome of A. suecica (Fig. 1c; Extended Data Fig. 3a). This may suggest an increased rate of genetic diversity in the outcrossing A. arenosa or a different A. arenosa strain present in natural A. suecica." However, another simple explanation is that the genotype of A. arenosa the authors used may be different from the very individual which contributed to the allopolyploidization 14,000-300,000 years ago. Thus the statement cannot be simply defended. Similarly, line 109 said " The Ks value distribution was higher between A. arenosa and the A subgenome than between A. thaliana and the T subgenome, suggesting a faster mutation rate in A. arenosa", but the issue is the same. If authors would like to discuss this point, population data of A.

arenosa may give some insights, but it is difficult to exclude the possibility that unknown genotype existed previously, and so I would recommend to remove this conclusion. I do not think it is the reviewer's job to point out all similar issues throughout the manuscript, and I wish the authors would revise accordingly.
Response: We appreciate this comment on data interpretation. As noted by the reviewer, we did not intend to study diversity within progenitor species and presented alternative possibilities. For line 95, we did include this alternative notion (see below). For Ks value analysis, we revised, "The Ks value distribution was higher between A. arenosa and the A subgenome than between A. thaliana and the T subgenome, which is consistent with more structural variation observed in A than T subgenome." We added, "This may suggest an increased rate of genetic diversity in the outcrossing A. arenosa or a different A. arenosa strain involved in the formation of natural A. suecica." From the published A. arenosa resequencing data (Burns et al. 2020, biorxiv), we found that the A. areonsa (Aar4) is indeed relatively close to the A genome donor of A. suecica. This conclusion is also consistent with the phylogenetic data previously reported (Novikova et al., 2016) (Extended Data Fig. 3). We have checked and toned down other relevant statements.
Reply: OK.

Abstract
In general, the abstract does not correspond to the content well.
L27 The sentence on results of self-incompatibility is not correct. The authors said "These epigenetic processes in the allotetraploids affect gene expression and phenotypic variation, including flowering, silencing of self-incompatibility". However, the result section described just the sequences of small RNA and its potential binding sites, which is nothing to do with "These epigenetic processes", which refer to DNA methylation in the previous sentence.

Response: As suggested, we revised the sentence. "These epigenetic processes including small RNAs in the allotetraploids may affect gene expression and phenotypic variation, including flowering, silencing of self-incompatibility, and upregulation of meiosis- and mitosis-related genes."
Reply: It is fine although the sentence does not have a significant conclusion.

Results
L103, explanations other than homeologous exchanges seems also plausible. Please explain more if this conclusion is retained.
Response: This could be a confusion. It was meant, as expected, that the SNP frequency in the T segment translocated to A subgenome is low, and the SNP frequency in the A segment translocated to in T subgenome is high. This suggests stable maintenance of high SNP frequency in the A segment and low SNP frequency in the T segment of these exchanged regions in allotetraploids. We clarified this in the revision.
Reply: This is fine. Related to that, there is a small issue.
Legend of Extended Data Fig. 4
The term TLb is used without definition. After spending a while, it turned out that it was defined in the legend of Fig. 1 (translocation between subgenomes). These should be defined in the main text or in each legend separately. This is just a tip of iceberg that makes this manuscript difficult to read through.

L117. The authors detected purifying selection, but no conclusions are drawn. There are already a few papers addressing the question whether purifying selection is weaker due to redundancy of homeologs in polyploid species (Capsella bursa-pastoris by Douglas et al. Proc Natl Acad Sci U S A. 2015 Mar

3;112(9):2806-11, A. kamchatica by Paape et al. Nat Commun. 2018 Sep 25;9(1):3909). These papers should be discussed in this context.
Response: That's a valid comment. As suggested, we added a sentence to clarify this. "However, purifying selection is generally weaker due to redundancy of homoeologs in allopolyploids as reported in A. kamchatica (Douglas et al., 2015) and Capsella bursa (Paape et al., 2018), and allopolyploidy might have weakened natural selection because of this bottleneck effect."
Reply: Just a typo (Doublas and Paape are opposite). Otherwise it is fine.

L118. Similarly, no reference was cited in the paragraph on the insertion time of transposable elements. There are studies in Arabidopsis species, and for example it is reported that recent insertion in A. thaliana was reduced associated with the transition to selfing (de la Chaux et al. Mob DNA. 2012 Feb 7;3(1):2). The conclusions should be discussed in the context of previous researches.
Response: As suggested, we discussed the reduced insertion of LTR after selfing in A. thaliana. We revised to "The order of insertion time is A. thaliana > A. lyrata > A. arenosa. (Fig. 1f), which seems to correlate with different mating systems, as recent insertions in A.
thaliana were reduced from the transition of outcrossing in A. lyrata to selfing (de la Chaux et al., 2012)."
Reply: fine.

L127. The source of the A. halleri data was not found. Please add references. It may be Briskine et al. Mol Ecol Resour. 2017 Sep;17(5):1025-1036.
Response: As suggested, we cited the reference for A. halleri along with A. lyrata and A. kamchatica in the Introduction.
Reply: fine.

L132. The first sentence said similar, but the second sentence seems contradictory.
Response: We revised, "Analysis of the gene family contraction and expansion revealed uneven rates of gain or loss among allopolyploid species examined (Fig. 2c)."

Reply: fine

Fig. 2. A. lyrata and A. halleri are clustered and had zero common changes. Many phylogenetic studies including Novikova et al. Nat Genet. 2016 Sep;48(9):1077-82 showed that lyrata and arenosa cluster first, and then halleri comes outside. I hope then the data would make sense.
Response: This is a good comment. We have revisited the analysis of trees, and the result remained unchanged. This could be due to closeness of A. arenosa to A. suecica and a small number of species used in this study. We stated, "Note that clustering between A. lyrata and
A. halleri could result from a small number of species used in the study, while A. lyrata and
A. arenosa may be more closely related (Novikova et al., 2016)." However, this discrepancy does not affect interpretation of the data.
Reply: The method section is too short (only 5 lines) and so more details would be necessary.

Track line 618.
The text of the original version corresponded to Fig. 2b. However, after revision, new categories were added in the text but the figure is unchanged. Please clarify.

Track line 614 The meaning of "Gene families (744) specific to the A-lineage orthogroups from A. arenosa, A. suecica, A. lyrata, and A. halleri" is not understandable. In Vendiagram of a or in c, the number 614 is not found. Then, it may mean something else. "A-lineage" of A. suecica can make sence because it has two homeologs. However, why A- or T- lineage matter for A. lyrata and A. halleri? The method section is too short (only 5 lines) and no more information is available. I suspect the authors may mean the clade including A. arenosa, A. suecica A, A. halleri, A. lyrata. However, it is not clear what the meaning of including A. lyrata or A. halleri. In addition, as mentioned before, this tree shape contradicts with a consensus tree of the genus, and so there may be some methodological issues.
Legend if Fig. 2
Black dots indicate node T (ancestor of A. thaliana) is probably wrong. Seeing the brief method, it is likely to be the common ancestor of A. thaliana col and the T-subgenome of A. suecica. The same for Node A.

Fig. 2e. It is unclear which tissues were used for the analysis.
Response: Rosette leaves before bolting, 3-4 weeks for A. thaliana and 6-7 weeks for A. arenosa, F1, Allo733, Allo738, and A. suecica, as previously reported in several studies (Wang et al., 2006a; Wang et al., 2006b; Ni et al., 2009; Shi et al., 2012; Shi et al., 2015) to standardize the stage of “prior to bolting.”
Reply: fine.

L155 Extended Data Fig. 5a,b do not seem relevant for the result of the sentence ("gain an exon from AaFLC1"). No figure legend explains a particular exon.
Response: The figure shows gene structure of the longest transcript. We removed the sentence, which is a previously reported result (Nah and Jeffrey Chen, 2010).
Reply: This is fine but another issue is found.

Track line 709.
Regarding the new sentence, no causal evidence was shown. At most, siRNA and DNA methylation "may be involved."

Track line 701
This does not seem to fit to the normal definition of convergent evolution (rather opposite). The authors potentially may mean that As-AaFLC1 and As-AaFLC2 forms a clade, this may be called gene conversion between tandem repeats (which is often called concerted evolution in the case of rDNA evolution). Still, the bootstrap of this clade is only 66, and this alone is not really a strong evidence.

L174-184. The description and discussion on the self-incompatible genes should be revised thoroughly. First, the result in the line 178-180 "In A. suecica, the AaSCR allele is silenced by miR867 of SCR04 targeting the first exon of AaSCR with a frameshift mutation (Extended Data Fig. 6d)" was already reported by Novikova et al. (reference 15) and thus it should be cited here. There are indeed new results. It is unclear why "a long-term selection for selfing in the allotetraploid, leading to nonfunctional S-alleles" is suggested. With a single disruptive mutation in S genes, self-compatibility

can evolve, and thus long-term selection is not relevant. More importantly, despite many studies, it has been difficult to detect the selection on self-compatibility from molecular evidences, and there is no molecular evidence in A. suecica supporting selection. A. suecica could have obtained self-compatibility at the origin, then selection was not relevant. Relevant review paper (for example, Shimizu and Tsuchimatsu, Ann Rev Ecol Evol Syst 46, 593-622, 2015) would provide further information. It is unclear what "gradual loss of self-incompatibility" in line 183 means. The sentence indicates no data or reference.
Reply: It is substantially improved.
Track line 762. It is unclear why "the combination of weak alleles" matters. Literally interpreted, it means two weak alleles were combined. That is nothing to do with the mechanism. The authors may mean the weak allele SCR01.

Response: These are valid comments. We removed the statement of long-term selection and gradual loss. Our intention was to discriminate the early stages of self-incompatibility in Allo733 and Allo738 (1-5 generations) and sequence variation between natural A. suecica and A. kamchatica. We added reference 15 in the line and clarified these results in the Results.

Reply: fine.

L194 and Fig.3a: it is not clear which row represents which individual, a second legend might be needed. There are five rows but the legend list six taxa. If a reader is very careful, one might notice that A. thaliana and A. arenosa are half and half in the same row, but still, it is not shown whether outside or inside correspond to them.
Response: As suggested, we revised the Fig 3a to "a, Chromosome features and methylation distributions. Notes in circos plots: (1) chromosomes, (2) gene and (3) TE density, and (4) CG, (5) CHG and (6) CHH methylation levels using 100-kb windows in A. thaliana or A. arenosa, F1, Allo733, Allo738, and A. suecica (in that order from outside to inside in each methylation context)."
Reply: fine.

This figure is used to claim that the overall methylation levels were higher in A. arenosa than in A. thaliana, but this might only be true for CG methylation (which appears higher visually). For CHG and CHH methylation there might be an opposite trend, but all the judgement relies on visual inspection. This inspection might also be misleading because the average methylation level needs to be adjusted by the proportion of cytosines in each context, which is usually CHH >> CHG ~= CG. Genome size is also not taken into account. Methylation levels can be defined numerically, and one common approach is to calculate the global methylation level (see Vidalis et al. 2016).
Response: We might not fully understand the comment on "CHH >> CHG ~= CG", as this does not make sense. In the reference cited (Vidalis et al., 2016), "Early methylome sequencing studies of the A. thaliana Columbia reference accession revealed that this model plant methylates about 10.5% of its cytosines globally (30% in context CG, 14% in CHG, and 6% in CHH, approximately) (Zhang et al., 2006; Cokus et al., 2008; Lister et al., 2008)." We estimated global methylation levels and included the data in Source Data Extended Data Fig. 8a, b. The results indicate a similar trend to each (CG, CHG, and CHH) context; the overall methylation levels were higher in A. arenosa than in A. thaliana, especially the CG methylation.
Reply: I probably now found the reason of the confusion in relation to the presentation. Track line 810

Pverall methylation levels were higher in A. arenosa than in A. thaliana (Fig. 3a, Source Data Extended Data Fig. 8a, b)
At a first glance, this conclusion is not visible from these figures. CG appeared higher in Aar, but CHG seems higher in Ath in Extended Data Fig. 8. Then, I noticed that the scale is different between a and b. I would suggest to use the same scale, and add explanation in the main text. Here, the main text asks the readers to compare the values of a and b. The current presentation may not be incorrect but confusing.

L198-200, Fig. 3b, c and Extended Fig. 7d and e: there might be a conflict in the statement and the figures. The average methylation pattern in Fig. 3b shows A. suecica with lower average levels compared to all others. Visually one might assume that correlation between F1, Allo733, Allo738 and A/T should be much higher compared to A. suecica. We might come to similar conclusions based on the very close pattern observed in Extended Figures 7d and e too. Instead the correlation between F1, Allo733, Allo738 and A/T is very low, but it’s not clear why.
Response: Thanks for the comment. There were some errors in the statement. We revised, “Moreover, the average methylation levels were highly correlated between parents (Ath/Aar, T/A) and F1, Allo733, Allo738 or A. suecica (at the lowest) (Extended Data Fig. 8c).”
Reply: fine.

L198, "As a result" is not right. This is about the order of presentation, not about causal relationship.
Response: We remove the “As a result” and revised to, “Moreover”.
Reply: fine.

L206: not clear how the epigenomic changes are “rapid and persistent” from the previous results. No difference between parents and synthetic polyploid are discussed here, so nothing can be rapid, and there is nothing persistent. This aspect should be addressed later when looking at DMRs and removed here.
Response: This is a good comment. We revised the sentence, “These data suggest dynamic changes of epigenomic modifications in newly formed allotetraploids and natural A. suecica.”
Reply: this is better, still what "dynamic" means is obscure. It would be better to define.

L207 and L248. The definition of hypo and hyper DMRs are unclear, or the terminology is confusing. Line 207 differentially methylated regions (DMRs) between the T or A subgenome in an allotetraploid and A. thaliana (T) or A. arenosa (A), respectively Line 248 the DMRs between the subgenomes or A. arenosa (A) and A. thaliana (T) Are they perhaps different or the same? The authors listed four genomes and connecting them with "and" "or", which made the sentences ambiguous. After a long struggle, I suspect that line 207 means the difference between parent and polyploid (In Figure 3d, there are 4 categories, A hyper, A hypo, T hyper, T hypo.). In contrast I suspect line 248 means the difference between subgenomes. In line 248, then, it is a relative issue. Is hypo means higher in A subgenome or in T subgenome? Throughout the text, "higher methylation levels in T subgenome" or something equivalent should be used to clarify the meaning. Regarding DMRs, the method must be much more detailed. It describes only one type of comparison (line 708). Is this consistent with the text?
Response: As suggested, we revised Line 207, “…, we analyzed differentially methylated regions

(DMRs) between T subgenome and A. thaliana (Ath, T genome) or A sugenome and A. arenosa (Aar, A genome) in each allotetraploid."
Line 248, we revised, "We further analyzed dynamic changes of hypo and hyper DMRs between Aar and Ath and between A and T subgenomes among different allotetraploids (Fig. 4b)." We also clarified them (708) in the Methods.

Reply: fine.

L214: Extended Figure 8e: not present.
Response: Thanks, and we removed the error.
Reply: fine.

L219-237 and extended data fig. 9. The correspondence between the figure and the text is unclear. There seem two duplicated genes in A. arenosa, AaROS1-1 and AaROS1-2, in the figure, but the text describes only AsROS1. Please explain it. Seeing Extended Data Fig. 9a, only thaliana homeolog of ROS1 in A. suecica is upregulated, but the text did not distinguish homeologs and said "whereas ROS1 was expressed at the highest level in A suecica". These data do not support conclusions.
Response: As suggested, we revised, "whereas AtROS1 and AaROS1-2 were expressed at high levels in A. suecica (Extended Data Fig. 10a)."

Reply: fine.

L235. "Predict" would be too strong, because there is only correlative evidence on ROS1 and methylation levels.
Response: We toned down and revised it to "speculate".

Reply: fine.

L249 and Fig. 4b. " (Fig. 4b). The number of hyper DMRs was reduced gradually from F1 to Allo733 and Allo738 and dramatically to natural A. suecica". The way of figure presentation for this conclusion is deceiving. I do not think this conclusion is well supported. Allo733 and Allo738 are essentially biological replicates of the synthetic polyploid. It would be fair to show the two values on the same column. Then, the "gradual" pattern is not obvious with only 1 or 2 samples per class. To maintain the conclusion, please provide statistical support.
Response: We agree that Allo733 and Allo738 are biological replicates of resynthesized, which show similar levels of methylation changes. We removed "gradual" or changed to "slowly" or "slightly." "The number of hyper DMRs was reduced slightly from F1 to resynthesized Allo733 and Allo738 and dramatically to natural A. suecica, while the number of hypo DMRs were relatively similar among F1 and resynthesized allotetraploids but increased in A. suecica."

Reply: The text is slightly changed but the figures are unchanged nor statistics are shown. I would suggest to add that Allo733 and Allo738 are biological replicated.

L263 and Fig. 4d. The legend does not explain what the dashed boxes in the figure are.
Response: As suggested, we revised to "Dashed black boxes indicate hypo DMRs between T subgenome and Ath (upper panel) and between A subgenome and Aar (lower panel) in F1 were

conserved in Allo733, Allo738 and Asu."

Reply: fine.

L269-270: how are "convergent" and "conserved" defined? What does the overlap mean? Is this from the line 241?
Response: As suggest, we briefly clarified in the Results added the definition in the Methods. "Conserved DMRs were defined as the hypo DMRs in Asu and consistently present in F1, Allo733 or Allo738. Convergent DMRs were identified as the hyper DMRs between Aar and Ath and in F1 and resynthesized allotetraploids and decreased to a similar level to T subgenome in Asu." The overlap between convergent and conserved groups represented those DMRs convergent in newly formed allotetraploid and remained in Asu (Fig. 4c).
Reply: The definition is fine, but please add the definition in the main text at the first appearance, at least before track line 1015 (Results). There the definition is partially presented but it is used in a specific manner and thus difficult to follow.
In addition, I would like to comment on changes around here.

Track line 853
"The CG hypomethylation was observed in all allotetraploids but more profound in the A subgenome of A. suecica with a sharp reduction of methylation levels in the gene body and flanking 5' and 3' sequences (Mann-Whitney U test: $P < 0.001$) (Fig. 3b), whereas in the T subgenome hypomethylation occurred mainly in the gene body (Mann-Whitney U test: $P > 0.05$)"
P values were updated in the new version. However, it is unclear what was compared with what. Particularly, what is the message of the non-significant value of $P>0.05$?

L313. "predict" is far too strong. All data are a kind of cherry picking and correlative.
Response: We toned down and revised it to, "speculate".

Reply: fine.

Discussion In this section the authors discuss DNA methylation in general terms, but most of the downstream analyses focus on CG methylation changes, meaning that most of the results refer to changes in CG context. This comes back to the comment of Fig. 3a, because not enough context is given to state that CG methylation changes represent the largest amount of changes in the genome. In addition, no DMR analysis was shown for the other two contexts. The global pattern suggests that the amount of DMRs might less, but numbers should confirm that. By better highlighting the importance and abundance of CG methylation changes, the reason behind continuing downstream analyses in CG context only would be more understandable and the discussion section would be better supported. For completeness, a short mention of the other two contexts could be considered as well.
Response: These are valid comments. We added the statistics of CHG and CHH DMRs in Extended Data Fig. 9e. We added this in the Results and also discussed. "CHG hypo DMRs in the A subgenome had a similar trend to CG hypo DMRs that increased slightly in F1 and resynthesized allotetraploids and dramatically in A. suecica, while hypo DMRs in the T subgenome increased dramatically only in A. suecica. CHH hypo DMRs displayed a similar trend to CHG hypo DMRs, except that CHH hyper DMRs had the highest number in the T subgenome among all allotetraploids. Considering that CG

methylation is relatively abundant and stable and correlates with expression levels of DMR-associated genes (Fig. 3e; Extended Data Fig. 9d), we focused most analyses on CG methylation dynamics.”

Reply: fine

Line 320 and 326. The term "ecological distribution" is unclear and unconventional. The reference papers do not seem to explain it. Then, in line 326, the authors discussed "despite diverse ecological distributions". The distribution range of A. suecica is rather narrow in the genus Arabidopsis.
Response: As suggested, we remove the “despite diverse ecological distributions” and revised it to, “A. suecica is estimated to form at 14,000 to 300,000 years ago and distributed in northern Fennoscandia.”
Reply: fine

Methods Method are in general fairly brief. For RNA-seq and MethylC-seq data analysis, further details of mapping should be described. In allopolyploid species, a fragment may often be mapped to two homeologous regions with the same score, and their treatment may lead to errors (for example, Kuo et al. Brief Bioinform. 2020 Mar 23;21(2):395-407; Hu et al. Brief Bioinform. 2020 Mar 27:bbaa035). How were such reads treated?
Response: We are aware of difficulties to handle and map reads in allopolyploids. As the reviewer pointed out and to the best of our knowledge, there is no “perfect” software that is error proof. Our general practice is filter out low-quality reads using Trimmomatic (version 0.39) (Bolger et al., 2014) and map the high-quality reads using variant calling software such as Picard Toolkit (Broad Institute, 2019). SNP tables were generated between subgenomes to partition reads using unique and perfect match. The reads that are mapped onto homoeologs are divided into homoelogs with weighted scores based on the length of reads mapped. We used the same criteria for both mRNA-seq and MethylC-seq data. Detailed procedures have been updated in the Methods.
Reply: fine

L705-706: for reproducibility purposes, these Python scripts should be available. Also, how many cytosines were found to be conserved? This should be stated in the main text to better contextualize the amount of cytosines analyzed
Response: As suggested, Python scripts were included in the Methods and Github (https://github.com/Anticyclone-op/Ara-genome-methly). Statistics of conserved C is given in Source Data Extended Data Fig. 8a, b and some numbers were also mentioned in the main text. We selected conserved C with coverage 3 or more reads and shared among all materials for further analysis. We revised “To improve data reproducibility, we used shared methylation sites (35,853,727) with conserved cytosine and 3 or more reads among different lines for further analysis (Source Data Extended Data Fig. 8a, b).”
Reply: fine

L708-713: a sliding window approach has many limitations, but in the scope of this paper it makes more sense compared to other statistical approaches. One major limitation of sliding windows concerns multiple testing and power, which is something the authors do not seem to address in their methods where a threshold p-value is set, but no multiple testing correction is applied. In addition, the cut-off values of the methylation levels need to be clarified: were these values used as a minimum difference for testing
Response: These are valid comments. Statistical significance was analyzed using Fisher’s¬exact-test

(FDR<0.05), with the following cut-off values of the minimum difference of methylation levels: 0.5 for CG DMRs, 0.3 for CHG DMRs, and 0.1 for CHH DMRs. We clarified this in the Methods.
Reply: fine

Extended Figure 8a and b: an upset plot might be better to show intersections and representing the size of the sets. An alternative would be to have the Venn diagram show circles proportional to the size. Figure b is really hard to understand, in particular what the asterisks refer to and what is the aim of the circle for all genes. There's also no specification of how the overlap between DMRs and genes is defined: is a 1bp overlap enough to be associated to a gene?
Response: As suggested, we replaced Venn plot with upset plot. An asterisk indicates the difference between numbers of the unique CHG or CHH DMRs and their overlapping genes was significantly reduced (Fisher's exact test), indicating CHG or CHH DMRs alone are unlikely associated with genes. DMR overlapping genes were defined as those that were overlapped with DMRs within a 2-kb flanking region.
Reply: add the explanations of the asterisk to the legend. More detailed explanation than the sentence above would be necessary.

Reply: minor comments below are fine.

Minor points
o L38-40: to rephrase. Polyploids do not generate genomic diversity (etc.) in response to selection, domestication or adaptation.
Response: As suggested, we replaced "generate" with "possess."
o L51: typo, "and"
Response: Removed as suggested.
o L144: typo in "allopolyploids"
Response: Corrected as suggested.
o L191-192: not clear how that improves reproducibility.
Response: We replaced "reproducibility" with "comparability", "To improve data comparability, we used shared methylation sites (35,853,727) with conserved cytosine and 3 or more reads among different lines for further analysis (Source Data Extended Data Fig. 8a, b)." and also in the Methods. This should improve comparability and accuracy across different species and possibly avoid variation of sequencing depth and uniformity.
o L233-235: Extended Fig. 7f and g show a slightly higher CHH methylation level in the tetraploids compared to A/T. The pattern is not as strong for Allo733, especially on the A-side where Allo733 has lower CHH levels.
Response: CHH methylation DMRs were rather unstable, probably because siRNAs that induce RdDM are variable in each species. "A similar trend was also observed in the CHG methylation levels of A genome (Extended Data Fig. 8f) and to a lesser degree in the CHH context (Extended Data Figs. 8g)."
o L246-247: Sentence should be less assertive.
Response: We replaced "resulted from" with "accompanied by."
o L270: typo in "pattern"
Response: Corrected as suggested.
o L337: A. arenosa typo
Response: Corrected as suggested.
o L358, 10,00 must be 10,000
Response: Corrected as suggested.

o L386: A. lyrata typo.
Response: Corrected as suggested.
o L684: add some details about sequencing platform and coverage.
Response: "….for mRNA sequencing with three biological replicates each with ~6.5 gigabases per replicate on Illumina HiSeq X Ten platform."
o L694: add some details about sequencing platform and coverage.
Response: "MethylC-seq libraries were constructed using a bisulfite method as previously described (Song et al., 2017) and sequenced on Illumina HiSeq X Ten platform (~11 gigabases per replicate)."
o L700: any reason why the --score_min parameter was adjusted here?
Response: The score threshold was lowered because high heterozygosity in A. arenosa and also the differences between parents, F1, Allo733, and Allo738.
o Legend Fig. 1, lyrate must be lyrata. translation must be translocation.
Response: Corrected as suggested.
o Fig. 1c. The legend is too short to understand what the figure means.
Response: We revised to "Rearrangements between T (sT1-sT5) and A (sA1-sA8) subgenomes of natural Asu and putative progenitors, A. thaliana (Col, T1-T5) and A. arenosa (A subgenome of Allo738 (A1-A8). Ribbons indicate translocations between Ath and A subgenomes (black), within Ath or A subgenome (blue), and in the same chromosomes (red)."
o Extended Fig. 2a: axes unreadable.
Response: The axes were revised as suggested.
o Extended Fig. 3d,e: why are the averages different.
Response: I believe that the question is about different averages of distributions between A and T subgenomes. This could be related to overall differences between DMRs and expression levels of the two subgenomes, as shown in many other allopolyploids such as cotton, oilseed rape, and wheat.
o Extended Fig. 7: keep colors consistent
Response: As suggested, colors were changed to be consistent.
o Extended Fig. 9: ROS2 must be typo. ROS1-1 and ROS1-2?s Differentially expression must be differential expression.
Response: As suggested, we revised to, "Differential expression of methylation pathway genes including AtROS1, AaROS1-1 and AaROS1-2 in allotetraploids."

Reviewer #2 (Remarks to the Author):

The revision of the manuscript by Jiang et al., now entitled "Concerted genomic and epigenomic changes accompany stabilization of Arabidopsis allopolyploids", is substantially improved, and the authors addressed all my major concern, and largely that of the other reviewers. I still find the FLC methylation data (Fig. 2e) not suitable for an interpretation without considering the other elements of its complex regulation, but I can live with that.

The complex revision, changing nearly one third of the text, has unfortunately led to signs of "repair", as new grammar problems and unclear sentences. Just some examples are sentences in l. 111-113, 265-266, 332-334, 363-364, 370-371. And please correct l. 78: "A. arenosa (aka, Care-1)" should probably be "A. arenosa (Aar, Care-1)", l. 103 "(Ara)" should be "(Aar)", l. 387 "240 kp" should be "240 kb". Also in the original text, there are sentences (like the first in the abstract, or l. 419-420, the "right species") that could be made clearer. These minor issues do not decrease the value of the

content but should be erased by a carefully copy editing before publication.

Reviewer #3 (Remarks to the Author):

I am quite satisfied with the response to my feedback. I believe that the changes introduced improved the manuscript and I also find reasonable the reasons why authors prefer to keep a few elements as they are. I would recommend this work for publication in Nature Ecology & Evolution.

Nevertheless, and building upon the changes made, I would suggest a few further improvements to polish the manuscript.

1) I find the nomenclature quite clearer now after stating it at the beginning of the results and after introducing the use of 3 letters (e.g. Asu). However, I would suggest one more modification to improve the consistency. We can see this here: Lines 303-304 “We further analyzed dynamics of hypo and hyper DMRs between Aar (A) and Ath (T)” I think that eliminating this double-naming and calling these Aar/Ath, rather than A/T (mostly in figures) would prevent all the possible ambiguity in the nomenclature. This is something that was already done, for instance, in Figure 3b and in Ext Data fig 8a-8e and I find it clearer.
Therefore, I will recommend to replace A/T by Aar/Ath in these figures:
• Fig 4b -4d (In Fig 4c, keeping the T and A green and purple key on top but replacing the A/T)
• Figure 5a
• Ext Data fig 8f-8g (just for the blue line key, I would keep the T and A on top of each grarph)
• Ext Data fig. 9 (same here, keeping the A and T to refer to (sub)genomes and the Ath, Aar, F1, 733, 338 to refer to the line).
• Ext Data fig 11a,
• Ext Data fig 10a,
Apart from that, in lines 1555-156 AsuT genome is mentioned which I suppose the Authors meant sT. What about Kyo?

2) Regarding the correction of figure 1a. Maybe I would remodel it to avoid ambiguities potentially leading to think that the parents used in this study are the same as the ones that originated. Moreover, It is written “5-6 million years” which is the time estimated for the split of A. thaliana and A. arenosa, but I am not totally sure if it could be misleading and make some people think that it is a date for the hybridization. I have attached a (draft) suggestion of how this figure could look like but the authors should feel free to find any other solution. I hope the authors don’t find it too complicated.

3) I am glad to read in the responses by the authors that, though Homoeologous Exchanges (HE) can happen, they don’t seem to be an important part of the genome. However, I believe that this information should be stated somewhere in the manuscript, so the readers will have no validity doubts about the results concerning the expansion and contraction of subgenomes (are lost genes fallen within an HE?). I believe that the same applies for those rearranged regions (inversion, translocations, etc) with lower Ka/Ks. It should be stated that HE are out of this analyses, in my opinion.

4) Though Wilcoxon rank sum test and Mann-Withney test are the same thing I find preferable to stick to only one of these denominations (e.g. Ext. data fig 8a and line 239).

5) There are also a few minor points concerning the discussion that I would like to review is the discussion part in regard to meiotic genes. First, I think that there is a confusion in line 395 when it is mentioned that

• In line 395 it is mentioned that "… down-regulation of meiosis-related genes such as PDS5 and SMC6… " Based on the text (abstract + results) and extended data fig 12 I thought that those genes were up-regulated.

• In lines 363-364 when it is mentioned that "levels of these three genes and three of homologous genes (SMC1, 363 SMC6B and PDS5B) of SMC3 and PDS5A were reduced". I am not sure if "homologous" is the most precise term. I would simply say that they belong to the same families and are functionally related (Pradillo et al 20015; Palecek and Gruber, 2015; Schubert, 2009).

• In lines 367-368. when it is mentioned that "low expression levels of these genes in A. arenosa are associated with meiotic instability observed in this outcrossing autotetraploids". Is not clear for me whether the authors meant that those expression differences were found in the cited study. In any case, in this work, Yant et al (2013) just identified some genes under selection (including PDS5B and SMC3) in natural A. arenosa tetraploids, which displays a more stable meiosis than newly colchicine-induced tetraploids.

• I am not sure if ASY2 is a relevant/informative case. It seems a very specific gene of A. thaliana (In lyrata there is another ASY1-related ORF, AL1G56910, in a region synthenic with AlASY1, AL2G25920, but it has very little homology with AtASY2). My guess is that it might be paralog of ASY1 that is getting (or already got) pseudogenized in parallel in both species. Given this specificity I am not sure if ASY2 should be in the same category as the genes described by DeSmet et al 2013 (that consistently return to single shortly after independent WGD). Moreover, I couldn't find in the list of genes under selection from Yant et al (2013) the ID of ASY2 (AT4G32200). I could only find ASY1 and ASY3 in the list of the supplemental information of this paper. In any case, if the authors decide to keep this example in the manuscript, I think it would make sense to state in the text (as I understand from the response to my comment) that the heavy methylation and poor expression emerged specifically in allotetraploids and are not observed in the parents.

6) One more silly thing for the proofreading; there are a few texts highlighted in yellow in Figures 2a and 3b.

7) Another tiny suggestion. In line 42 I would say "In A. suecica, the subgenomes sA and sT are divergent enough to prevent from homoeologous exchanges…"

Bibliography.

Pradillo, M., Knoll, A., Oliver, C., Varas, J., Corredor, E., Puchta, H., et al. (2015). Involvement of the cohesin cofactor PDS5 (SPO76) during meiosis and DNA repair in Arabidopsis thaliana. Front. Plant Sci. 6:1034. doi: 10.3389/fpls.2015.01034

Palecek JJ, Gruber S. (2015). Kite Proteins: a Superfamily of SMC/Kleisin Partners Conserved Across Bacteria, Archaea, and Eukaryotes. Structure. 2015 Dec 1; 23(12):2183-2190.

Schubert V. SMC proteins and their multiple functions in higher plants. (2009) Cytogenet Genome Res. 2009; 124(3-4):202-14.

De Smet et al. Convergent gene loss following gene and genome duplications creates single-copy families in flowering plants. 2013 PNAS. 10.1073/pnas.1300127110

Yant, L., Hollister, J.D., Wright, K.M., Arnold, B.J., Higgins, J.D., Franklin, F.C.H., and Bomblies, K. (2013). Meiotic adaptation to genome duplication in Arabidopsis arenosa. Curr Biol 23, 2151-2156.

********************END********************

Author Rebuttal, first revision:

Reviewer #1 (Remarks to the Author):

"Reply:" indicates the comments by reviewers below.

Reviewer #1 (Remarks to the Author):
The authors focused on the genomic and epigenomic analysis of a model allopolyploid species Arabidopsis suecica. The authors first conducted chromosome-scale assembly of natural and synthetic A. suecica. The latter was used as a substitute of a parental species A. arenosa, which I will discuss further below. Standard analyses of synteny and gene families as well as a focus on flowering and self-incompatibility genes follow. The authors did not find a major change that may be called genome shock. Then, the authors reported the main part of this manuscript, genome-wide DNA methylation analysis. Similar to previous studies using cotton, the methylation level of the two subgenomes became similar after hybridization, but in cotton low methylated subgenome became highly methylated in contrast to the decrease of high methylated subgenome in A. suecica. The authors identified differentially methylated regions.
The topic of epigenome of polyploid species is topical, and the dataset of the model allopolyploid A. suecica would be valuable. However, writing should be substantially improved. The methods and figure legends are often too short to follow. The correspondence between the main text and figures are often unclear.
Response: We appreciate the encouraging and constructive analysis of our work from this expert reviewer and have addressed the concerns in this revision.
Reply: Many points were indeed improved. However, I wished the authors took this point more thoroughly: "However, writing should be substantially improved. The methods and figure legends are often too short to follow. The correspondence between the main text and figures are often unclear." The points that are directly pointed out by reviewers are improved but many points indirectly related to them are left. I would mention some of them in the following.

Response: We appreciate the comments from this expert reviewer and have further improved writing and clarity in this revision. To save space, we did not reiterate the "reply" comments with "fine" or acceptable response from the previous review.

A major issue that would need additional analysis is a circular argument about the genome conservation. The authors did not assemble directly the parental species A. arenosa, but the arenosa-derived subgenome of the synthetic A. suecica which experienced about 10 generations after allopolyploidization. Technically, it is a good idea to obtain homozygous state, because high heterozygosity of the autotetraploid A. arenosa would be a major barrier for genome assembly. The authors simply said in line 86 "Because of genome conservation, our further analysis considered the A and T subgenomes of resynthesized Allo738 to be A. arenosa (A) and A. thaliana (T, Ler) genomes, respectively." However, validation and careful interpretation would be necessary. It is not clear what aspect the authors say "genome conservation", or between which individuals the genomes were conserved. In other words, in

this experimental setting, they cannot compare the genome before and after the hybridization (I mean, allopolyploidization). They were comparing the sequence right after allopolyploidization and that after thousands of years.
In DNA sequences, it is no surprise if there were not be major changes in this laboratory 10 generations, if so, the genome may be conserved at the allopolyploidization event. However, there is no direct evidence shown in the manuscript. I would propose a few possibilities to check if the changes in the 10 generations were not huge, although the authors may find additional ways. First of all, at least, the Ler subgenome of synthetic A. suecica should be compared with the Ler genome. Fig. 1c showed a chromosome level synteny with Col accession of A. thaliana, but this is not adequate because the authors discussed smaller scales of changes in the text. There is a publication (Chromosome-level assembly of Arabidopsis thaliana Ler reveals the extent of translocation and inversion polymorphisms. Zapata et al. Proc Natl Acad Sci U S A. 2016 Jul 12;113(28):E4052-60) although I do not know if it is directly usable for your purpose. To compare arenosa subgenome with A. arenosa would be more important. Small amount of long- read data from natural A. arenosa (ideally close to the parent of the synthetic polyploid) may be adequate to validate the assembly. Even when these validations are done, all texts including the abstract, results and discussion should be toned down when it comes to the interpretation of the conservation at the allopolyploidization.
Response: These are valid comments. We thank Dr. Magnus Nordborg for sharing A. arenosa sequence (bioRxiv, Burns et al. 2020). "To test stability of resynthesized Arabidopsis allotetraploid Allo738, we compared the genome of Allo738 with Ler (Zapata et al., 2016) and other Arabidopsis species (Novikova et al., 2016; Novikova et al., 2017) including an A. arenosa accession (https://doi.org/10.1101/2020.08.24.264432), and Allo733 (a sibling of 738) (Extended Data Fig. 3). We found that (1) Allo733 and Allo738 have similar levels of divergence to Ler and Aar4, respectively (Extended Data Fig. 3a); (2) A subgenomes of Allo733 and Allo738 are closely related to A. arenosa accessions that are in a different clade from A subgenomes of A. suecica accessions (Extended Data Fig. 3b); and (3) T subgenome of Allo733 and Allo738 are closely related to Ler, which is different from T subgenome of A. suecica accessions (Extended Data Fig. 3c). Neighbor-joining evolutionary tree also indicated that the A-subgenome donor of Allo733 and Allo738 was closest to A. arenosa (Novikova et al., 2017) (Extended Data Fig. 3b), and T-subgenome donor of Asu was closely related to ecotypes from Russia of Asia admixture of 1135 strains analyzed (Extended Data Fig. 3c) (Consortium, 2016; Novikova et al., 2016). These analyses confirm that A and T subgenomes of resynthesized Allo738 (and Allo733) can be treated as A. arenosa (Aar) and A. thaliana (Ath, Ler) genomes, respectively, for further analysis." We also updated source data with these new analyses.

Reply: I appreciate the substantial effort of validation by the authors, but it only deals with the large scale synteny. This may be fine for epigenetic analysis. However, the validation at more fine scales is lacking, although the assumption that A-subgenome of A. suecica represent A. arenosa sequence probably exist for DNA sequencing analysis. The method section is too short to tell which ones do or do not include this assumption. For example, the Ka/Ks calculations are likely suffered from it. There would be two feasible solutions. First,

validation can be done at a single nucleotide level. For the T-subgenome, it can be straightforward. Is the sequence of the published Ler genome and the newly assembled T-subgenome of the synthetics nearly identical? (If not, it may be interesting but raises new questions). It may not be necessary to evaluate complete genome. Note that phylogenetic tree shown in Extended Dat Fig. 3 is not very informative at a fine scale (it just takes the pattern of majority). Second, the SNPs of the natural arenosa may be used for the Ka/Ks, now that you included such data. Also for analyses other than Ka/Ks, I hope authors would check the validation carefully.

Track Line 2411. "Identification of orthologous genes and Ka/Ks calculations. Orthologous gene clusters were recognized using OrthoFinder 108 (version 2.2.7) using parameters (-S diamond -M msa -T raxml) 109. The single-copy genes of A. thaliana, A. arenosa, A. suecica, and A. lyrata were used to calculate Ks, Ka, and Ka/Ks values 110 by KaKs_Calculator (version 1.2) 111.

In short, I do not think the following statement by the author is valid. " These analyses confirm that A and T subgenomes of resynthesized Allo738 (and Allo733) can be treated as A. arenosa (Aar) and A. thaliana (Ath, Ler) genomes, respectively, for further analysis. "

Response: We appreciate additional comments but respectfully disagree the concluding statement. To address these comments, firstly, we compared fine-scale variation (SNPs) in Ath and Aar in allotetraploids with L*er* and Aar4 from the published data, respectively. At the fine-scale level, we found that frequencies of SNPs and indels were very low (0.04 SNPs and 0.04 indels per kb) in T subgenome between the two resynthesized allotetraploids Allo733 and Allo738, while they were higher (2.15 SNPs and 1.81 indels per kb) in the A subgenome (Source Data Extended Data Fig. 3a); these levels of variation were also comparable with their corresponding extant parents, L*er* and Aar4 (Source Data Extended Data Fig. 3a). As expected, these data suggest that the newly assembled T subgenome of the resynthesized allotetraploids is nearly identical to the published L*er* sequence, in spite of some minor sequence changes.

Secondly, both natural and our reassembled *A. arenosa* genomes have a higher level of SNP variation, which may affect *Ka*/*Ks* values. To address this, we replaced the SNPs of the A subgenome in Allo738 with natural Aar4 and recalculated *Ka* and *Ks* values. The results showed no difference between this new set of data (Aar4 vs. Asu, blue) and the previous result (Aar vs. Asu, orange) for *Ka* (left), *Ks* (middle), and *Ka*/*Ks* (right) values (Response Fig. 1).

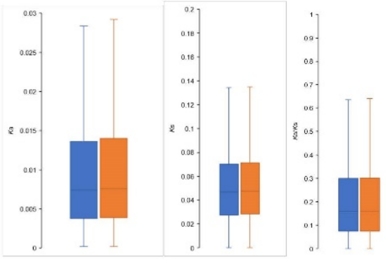

Finally, to satisfy the reviewer's insistence, we removed the word "confirm" and modified the sentence as follows. "For the purpose of this study, we used A and T subgenomes of resynthesized Allo738 (and Allo733) as *A. arenosa* (Aar) and *A. thaliana* (Ath, Ler) genomes, respectively for further analysis." We believe that this is a reasonable compromise based on our good faith effort and results from multiple approaches and analyses. As we noted in the previous response, we did not intend to address population diversity issue in this study. As the reviewer also commented, "This may be fine for epigenetic analysis," which is a main focus of the study.

Track line 605. In a new sentence, "Kyo" appears without explanation. Please explain it. Citing a reference is not enough (readers or reviewers are not expected to spend effort to obtain the reference). From the context, it may be a thaliana "ecotype from Russia of Asia". By the way, "Russia of Asia" must be a typo.

Response: Thanks for the suggestions. Kyo is an ecotype from Kyoto, Japan. We revised to, "…we found that all except one had older LTR insertion events than AsuT. Kyo, an ecotype from Kyoto, Japan, had similar LTR insertion time to the AsuT subgenome (Extended Data Fig. 4h)."

The ecotype from Russia of Asia is a typo and should be Russia-Asia admixture. These accessions were presumably from Russia but belong to the Asia group in the 1001 Genomes Project (https://1001genomes.org/accessions.html). We revised the statement to, "…and T-subgenome donor of Asu was closely related to ecotypes from Russia-Asia admixture among 1135 strains analyzed (Extended Data Fig. 3c) [19,24]."

Extended Data Fig. 1. What is the "proportion" of genes and TEs? Is it per base? If so, how did you classify the genome, gene, TE and intergenic regions?

Response: Correct, the proportion is on per base, which is calculated from the base number of corresponding feature region divided by that of a genomic feature. The genome, gene, TE, and intergenic regions correspond to the whole assembly, coding sequence from 5' UTR to 3' UTR, TE from start to end, and all other regions, respectively. We clarified these in the figure legends.

Legend of Extended Data Fig. 4
The term TLb is used without definition. After spending a while, it turned out that it was defined in the legend of Fig. 1 (translocation between subgenomes). These should be defined in the main text or in each legend separately. This is just a tip of iceberg that makes this manuscript difficult to read through.

Response: To our defense, TLb was defined in the legend (Extended Data Fig. 4c). We double-checked all others.

L117. The authors detected purifying selection, but no conclusions are drawn. There are already a few papers addressing the question whether purifying selection is weaker due to redundancy of homeologs in polyploid species (Capsella bursa-pastoris by Douglas et al. Proc Natl Acad Sci U S A. 2015 Mar 3;112(9):2806-11, A. kamchatica by Paape et al. Nat Commun. 2018 Sep 25;9(1):3909). These papers should be discussed in this context.
Response: That's a valid comment. As suggested, we added a sentence to clarify this. "However, purifying selection is generally weaker due to redundancy of homoeologs in allopolyploids as reported in A. kamchatica (Douglas et al., 2015) and Capsella bursa (Paape et al., 2018), and allopolyploidy might have weakened natural selection because of this bottleneck effect."
Reply: Just a typo (Doublas and Paape are opposite). Otherwise it is fine.

Response: Thanks for careful reading, and we corrected the citation references.

Fig. 2. A. lyrata and A. halleri are clustered and had zero common changes. Many phylogenetic studies including Novikova et al. Nat Genet. 2016 Sep;48(9):1077-82 showed that lyrata and arenosa cluster first, and then halleri comes outside. I hope then the data would make sense.
Response: This is a good comment. We have revisited the analysis of trees, and the result remained unchanged. This could be due to closeness of A. arenosa to A. suecica and a small number of species used in this study. We stated, "Note that clustering between A. lyrata and A. halleri could result from a small number of species used in the study, while A. lyrata and A. arenosa may be more closely related (Novikova et al., 2016)." However, this discrepancy does not affect interpretation of the data.
Reply: The method section is too short (only 5 lines) and so more details would be necessary.

Response: As suggested, we added more details in the Methods. "Single-copy genes of *A. thaliana*, *A. arenosa*, *A. suecica*, *A. lyrata*, and *A. halleri* were extracted using OrthoFinder (version 2.2.7) [108], and parameters (-S diamond -M msa -T raxml) [109] and r8s (version 1.81) were used to estimate divergence time to construct phylogenetic trees [112] with the constrained divergence time range following the TimeTree {Kumar, 2017 #5957}. Contraction and expansion of gene families were identified by CAFE (version 4.2.1) (parameters: -p 0.05 -filter) [113], which accounted for phylogenetic history and provided a statistical basis for evolutionary inference. *P*-values were used to estimate the likelihood of the observed sizes given average rates of gain and loss and used to determine expansion or contraction for individual gene families in each node."

Track line 618.
The text of the original version corresponded to Fig. 2b. However, after revision, new categories were added in the text but the figure is unchanged. Please clarify.

Track line 614 The meaning of "Gene families (744) specific to the A-lineage orthogroups from A. arenosa, A. suecica, A. lyrata, and A. halleri" is not understandable. In Vendiagram

of a or in c, the number 614 is not found. Then, it may mean something else. "A-lineage" of A. suecica can make sence because it has two homeologs. However, why A- or T- lineage matter for A. lyrata and A. halleri? The method section is too short (only 5 lines) and no more information is available. I suspect the authors may mean the clade including A. arenosa, A. suecica A, A. halleri, A. lyrata. However, it is not clear what the meaning of including A. lyrata or A. halleri. In addition, as mentioned before, this tree shape contradicts with a consensus tree of the genus, and so there may be some methodological issues.
Legend if Fig. 2
Black dots indicate node T (ancestor of A. thaliana) is probably wrong. Seeing the brief method, it is likely to be the common ancestor of A. thaliana col and the T-subgenome of A. suecica. The same for Node A.

L155 Extended Data Fig. 5a,b do not seem relevant for the result of the sentence ("gain an exon from AaFLC1"). No figure legend explains a particular exon.
Response: The figure shows gene structure of the longest transcript. We removed the sentence, which is a previously reported result (Nah and Jeffrey Chen, 2010).
Reply: This is fine but another issue is found.

Track line 709.
Regarding the new sentence, no causal evidence was shown. At most, siRNA and DNA methylation "may be involved."

Response: As suggested, the sentence has been revised. "Thus, siRNAs and DNA methylation may also be involved in *FLC* expression and vernalization, in addition to its regulation by long noncoding RNAs [43,44]."

Track line 701
This does not seem to fit to the normal definition of convergent evolution (rather opposite). The authors potentially may mean that As-AaFLC1 and As-AaFLC2 forms a clade, this may be called gene conversion between tandem repeats (which is often called concerted evolution in the case of rDNA evolution). Still, the bootstrap of this clade is only 66, and this alone is not really a strong evidence.

Response: Per suggestion, we changed it to "concerted evolution." "Interestingly, *AaFLC1* and *AaFLC2* in *A. suecica* were clustered in one clade, suggesting concerted evolution (Extended Data Fig. 6a, b)."

L174-184. The description and discussion on the self-incompatible genes should be revised thoroughly. First, the result in the line 178-180 "In A. suecica, the AaSCR allele is silenced by miR867 of SCR04 targeting the first exon of AaSCR with a frameshift mutation (Extended Data Fig. 6d)" was already reported by Novikova et al. (reference 15) and thus it should be cited here. There are indeed new results. It is unclear why "a long-term selection for selfing in the allotetraploid, leading to nonfunctional S-alleles" is suggested. With a single

disruptive mutation in S genes, self-compatibility can evolve, and thus long-term selection is not relevant. More importantly, despite many studies, it has been difficult to detect the selection on self-compatibility from molecular evidences, and there is no molecular evidence in A. suecica supporting selection. A. suecica could have obtained self-compatibility at the origin, then selection was not relevant. Relevant review paper (for example, Shimizu and Tsuchimatsu, Ann Rev Ecol Evol Syst 46, 593-622, 2015) would provide further information. It is unclear what "gradual loss of self-incompatibility" in line 183 means. The sentence indicates no data or reference.
Reply: It is substantially improved.
Track line 762. It is unclear why "the combination of weak alleles" matters. Literally interpreted, it means two weak alleles were combined. That is nothing to do with the mechanism. The authors may mean the weak allele SCR01.

Response: We revised to, "The weak alleles that were immediately silenced by miRNA may contribute to a loss of self-incompatibility in early stage of allotetraploids and become nonfunctional in natural *A. suecica*."

This figure is used to claim that the overall methylation levels were higher in A. arenosa than in A. thaliana, but this might only be true for CG methylation (which appears higher visually). For CHG and CHH methylation there might be an opposite trend, but all the judgement relies on visual inspection. This inspection might also be misleading because the average methylation level needs to be adjusted by the proportion of cytosines in each context, which is usually CHH >> CHG ~= CG. Genome size is also not taken into account. Methylation levels can be defined numerically, and one common approach is to calculate the global methylation level (see Vidalis et al. 2016).
Response: We might not fully understand the comment on "CHH >> CHG ~= CG", as this does not make sense. In the reference cited (Vidalis et al., 2016), "Early methylome sequencing studies of the A. thaliana Columbia reference accession revealed that this model plant methylates about 10.5% of its cytosines globally (30% in context CG, 14% in CHG, and 6% in CHH, approximately) (Zhang et al., 2006; Cokus et al., 2008; Lister et al., 2008)." We estimated global methylation levels and included the data in Source Data Extended Data Fig. 8a, b. The results indicate a similar trend to each (CG, CHG, and CHH) context; the overall methylation levels were higher in A. arenosa than in A. thaliana, especially the CG methylation.
Reply: I probably now found the reason of the confusion in relation to the presentation. Track line 810
Pverall methylation levels were higher in A. arenosa than in A. thaliana (Fig. 3a, Source Data Extended Data Fig. 8a, b)
At a first glance, this conclusion is not visible from these figures. CG appeared higher in Aar, but CHG seems higher in Ath in Extended Data Fig. 8. Then, I noticed that the scale is different between a and b. I would suggest to use the same scale, and add explanation in the main text. Here, the main text asks the readers to compare the values of a and b. The current

presentation may not be incorrect but confusing.

Response: As suggested, we used the same scale for Extended Data Fig. 8a, b. The sentence was revised to "…overall CG methylation levels were higher in *A. arenosa* than in *A. thaliana* (Fig. 3a, Source Data Extended Data Fig. 8a, b).".

L206: not clear how the epigenomic changes are "rapid and persistent" from the previous results. No difference between parents and synthetic polyploid are discussed here, so nothing can be rapid, and there is nothing persistent. This aspect should be addressed later when looking at DMRs and removed here.
Response: This is a good comment. We revised the sentence, "These data suggest dynamic changes of epigenomic modifications in newly formed allotetraploids and natural A. suecica."
Reply: this is better, still what "dynamic" means is obscure. It would be better to define.

Response: This is a good comment, and we revised to "These data suggest that epigenomic modifications are dynamic, which occur largely in CG and CHG sites of natural *A. suecica* and throughout coding sequences including 5' and 3' UTRs of the A subgenome and in the gene body of the T subgenome."

L249 and Fig. 4b. " (Fig. 4b). The number of hyper DMRs was reduced gradually from F1 to Allo733 and Allo738 and dramatically to natural A. suecica". The way of figure presentation for this conclusion is deceiving. I do not think this conclusion is well supported. Allo733 and Allo738 are essentially biological replicates of the synthetic polyploid. It would be fair to show the two values on the same column. Then, the "gradual" pattern is not obvious with only 1 or 2 samples per class. To maintain the conclusion, please provide statistical support.
Response: We agree that Allo733 and Allo738 are biological replicates of resynthesized, which show similar levels of methylation changes. We removed "gradual" or changed to "slowly" or "slightly." "The number of hyper DMRs was reduced slightly from F1 to resynthesized Allo733 and Allo738 and dramatically to natural A. suecica, while the number of hypo DMRs were relatively similar among F1 and resynthesized allotetraploids but increased in A. suecica."

Reply: The text is slightly changed but the figures are unchanged nor statistics are shown. I would suggest to add that Allo733 and Allo738 are biological replicated.

Response: As suggested, we revised the figure legend to "b, Numbers of differentially methylated regions (DMRs) between T subgenome and Ath (Col) or A subgenome and Aar in F_1, 733, 738, and Asu, respectively. Note that Allo733 and Allo738 may be treated as biological replicates of resynthesized allotetraploids." Although sequence variation between the two lines may be small, they are independently lines and may have different epigenetic modifications and phenotypic consequences, as shown by many other studies. Averaging them as biological replicates is probably not a good option. For each sample,

we have combined replicated data for downstream analysis, so the significance test cannot be performed in this figure.

L269-270: how are “convergent” and “conserved” defined? What does the overlap mean? Is this from the line 241?
Response: As suggest, we briefly clarified in the Results added the definition in the Methods. “Conserved DMRs were defined as the hypo DMRs in Asu and consistently present in F1, Allo733 or Allo738. Convergent DMRs were identified as the hyper DMRs between Aar and Ath and in F1 and resynthesized allotetraploids and decreased to a similar level to T subgenome in Asu.” The overlap between convergent and conserved groups represented those DMRs convergent in newly formed allotetraploid and remained in Asu (Fig. 4c).
Reply: The definition is fine, but please add the definition in the main text at the first appearance, at least before track line 1015 (Results). There the definition is partially presented but it is used in a specific manner and thus difficult to follow.

Response: As suggested, we included these definitions in the Results.

In addition, I would like to comment on changes around here.

Track line 853
"The CG hypomethylation was observed in all allotetraploids but more profound in the A subgenome of A. suecica with a sharp reduction of methylation levels in the gene body and flanking 5’ and 3’ sequences (Mann-Whitney U test: $P < 0.001$) (Fig. 3b), whereas in the T subgenome hypomethylation occurred mainly in the gene body (Mann-Whitney U test: $P > 0.05$)"
P values were updated in the new version. However, it is unclear what was compared with what. Particularly, what is the message of the non-significant value of $P>0.05$?

Response: The comparison was made for CG methylation levels of upstream, gene body and downstream respectively between Asu and corresponding parents (Aar/Ath). When $P > 0.05$, it indicates that the decline was mainly in the gene body in T genome at a statistically insignificant level. We clarified them in the revision.

Extended Figure 8a and b: an upset plot might be better to show intersections and representing the size of the sets. An alternative would be to have the Venn diagram show circles proportional to the size. Figure b is really hard to understand, in particular what the asterisks refer to and what is the aim of the circle for all genes. There’s also no specification of how the overlap between DMRs and genes is defined: is a 1bp overlap enough to be associated to a gene?
Response: As suggested, we replaced Venn plot with upset plot. An asterisk indicates the difference between numbers of the unique CHG or CHH DMRs and their overlapping genes was significantly reduced (Fisher’s exact test), indicating CHG or CHH DMRs alone are unlikely associated with genes. DMR overlapping genes were defined as those that were

overlapped with DMRs within a 2-kb flanking region.
Reply: add the explanations of the asterisk to the legend. More detailed explanation than the sentence above would be necessary.

Response: Per suggestion, we added this in the legend (now Figure 9a, b). "An asterisk indicates that the fraction of unique CHG or CHH DMRs (unique / total numbers of DMRs) compared to that of uniquely overlapping genes (unique / total numbers of associated genes) was significantly reduced ($P < 0.05$, Fisher's exact test)."

Reply: minor comments below are fine.

Reviewer #2 (Remarks to the Author):

The revision of the manuscript by Jiang et al., now entitled "Concerted genomic and epigenomic changes accompany stabilization of Arabidopsis allopolyploids", is substantially improved, and the authors addressed all my major concern, and largely that of the other reviewers. I still find the FLC methylation data (Fig. 2e) not suitable for an interpretation without considering the other elements of its complex regulation, but I can live with that.

Response: Thanks for the acceptance of our good faith responses.

The complex revision, changing nearly one third of the text, has unfortunately led to signs of "repair", as new grammar problems and unclear sentences. Just some examples are sentences in l. 111-113, 265-266, 332-334, 363-364, 370-371. And please correct l. 78: "A. arenosa (aka, Care-1)" should probably be "A. arenosa (Aar, Care-1)", l. 103 "(Ara)" should be "(Aar)", l. 387 "240 kp" should be "240 kb". Also in the original text, there are sentences (like the first in the abstract, or l. 419-420, the "right species") that could be made clearer. These minor issues do not decrease the value of the content but should be erased by a carefully copy editing before publication.

Response: Thanks for these comments. We corrected all typos and revised the sentence in the Discussion to, "One possibility is that the new species forms at the right time and under suitable conditions."

Reviewer #3 (Remarks to the Author):

I am quite satisfied with the response to my feedback. I believe that the changes introduced improved the manuscript and I also find reasonable the reasons why authors prefer to keep a few elements as they are. I would recommend this work for publication in Nature Ecology & Evolution.

Nevertheless, and building upon the changes made, I would suggest a few further improvements to polish the manuscript.

1) I find the nomenclature quite clearer now after stating it at the beginning of the results and after introducing the use of 3 letters (e.g. Asu). However, I would suggest one more modification to improve the consistency. We can see this here: Lines 303-304 "We further analyzed dynamics of hypo and hyper DMRs between Aar (A) and Ath (T)" I think that eliminating this double-naming and calling these Aar/Ath, rather than A/T (mostly in figures) would prevent all the possible ambiguity in the nomenclature. This is something that was already done, for instance, in Figure 3b and in Ext Data fig 8a-8e and I find it clearer.

Response: As suggested, we revised the "A/T" to "Aar/Ath".

Therefore, I will recommend to replace A/T by Aar/Ath in these figures:
• Fig 4b -4d (In Fig 4c, keeping the T and A green and purple key on top but replacing the A/T)
• Figure 5a
• Ext Data fig 8f-8g (just for the blue line key, I would keep the T and A on top of each grarph)
• Ext Data fig. 9 (same here, keeping the A and T to refer to (sub)genomes and the Ath, Aar, F1, 733, 338 to refer to the line).
• Ext Data fig 11a,
• Ext Data fig 10a,

Response: As suggested, we replaced A/T with "Aar/Ath" in each figure.

Apart from that, in lines 1555-156 AsuT genome is mentioned which I suppose the Authors meant sT.

Response: Revised as suggested.

What about Kyo?

Response: Kyo is an ecotype from Kyoto, Japan. This is clarified in the revision.

2) Regarding the correction of figure 1a. Maybe I would remodel it to avoid ambiguities

potentially leading to think that the parents used in this study are the same as the ones that originated. Moreover, It is written "5-6 million years" which is the time estimated for the split of A. thaliana and A. arenosa, but I am not totally sure if it could be misleading and make some people think that it is a date for the hybridization. I have attached a (draft) suggestion of how this figure could look like but the authors should feel free to find any other solution. I hope the authors don't find it too complicated.

Response: This is a good suggestion, although it has twisted our arms to squeeze this complication into the limited space of a figure panel. It may raise more questions concerning the formation of natural *A. suecica* via "unreduced gametes" of a diploid Ath and a tetraploid Aar, but we are fine with this explanation.

3) I am glad to read in the responses by the authors that, though Homoeologous Exchanges (HE) can happen, they don't seem to be an important part of the genome. However, I believe that this information should be stated somewhere in the manuscript, so the readers will have no validity doubts about the results concerning the expansion and contraction of subgenomes (are lost genes fallen within an HE?). I believe that the same applies for those rearranged regions (inversion, translocations, etc) with lower Ka/Ks. It should be stated that HE are out of this analyses, in my opinion.

Response: This a good comment. We added, "Notably, the amount of homoeologous exchanges (HEs) between the two subgenomes in Allo738 was relatively small, only ~21.5 kb of Ath origin in the A subgenome and ~1.4 Mb of Aar origin in the T subgenome derived from Aar (Source Data Extended Data Fig. 3a), suggesting a minor role of HE in evolution of allotetraploid *A. suecica* genome." We also stated the HEs are excluded from the analysis of rearranged regions.

4) Though Wilcoxon rank sum test and Mann-Withney test are the same thing I find preferable to stick to only one of these denominations (e.g. Ext. data fig 8a and line 239).

Response: As suggested, we used Mann-Whitney U test.

.

5) There are also a few minor points concerning the discussion that I would like to review is the discussion part in regard to meiotic genes. First, I think that there is a confusion in line 395 when it is mentioned that

• In line 395 it is mentioned that "… down-regulation of meiosis-related genes such as PDS5 and SMC6… " Based on the text (abstract + results) and extended data fig 12 I thought that those genes were up-regulated.

Response: The description was meant to show functional validation in wheat by down-regulation of these genes. "In wheat, down-regulation of meiosis-related genes such as *PDS5* and *SMC6* is sufficient to confer unstable meiotic phenotypes [65]."

• In lines 363-364 when it is mentioned that "levels of these three genes and three of homologous genes (SMC1, 363 SMC6B and PDS5B) of SMC3 and PDS5A were reduced". I am not sure if "homologous" is the most precise term. I would simply say that they belong to the same families and are functionally related (Pradillo et al 20015; Palecek and Gruber, 2015; Schubert, 2009).

Response: Agreed and changed to "…three genes (*SMC1*, *SMC6B* and *PDS5B*) in the same family of *SMC3* and *PDS5A*…"

• In lines 367-368. when it is mentioned that "low expression levels of these genes in A. arenosa are associated with meiotic instability observed in this outcrossing autotetraploids". Is not clear for me whether the authors meant that those expression differences were found in the cited study. In any case, in this work, Yant et al (2013) just identified some genes under selection (including PDS5B and SMC3) in natural A. arenosa tetraploids, which displays a more stable meiosis than newly colchicine-induced tetraploids.

Response: We revised to, "Moreover, some of these genes including *PDS5B* and *SMC3* are highly diverged and under strong selection in *A. arenosa* tetraploids [63]."

• I am not sure if ASY2 is a relevant/informative case. It seems a very specific gene of A. thaliana (In lyrata there is another ASY1-related ORF, AL1G56910, in a region synthenic with AlASY1, AL2G25920, but it has very little homology with AtASY2). My guess is that it might be paralog of ASY1 that is getting (or already got) pseudogenized in parallel in both species. Given this specificity I am not sure if ASY2 should be in the same category as the genes described by DeSmet et al 2013 (that consistently return to single shortly after independent WGD). Moreover, I couldn't find in the list of genes under selection from Yant et al (2013) the ID of ASY2 (AT4G32200). I could only find ASY1 and ASY3 in the list of the supplemental information of this paper. In any case, if the authors decide to keep this example in the manuscript, I think it would make sense to state in the text (as I understand from the response to my comment) that the heavy methylation and poor expression emerged specifically in allotetraploids and are not observed in the parents.

Response: We revised the description to clarify the relevant points. "For example, *ASY2* (asynaptic mutant2), a homolog of *ASY1* [71], is heavily methylated and expressed poorly in Allo733, Allo738, and *A. suecica* and possesses a frameshift mutation, which are not observed in *A. thaliana* or *A. arenosa*. However, these results should be cautiously interpreted as our current data are limited to a few strains and tissue types of biological relevance."

6) One more silly thing for the proofreading; there are a few texts highlighted in yellow in Figures 2a and 3b.

Response: Sorry for the marks that were generated during revision. We fixed those.

7) Another tiny suggestion. In line 42 I would say "In A. suecica, the subgenomes sA and sT are divergent enough to prevent from homoeologous exchanges…"

Response: This should be in line 423. We revised it as suggested.

Bibliography.

Pradillo, M., Knoll, A., Oliver, C., Varas, J., Corredor, E., Puchta, H., et al. (2015). Involvement of the cohesin cofactor PDS5 (SPO76) during meiosis and DNA repair in Arabidopsis thaliana. Front. Plant Sci. 6:1034. doi: 10.3389/fpls.2015.01034

Palecek JJ, Gruber S. (2015). Kite Proteins: a Superfamily of SMC/Kleisin Partners Conserved Across Bacteria, Archaea, and Eukaryotes. Structure. 2015 Dec 1; 23(12):2183-2190.

Schubert V. SMC proteins and their multiple functions in higher plants. (2009) Cytogenet Genome Res. 2009; 124(3-4):202-14.

De Smet et al. Convergent gene loss following gene and genome duplications creates single-copy
families in flowering plants. 2013 PNAS. 10.1073/pnas.1300127110

Yant, L., Hollister, J.D., Wright, K.M., Arnold, B.J., Higgins, J.D., Franklin, F.C.H., and Bomblies, K. (2013). Meiotic adaptation to genome duplication in Arabidopsis arenosa. Curr Biol 23, 2151-2156.

Decision Letter, second revision:

12th May 2021

Dear Jeff,

Thank you for submitting your revised manuscript "Concerted genomic and epigenomic changes accompany stabilization of Arabidopsis allopolyploids" (NATECOLEVOL-201112087B). It has now been seen again by the original reviewer and their comments are below. The reviewers find that the paper has improved in revision, and therefore we'll be happy in principle to publish it in Nature Ecology & Evolution, pending minor revisions to satisfy the reviewers' final requests and to comply with our editorial and formatting guidelines.

If the current version of your manuscript is in a PDF format, please email us a copy of the file in an editable format (Microsoft Word or LaTex)-- we can not proceed with PDFs at this stage.

We are now performing detailed checks on your paper and will send you a checklist detailing our editorial and formatting requirements in about a week. Please do not upload the final materials and make any revisions until you receive this additional information from us.

Thank you again for your interest in Nature Ecology & Evolution. Please do not hesitate to contact me if you have any questions.

Sincerely,

[REDACTED]

Reviewer #1 (Remarks to the Author):

I appreciate the validation of the genome on the page 3 of the point-to-point letter. The authors showed the low difference in the comparison between synthetics in line 113 "At a fine-scale level, we found that frequencies of SNPs and indels were very low (0.04 SNPs and 0.04 indels per kb) in the T subgenome between the two resynthesized allotetraploids Allo733 and Allo738)", although it is not very relevant. The most important validation is the following; "these levels of variation were also comparable with their corresponding extant parents, Ler and Aar4 (Source Data Extended Data Fig. 3a)" (line 116).
However, as far as I read, I could not find any updated methods in relevant sections. I guess that the authors used the data in the file:
12545_2_source_data_131278_qrz528.xlsx, the sheet EDFig.3a.
11,232 SNP (B7 cell) / 118,283,013 (D7 cell) = 9.4E-05
but should I use E7 (M-to-M Alignments) instead of D7?
I agree that the value is low enough. However, I am not sure what M-to-M Alignments means.

Except for this, I am glad that all the points on this exciting manuscript were solved.

Our ref: NATECOLEVOL-201112087B

26th May 2021

Dear Dr. Chen,

Thank you for your patience as we've prepared the guidelines for final submission of your Nature Ecology & Evolution manuscript, "Concerted genomic and epigenomic changes accompany stabilization of Arabidopsis allopolyploids" (NATECOLEVOL-201112087B). Please carefully follow the step-by-step instructions provided in the attached file, and add a response in each row of the table to indicate the changes that you have made. Please also check and comment on any additional marked-up edits we have proposed within the text. Ensuring that each point is addressed will help to ensure that your revised manuscript can be swiftly handed over to our production team.

We would like to start working on your revised paper, with all of the requested files and forms, as soon as possible (preferably within two weeks). Please get in contact with us immediately if you anticipate it taking more than two weeks to submit these revised files.

When you upload your final materials, please include a point-by-point response to any remaining reviewer comments.

If you have not done so already, please alert us to any related manuscripts from your group that are under consideration or in press at other journals, or are being written up for submission to other journals (see: https://www.nature.com/nature-research/editorial-policies/plagiarism#policy-on-duplicate-publication for details).

In recognition of the time and expertise our reviewers provide to Nature Ecology & Evolution's editorial process, we would like to formally acknowledge their contribution to the external peer review of your manuscript entitled "Concerted genomic and epigenomic changes accompany stabilization of Arabidopsis allopolyploids". For those reviewers who give their assent, we will be publishing their names alongside the published article.

Nature Ecology & Evolution offers a Transparent Peer Review option for new original research manuscripts submitted after December 1st, 2019. As part of this initiative, we encourage our authors to support increased transparency into the peer review process by agreeing to have the reviewer comments, author rebuttal letters, and editorial decision letters published as a Supplementary item. When you submit your final files please clearly state in your cover letter whether or not you would like to participate in this initiative. Please note that failure to state your preference will result in delays in accepting your manuscript for publication.

Cover suggestions

As you prepare your final files we encourage you to consider whether you have any images or illustrations that may be appropriate for use on the cover of Nature Ecology & Evolution.

Covers should be both aesthetically appealing and scientifically relevant, and should be supplied at the

best quality available. Due to the prominence of these images, we do not generally select images featuring faces, children, text, graphs, schematic drawings, or collages on our covers.

We accept TIFF, JPEG, PNG or PSD file formats (a layered PSD file would be ideal), and the image should be at least 300ppi resolution (preferably 600-1200 ppi), in CMYK colour mode.

If your image is selected, we may also use it on the journal website as a banner image, and may need to make artistic alterations to fit our journal style.

Please submit your suggestions, clearly labeled, along with your final files. We'll be in touch if more information is needed.

Nature Ecology & Evolution has now transitioned to a unified Rights Collection system which will allow our Author Services team to quickly and easily collect the rights and permissions required to publish your work. Approximately 10 days after your paper is formally accepted, you will receive an email in providing you with a link to complete the grant of rights. If your paper is eligible for Open Access, our Author Services team will also be in touch regarding any additional information that may be required to arrange payment for your article.

Please note that <i>Nature Ecology & Evolution</i> is a Transformative Journal (TJ). Authors may publish their research with us through the traditional subscription access route or make their paper immediately open access through payment of an article-processing charge (APC). Authors will not be required to make a final decision about access to their article until it has been accepted. Find out more about Transformative Journals

<B>Authors may need to take specific actions to achieve compliance with funder and institutional open access mandates. For submissions from January 2021, if your research is supported by a funder that requires immediate open access (e.g. according to Plan S principles) then you should select the gold OA route, and we will direct you to the compliant route where possible. For authors selecting the subscription publication route our standard licensing terms will need to be accepted, including our self-archiving policies. Those standard licensing terms will supersede any other terms that the author or any third party may assert apply to any version of the manuscript.

Please note that you will not receive your proofs until the publishing agreement has been received through our system.

For information regarding our different publishing models please see our Transformative Journals page. If you have any questions about costs, Open Access requirements, or our legal forms, please contact ASJournals@springernature.com.

Please use the following link for uploading these materials:
[REDACTED]

If you have any further questions, please feel free to contact me.

Best regards,

[REDACTED]

On behalf of

[REDACTED]

Reviewer #1:
Remarks to the Author:
I appreciate the validation of the genome on the page 3 of the point-to-point letter. The authors showed the low difference in the comparison between synthetics in line 113 "At a fine-scale level, we found that frequencies of SNPs and indels were very low (0.04 SNPs and 0.04 indels per kb) in the T subgenome between the two resynthesized allotetraploids Allo733 and Allo738)", although it is not very relevant. The most important validation is the following; "these levels of variation were also comparable with their corresponding extant parents, Ler and Aar4 (Source Data Extended Data Fig. 3a)" (line 116).
However, as far as I read, I could not find any updated methods in relevant sections. I guess that the authors used the data in the file:
12545_2_source_data_131278_qrz528.xlsx, the sheet EDFig.3a.
11,232 SNP (B7 cell) / 118,283,013 (D7 cell) = 9.4E-05
but should I use E7 (M-to-M Alignments) instead of D7?
I agree that the value is low enough. However, I am not sure what M-to-M Alignments means.

Except for this, I am glad that all the points on this exciting manuscript were solved.

Author Rebuttal, second revision:

08 June 2021

[REDACTED]
RE: Decision on Nature Ecology & Evolution manuscript NATECOLEVOL-201112087B

Dear ***[REDACTED]***

Thank you for acknowledging the editorial decision on acceptance for our revised manuscript for publication in NEE, pending in response to one remaining comment.

Reviewer #1:
Remarks to the Author:
However, as far as I read, I could not find any updated methods in relevant sections. I guess that the authors used the data in the file:
12545_2_source_data_131278_qrz528.xlsx, the sheet EDFig.3a.
11,232 SNP (B7 cell) / 118,283,013 (D7 cell) = 9.4E-05
but should I use E7 (M-to-M Alignments) instead of D7?
I agree that the value is low enough. However, I am not sure what M-to-M Alignments means.

Except for this, I am glad that all the points on this exciting manuscript were solved.

Response: M-to-M Alignment refers to multiple to multiple alignment (M-to-M alignment), including duplicated regions. For SNP identification, it is inaccurate to use E7 (M-to-M alignments) instead of D7 (one-to-one alignment) for SNP identification. Thus, we identified SNPs in one-to-one alignment regions.

To clarify this, we updated the Method of SNP identification in sections of Assessment of assembly accuracy and Identification of rearrangements and local differences. "Local variants (SNP and indel) were identified in one to one alignment region using the dnadiff function of MUMmer[100]. High-order variation was analyzed using SyRI (version 1.1)[105]."

We updated all files according to the editorial instructions and uploaded them online. If you and editorial office have any questions, please let us know.

Thank you for considering our revised manuscript for publication in *Nature Ecology & Evolution*. We look forward to hearing from you.

Sincerely,

Z. Jeffrey Chen

Final Decision Letter:

24th June 2021

Dear Jeffrey,

We are pleased to inform you that your Article entitled "Concerted genomic and epigenomic changes accompany stabilization of Arabidopsis allopolyploids", has now been accepted for publication in Nature Ecology & Evolution.

Can you please confirm that all sequencing data generated in the study, including raw reads and assembled genomes, are available in the NCBI accession code provided in the Data availability statement.

Before your manuscript is typeset, we will edit the text to ensure it is intelligible to our wide readership and conforms to house style. We look particularly carefully at the titles of all papers to ensure that they are relatively brief and understandable.

The subeditor may send you the edited text for your approval. Once your manuscript is typeset you will receive a link to your electronic proof via email, with a request to make any corrections within 48 hours. If you have queries at any point during the production process then please contact the production team at rjsproduction@springernature.com. Once your paper has been scheduled for online publication, the Nature press office will be in touch to confirm the details.

Acceptance of your manuscript is conditional on all authors' agreement with our publication policies (see www.nature.com/authors/policies/index.html). In particular your manuscript must not be published elsewhere and there must be no announcement of the work to any media outlet until the publication date (the day on which it is uploaded onto our web site).

Please note that <i>Nature Ecology & Evolution</i> is a Transformative Journal (TJ). Authors may publish their research with us through the traditional subscription access route or make their paper immediately open access through payment of an article-processing charge (APC). Authors will not be required to make a final decision about access to their article until it has been accepted. Find out more about Transformative Journals

<B>Authors may need to take specific actions to achieve compliance with funder and institutional open access mandates. For submissions from January 2021, if your research is supported by a funder that requires immediate open access (e.g. according to Plan S principles) then you should select the gold OA route, and we will direct you to the compliant route where possible. For authors selecting the subscription publication route our standard licensing terms will need to be accepted, including our self-archiving policies. Those standard licensing terms will supersede any other terms that the author or any third party may assert apply to any version of the manuscript.

In approximately 10 business days you will receive an email with a link to choose the appropriate

publishing options for your paper and our Author Services team will be in touch regarding any additional information that may be required.

You will not receive your proofs until the publishing agreement has been received through our system.

If you have any questions about our publishing options, costs, Open Access requirements, or our legal forms, please contact ASJournals@springernature.com

An online order form for reprints of your paper is available at https://www.nature.com/reprints/author-reprints.html. All co-authors, authors' institutions and authors' funding agencies can order reprints using the form appropriate to their geographical region.

We welcome the submission of potential cover material (including a short caption of around 40 words) related to your manuscript; suggestions should be sent to Nature Ecology & Evolution as electronic files (the image should be 300 dpi at 210 x 297 mm in either TIFF or JPEG format). Please note that such pictures should be selected more for their aesthetic appeal than for their scientific content, and that colour images work better than black and white or grayscale images. Please do not try to design a cover with the Nature Ecology & Evolution logo etc., and please do not submit composites of images related to your work. I am sure you will understand that we cannot make any promise as to whether any of your suggestions might be selected for the cover of the journal.

You can now use a single sign-on for all your accounts, view the status of all your manuscript submissions and reviews, access usage statistics for your published articles and download a record of your refereeing activity for the Nature journals.

To assist our authors in disseminating their research to the broader community, our SharedIt initiative provides you with a unique shareable link that will allow anyone (with or without a subscription) to read the published article. Recipients of the link with a subscription will also be able to download and print the PDF.

You can generate the link yourself when you receive your article DOI by entering it here: <a href="http://authors.springernature.com/share">http://authors.springernature.com/share<a>.

Yours sincerely,

[REDACTED]

P.S. Click on the following link if you would like to recommend Nature Ecology & Evolution to your librarian http://www.nature.com/subscriptions/recommend.html#forms

** Visit the Springer Nature Editorial and Publishing website at www.springernature.com/editorial-and-publishing-jobs for more information

about our career opportunities. If you have any questions please click here.**
